# Flexible Parametric Accelerated Failure Time Models With Cure

**DOI:** 10.1002/bimj.70074

**Published:** 2025-09-10

**Authors:** Birzhan Akynkozhayev, Benjamin Christoffersen, Xingrong Liu, Keith Humphreys, Mark Clements

**Affiliations:** ^1^ Department of Medical Epidemiology and Biostatistics Karolinska Institutet Stockholm Sweden; ^2^ Department of Oncology‐Pathology Karolinska Institutet Stockholm Sweden

**Keywords:** accelerated failure time models, cure models, flexible parametric models, splines

## Abstract

Accelerated failure time (AFT) models offer an attractive alternative to Cox proportional hazards models. AFT models are collapsible and, unlike hazard ratios in proportional hazards models, the acceleration factor—a key effect measure in AFT models—is collapsible, meaning its value remains unchanged when adjusting for additional covariates. In addition, AFT models provide an intuitive interpretation directly on the survival time scale. From the recent development of smooth parametric AFT models, we identify potential issues with their applications and note several desired extensions that have not yet been implemented. To enrich this tool and its application in clinical research, we improve the AFT models within a flexible parametric framework in several ways: we adopt monotone natural splines to constrain the log cumulative hazard to be a monotonic function across its support; allow for time‐varying acceleration factors, possibly include cure and accommodating more than one time‐varying effect; and implement both mixture and nonmixture cure models. We implement all of these extensions in the rstpm2 package, which is publicly available on CRAN. Simulations highlight a varying success in estimating cure fractions. However, in terms of covariate‐effect estimation, flexible AFT models appear to be more robust than the Cox model even when there is a high proportion of cured individuals in the data, regardless of whether cure is reached within the observed data. We also apply some of our extensions of AFT models to real‐world survival data.

## 1 Introduction

Accelerated failure time (AFT) models with a time‐constant acceleration factor can be defined as $T=T_{0}\exp\left(-\beta^{T}z\right)$, where T is the event time, z are time‐invariant covariates, β is a vector of parameters, and $T_{0}$ is some baseline event time which incorporates random variation. More specifically, the AFT model can be formulated as a log‐linear regression of survival times on covariates:

$$\log\left(t\right)=\mu -\beta^{T}z+\sigma \varepsilon ,$$

where μ is the intercept, σ is the scale, and ε is the random error terms that govern the variability of the survival times. Then, exp(μ+σε) represents the baseline survival time. For instance, if the error term ε is assumed to follow a normal distribution, then the survival times T will follow a log‐normal distribution. Rich families to choose from for the baseline survival function include the generalized gamma and generalized F distributions, which include the Weibull and exponential distributions as special cases (Cox et al. 2007; Cox 2008). AFT models have several strong advantages over proportional hazards models (Crowther et al. 2023). First, they are *collapsible*, meaning that the estimated effect of the exposure of interest will not be affected by adjustment for covariates that are associated with the outcome but not associated with the exposure of interest. Second, AFT models capture proportional changes on the time scale. There has been a recent interest to estimate *restricted mean survival time* (RMST), which can be defined as E(T∧τ|z), where τ is the maximum considered time. Rather than trying to estimate RMST using integration (Parmar and Royston 2011) or pseudo‐values (Andersen et al. 2004), we propose that it is simpler to model E(T) directly using AFT models.

Cox and Oakes (1984) defined a time‐dependent AFT model in terms of a baseline survival function given $S\left(t\right)=S_{0}\left(\int_{0}^{t}\exp\left(-\beta^{T}x\left(u\right)\right)du\right)$, where x(t) is a time‐dependent covariate. We generalize this model by considering x(t) as a time‐dependent encoding for the effect of exposure of time‐constant z. We recently introduced a numerically efficient approach (Crowther et al. 2023), where the cumulative effect is modeled, such that $S\left(t\right)=S_{0}\left(t\phi \left(t\right)\right)$, and then $\exp\left(-\beta^{T}x\left(t\right)\right)=\phi \left(t\right)+t\phi^{\prime }\left(t\right)$; however, this approach does not have a simple interpretation for more than one time‐dependent effect. Pang et al. (2021) recently presented an alternative extension to the time‐dependent AFT model, although it is unclear whether this model can be interpreted in terms of the Cox and Oakes time‐dependent model. To our knowledge, the Cox and Oakes time‐dependent model has not been investigated for flexible parametric AFT models. The model developed by Crowther et al. (2023) represents that baseline log cumulative hazard function using natural splines. The formulation is computationally efficient, as it requires no numerical integration, and a similar approach has been used for a range of flexible parametric models. However, this approach has also been criticized (Fauvernier et al. 2019), as there is no guarantee that the cumulative hazard is monotone increasing across its entire support. We propose a simple extension to allow for monotone natural splines across their entire support.

For practical applications of these methods, it would be useful to understand when the models perform well or perform poorly. Crowther et al. (2023) presented an extensive range of simulations and found that the model had good statistical properties (i.e., low bias and reasonable coverage). However, they did not investigate more challenging data, such as data sets where a proportion of individuals are considered *cured*, meaning that they will never experience the event of interest even with infinite follow‐up time. Cure models account for this subpopulation, making estimation more complex due to the need to distinguish between long‐term survivors and those still at risk.

Recently, Parsa and Van Keilegom (2023) suggested that AFT models may be better suited to modeling cure than are proportional hazards models.

In this paper, we aim to introduce extensions to the flexible parametric AFT models, including the Cox and Oakes time‐dependent model, the use of monotone natural splines, and both mixture and nonmixture cure models. In a mixture cure model, the population consists of a mix of “cured” and “uncured” individuals, where the probability of cure is explicitly modeled. In contrast, the nonmixture cure model assumes all individuals are at risk but constrains the survival function to have a horizontal asymptote at the end of follow‐up, effectively representing a long‐term survival plateau. Through simulations, we assess the performance of these cure models, as well as the performance of AFT models with time‐dependent effects in the presence of cure. Moreover, we demonstrate the application of the AFT model with time‐dependent effects and cure to a synthetic data set.

## 2 Methods

First, recall the time‐dependent AFT model defined by Cox and Oakes (1984), where the survival function $S(t|\bar{X}\left(t\right);\beta ,\gamma )$, cumulative hazard function $H(t|\bar{X}\left(t\right);\beta ,\gamma )$ and hazard function $h(t|\bar{X}\left(t\right);\beta ,\gamma )$ at time t given the history of the covariate vector $\bar{X}\left(t\right)$ to time t are defined by

(1) $$\begin{array}{cc} S(t|\bar{X}\left(t\right);\beta ,\gamma ) & =\exp\left(-H(t|\bar{X}\left(t\right);\beta ,\gamma )\right) \end{array}$$

(2) $$\begin{array}{cc} H(t|\bar{X}\left(t\right);\beta ,\gamma ) & =H_{0}\left(\int_{0}^{t}\exp\left(-\beta^{T}x\left(u\right)\right)du|\gamma \right) \end{array}$$

(3) $$\begin{array}{cc} h(t|\bar{X}\left(t\right);\beta ,\gamma ) & =H^{\prime }\left(t|\bar{X}\left(t\right);\beta ,\gamma \right), \end{array}$$

where $H_{0}\left(\tilde{t}|\gamma \right)$ is the baseline cumulative hazard function at transformed time $\tilde{t}$, β is a vector of parameters for the covariates, x(u) is a design matrix encoding the time‐dependent covariate effect at time u, and γ is a vector of parameters for the baseline functions. Thus, rather than modeling time‐dependent covariates, we implicitly model the time‐dependent acceleration factor. It is also possible, of course, to model time‐dependent covariates using this formulation.

### 2.1 Parameterization for the Time‐Varying Effects

We calculate transformed time using Gauss–Legendre quadrature, such that

$$\begin{array}{c} \tilde{t}=\int_{0}^{t}\exp\left(-\beta^{T}x\left(u\right)\right)\;du\approx \frac{t}{2}\sum_{i=1}^{n}w_{i}\;\exp\left(-\beta^{T}x\left(\frac{t}{2}\left(g_{i}+1\right)\right)\right) \end{array}$$

with quadrature nodes $g_{i}$ and weights $w_{i}$. The current implementation does not support covariates that change with time. However, the above design matrix x(u) could be readily extended to accommodate that case.

Previously, we implemented (Crowther et al. 2023) the integration by expressing it as a cumulative effect, such that

(4) $$\begin{array}{cc} \tilde{t} & =\int_{0}^{t}\exp\left(-\beta^{T}x\left(u\right)\right)\;du=t\exp\left(-\eta \left(\log\left(t\right)|\bar{X}\left(t\right);\tilde{\beta }\right)\right), \end{array}$$

where the linear predictor η is an arbitrary continuous function of time and we chose to model it using natural cubic splines $s(\log\left(t\right)|\tilde{\beta_{2}})$:

$$\begin{array}{cc} \eta (\log\left(t\right)|\bar{X}\left(t\right);\tilde{\beta }) & ={\tilde{\beta_{1}}}^{T}X+\sum_{i\in P}x_{i}s\left(\log\left(t\right)|\tilde{\beta_{2}}\right), \end{array}$$

where X is a covariate matrix, P denotes its subset with time‐varying effects, and $\tilde{\beta }=\tilde{\beta_{1}}⊕\tilde{\beta_{2}}$. Differentiating both sides of (4) with respect to time we then have that the time‐dependent acceleration factor is $\exp\left(-\beta^{T}x\left(t\right)\right)=\exp\left(-\eta \left(\log\left(t\right)|\bar{X}\left(t\right);\tilde{\beta }\right)\right)\left(1-\eta^{\prime }\left(\log\left(t\right)|\bar{X}\left(t\right);\tilde{\beta }\right)\right)$.

The cumulative hazard and hazard functions are then

$$\begin{array}{cc} H(t|\bar{X}\left(t\right);\tilde{\beta },\gamma )=\; & H_{0}\left(t\exp(-\eta \left(\log\left(t\right)|\bar{X}\left(t\right);\tilde{\beta }\right))|\gamma \right)\\ h(t|\bar{X}\left(t\right);\tilde{\beta },\gamma )=\; & h_{0}\left(t\exp(-\eta \left(\log\left(t\right)|\bar{X}\left(t\right);\tilde{\beta }\right))|\gamma \right)\\ & \times \exp\left(-\eta \left(\log\left(t\right)|\bar{X}\left(t\right);\tilde{\beta }\right)\right)\left(1-\eta^{\prime }\left(\log\left(t\right)|\bar{X}\left(t\right);\tilde{\beta }\right)\right). \end{array}$$

Gradients are presented in Appendix A.

### 2.2 Parameterization for the Baseline Function

The baseline functions can be parameterized in a number of ways. For the following development, we propose a linear model for the log cumulative hazard for some basis function B as a function of $\log(\tilde{t})$. We then have that

$$\begin{array}{cc} H_{0}\left(\tilde{t}|\gamma \right) & =\exp(B{\left(\log\left(\tilde{t}\right)\right)}^{T}\gamma )\\ h_{0}\left(\tilde{t}|\gamma \right) & =\exp\left(B{\left(\log\left(\tilde{t}\right)\right)}^{T}\gamma \right)B^{\prime }{\left(\log\left(\tilde{t}\right)\right)}^{T}\gamma )/\tilde{t} \end{array}$$

for transformed time $\tilde{t}$.

### 2.3 Time‐Dependent Covariates and Left Truncation

We can reexpress Equation (3) as

$$\begin{array}{cc} h(t|\bar{X}\left(t\right);\beta ,\gamma ) & =h_{0}\left(\int_{0}^{t}\exp\left(-\beta^{T}x\left(u\right)\right)du)|\;\gamma \right)\exp\left(-\beta^{T}x\left(t\right)\right), \end{array}$$

where $h_{0}\left(\tilde{t}|\gamma \right)=H_{0}^{\prime }\left(\tilde{t}|\gamma \right)$ is the baseline hazard function at transformed time $\tilde{t}$. From this, we see that the definition of h(t|x,β,γ) is dependent on the covariate history from the time origin. For left truncation, survival to time t conditional on survival to delayed entry time $t_{0}$ is

$$\begin{array}{c} S\left(t|\bar{X}\left(t\right);\beta ,\gamma ,t>t_{0}\right)=\frac{S(t|\bar{X}\left(t\right);\beta ,\gamma )}{S(t_{0}|\bar{X}\left(t_{0}\right);\beta ,\gamma )}. \end{array}$$

There may be a difficulty in model interpretation for AFT models with both time‐dependent covariates and delayed entry simultaneously. Obtaining parameter estimates in AFT model with time‐varying exposures assumes knowledge of the full history of the covariates from the time origin to the observed event/censoring time. In the case of left truncated data, this history is commonly unknown prior to a subject's delayed entry time. This will hinder estimation and to proceed will require strong assumptions on past history before delayed entry time.

### 2.4 Log‐Likelihood

Consider observed individuals indexed by i with a data tuple $\left(\delta_{i},t_{0i},u_{i},v_{i},{\bar{X}}_{i}\left(t\right)\right)$, where $\delta_{i}$ is the type of observation, $t_{0i}$ is the date of entry (or left truncation time), $u_{i}$ and $v_{i}$ are other times that vary by type of observation, and ${\bar{X}}_{i}\left(t\right)$ is the history of covariates. For right‐censored data, let δ=0, let $u_{i}$ be the final observed time, and $v_{i}$ is unused. For exactly observed times, let δ=1, let $u_{i}$ be the final observed time, and $v_{i}$ is again unused. For interval‐censored data, let δ=2, let $u_{i}$ be the start of the observed event interval, and $v_{i}$ be the end of the observed event interval. The log‐likelihood for the ith individual is then

$$\begin{array}{cc} l_{i}= & -\left(\delta_{i}=0\right)H\left(u_{i}|{\bar{X}}_{i}\left(u_{i}\right);\beta ,\gamma \right)\\ & +\left(\delta_{i}=1\right)\left(\log\left(h\left(u_{i}|{\bar{X}}_{i}\left(u_{i}\right);\beta ,\gamma \right)\right)-H\left(u_{i}|{\bar{X}}_{i}\left(u_{i}\right);\beta ,\gamma \right)\right)\\ & +\left(\delta_{i}=2\right)\log(\exp\left(-H\left(u_{i}|{\bar{X}}_{i}\left(u_{i}\right);\beta ,\gamma \right)\right)\\ & -\exp(-H\left(v_{i}|{\bar{X}}_{i}\left(v_{i}\right);\beta ,\gamma \right)))\\ & +H(t_{0i}|{\bar{X}}_{i}\left(t_{i}\right);\beta ,\gamma ). \end{array}$$

Gradients for this log‐likelihood can be expressed in terms of log(hazards) and cumulative hazards.

### 2.5 Monotone Natural Splines and Nonmixture Cure Functions

For a linear parameterization on the log cumulative hazard scale, we would prefer the mathematical property that the spline‐based estimate of cumulative hazard is monotonic. It is well‐known that B‐splines are monotone if their parameter vector is also monotone (Ramsay 1988).

A common approach to the calculation of natural splines is to use a null‐space projection based on a QR decomposition (Wang and Yan 2021). Specifically, if we assume that the second derivatives at the boundary knots are zero, then we can: calculate the constraint design matrix $X_{c}$ of the second derivatives at the boundary knots; calculate the full Q matrix from the QR decomposition of $X_{c}^{T}$; drop the first m columns of the Q matrix, where there are m constraints (e.g., m=2), to calculate the reduced Q matrix $Q^{*}$; and then calculate the natural splines at time t using the design matrix N(t) at values t and parameters γ

$$\begin{array}{c} N\left(t\right)\gamma =B\left(t\right)Q^{*}\gamma , \end{array}$$

where B(t) is a B‐spline design matrix. Based on the observation for B‐splines, we can assume that these natural splines will be monotone if $\gamma^{*}=Q^{*}\gamma$ are monotone. For increasing values, we can apply a linear constraint that $\gamma_{i+1}^{*}>\gamma_{i}^{*}$; when $\gamma^{*}$ has length k+1, we can define a difference matrix $\Delta =\left[0|I_{k}\right]-\left[I_{k}|0\right]$, and we have the linear constraint that $\Delta Q^{*}\gamma >0$.

Usefully, the null‐space projection methods based on a QR decomposition for natural splines can be readily extended to *nonmixture cure models* (Jakobsen et al. 2020). A nonmixture cure model will have zero slope after the rightmost boundary knot. In outline, the constraint design matrix $X_{c}$ is defined for the second derivatives at the boundary knots and the first derivative at the right boundary knot. Then there are m= 3 constraints. The calculation of the reduced Q matrix $Q^{*}$ and the natural spline values proceeds in the same manner. Moreover, the constraint to monotone cure models can use a similar linear constraint. In addition, it is possible to derive natural splines from B‐splines avoiding QR decomposition entirely. Wang and Yan (2021) presented an algebraic approach for null‐space projection that takes full advantage of inherent local support properties of B‐splines. We have further advanced this method by adapting it to the nonmixture cure model by introducing an additional constraint on the right boundary and subsequently derived an algebraic solution similarly. For a further extension to algebraic solutions for the null‐space projections, see Appendix B.

### 2.6 Mixture Cure Models

A common representation for cure models is to use a mixture model, which combines the subpopulations of “cured” and “uncured” individuals (Maller and Zhou 1996). Let π(X|θ) represent the probability of cure, also known as the “cure fraction,” where θ is a vector of parameters for the cure fraction, let $S_{u}\left(t|\bar{X}\left(t\right);\beta ,\gamma \right)$ be the survival probability, and $H_{u}\left(t|\bar{X}\left(t\right);\beta ,\gamma \right)$ be the cumulative hazard for the uncured fraction, such that

$$\begin{array}{cc} S(t|\bar{X}\left(t\right);\beta ,\gamma ,\theta ) & =\pi \left(X|\theta \right)+\left(1-\pi \left(X|\theta \right)\right)S_{u}\left(t|\bar{X}\left(t\right);\beta ,\gamma \right)\\ & =\pi \left(X|\theta \right)+\left(1-\pi \left(X|\theta \right)\right)\exp(-H_{u}\left(t|\bar{X}\left(t\right);\beta ,\gamma \right)),\\ H(t|\bar{X}\left(t\right);\beta ,\gamma ,\theta ) & =-\log(S\left(t|\bar{X}\left(t\right);\beta ,\gamma ,\theta \right)),\\ h(t|\bar{X}\left(t\right);\beta ,\gamma ,\theta ) & =\frac{\left(1-\pi \left(X|\theta \right)\right)h_{u}\left(t|\bar{X}\left(t\right);\beta ,\gamma \right)\exp\left(-H_{u}\left(t|\bar{X}\left(t\right);\beta ,\gamma \right)\right)}{S(t|\bar{X}\left(t\right);\beta ,\gamma ,\theta )}. \end{array}$$

We assume that $S_{u}$ and $h_{u}$ follow an AFT model as described in Equations (1) and (3), respectively. The gradients of H(t) and h(t) are presented in Appendix C. We can represent the cure fraction using a logistic model, such that

$$\begin{array}{c} \pi \left(X|\theta \right)=1/(1+\exp\left(-\theta^{T}X\right)). \end{array}$$

## 3 Implementation

For optimization, we used the Broyden–Fletcher–Goldfarb–Shanno (BFGS) algorithm with analytical gradients. The Hessian was calculated from the gradients using finite differences, and the variance–covariance matrix was calculated by inverting the negative Hessian. We used quadratic penalties in the log‐likelihood to ensure that both the baseline splines and the cumulative formulation of time‐varying effects increased monotonically. The models allowed for right censoring, left truncation, and a variety of predictions. See the rstpm2 package on CRAN.

## 4 Simulations

The primary objective of the simulations was to evaluate the performance of the newly proposed extensions for flexible parametric AFT models under various conditions and to compare them to the Cox proportional hazards model. In particular, we aimed to assess the accuracy of model estimates with respect to covariate effects and cure fractions. We examined a range of scenarios, both with and without cure, in the data generation process and in the fitted models. In addition, we assessed the performance of noncure AFT models in cases where no cure is present, as well as more complex cases incorporating both time‐varying effects and cure.

### 4.1 Design

**FIGURE 1:**
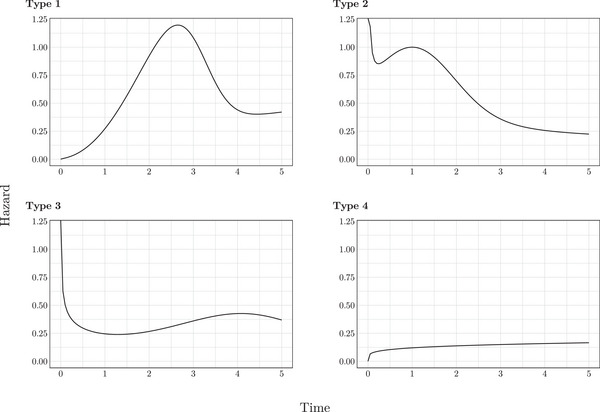
Baseline hazard types for the uncured.

Following Crowther et al. (2023), we modeled survival in the uncured using the four distinct baseline survival functions, with baseline hazard plots provided in Figure 1. Three of these functions were two‐component mixture Weibull functions; the remaining one was a standard Weibull function.

The survival function for the uncured was represented by

(5) $$\begin{array}{ccc} S_{u}\left(t_{i}\right) & = & \omega S_{1}\left(\int_{0}^{t_{i}}\exp\left(-\beta \left(u\right)X_{i}\right)du\right)\\ & & +\;\left(1-\omega \right)S_{2}\left(\int_{0}^{t_{i}}\exp\left(-\beta \left(u\right)X_{i}\right)du\right), \end{array}$$

where ω is a mixing parameter, $S_{1}\left(t\right)$ and $S_{2}\left(t\right)$ are Weibull survival functions with shape parameters $a_{1},a_{2}$, and scale parameters $b_{1},b_{2}$, respectively. For baseline type 1, we used a mixture with parameters $a_{1}=3$, $a_{2}=1.6$, $b_{1}=2.15$, $b_{2}=4.22$; for baseline type 2, $a_{1}=1.5$, $a_{2}=0.5$, $b_{1}=b_{2}=1$; for baseline type 3, $a_{1}=3$, $a_{2}=0.7$, $b_{1}=3.68$, $b_{2}=2.69$; and for baseline type 4, $a_{1}=a_{2}=1.2$, $b_{1}=b_{2}=6.81$. $X_{i}$ is a Bernoulli random variable with a probability of 0.5. In the time‐constant acceleration factor case, the survival function simplifies to

$$S_{u}\left(t_{i}\right)=\omega S_{1}\left(t_{i}\exp\left(-\beta X_{i}\right)\right)+\left(1-\omega \right)S_{2}\left(t_{i}\exp\left(-\beta X_{i}\right)\right)$$

with β set to 1. For the more complex time‐varying acceleration factor case, we used β(u)=2−0.2u. Subsequently, the survival function for the whole population was $S\left(t\right)=\pi +\left(1-\pi \right)S_{u}\left(t\right)$, which represents the mixture of “cured” and “uncured” individuals. Also, note that the survival function in the time‐constant case for the “uncured” individuals $S_{u}\left(t\right)$ was itself the mixture of two Weibull distributions and was specifically chosen to explore the capability of splines in accurately representing and capturing complex shapes within survival data. Consequently, this resulted in a layered mixture framework, where the overall survival model represented a mixture of “cured” and “uncured” populations, and the “uncured” component further involved a mixture of Weibull distributions. We used only a single Weibull baseline in the more complex time‐varying case. For simulation, we first randomly assigned individuals to either being “cured” or “uncured” using a cure fraction π; then for the uncured, we numerically solved the equation $S_{u}\left(t_{i}\right)=u_{i}$ for $t_{i}$ where $u_{i}$ followed a uniform distribution from 0 to 1. For all scenarios, we applied right censoring following an exponential distribution with a rate of 0.1 and a cutoff at 10.

In the time‐constant case, the cure fraction was assigned values of 0, 0.1, 0.5, and 0.9, and, in conjunction with four distinct baseline configurations for the Weibull mixture models, yielded a total of 16 main scenarios.

**FIGURE 2:**
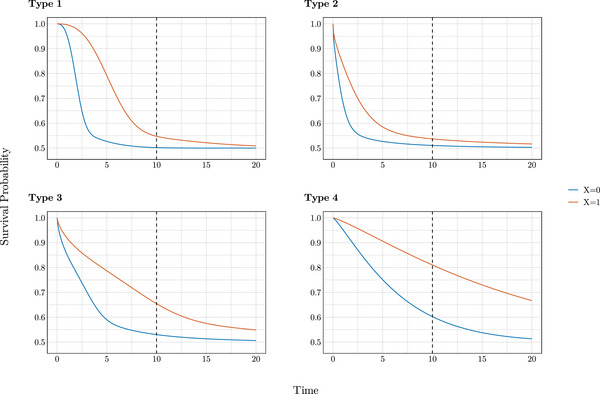
Survival curves under all four baseline scenarios with a cure fraction of 0.5. The dashed line marks the end of follow‐up at 10.

We include a plot of survival curves for a cure fraction of 0.5 (Figure 2) to illustrate how the survival functions behaved in these scenarios. As a result, we observed a gradual transition from type 1, where the survival curve for X=0 (baseline) suggests that cure may be reached within the observed data, to type 4, where this is evidently not the case. The survival curve shapes for other cure fraction values are similar, with the curves plateauing at different levels.

In the time‐varying case, we used a single cure fraction value of 0.9. In total, we generated 1000 data sets for each scenario, with observation counts of 10,000.

In the time‐constant case, we performed model estimation on each of the generated data sets using three types of AFT models: mixture cure, nonmixture cure, and AFT with no cure. The degrees of freedom for the restricted cubic splines in each model fit were configured to span from 2 to 6. In addition, in the special case of baseline type 4—where survival in the uncured is generated using a standard Weibull distribution—we fitted a Cox proportional hazards model. This provided a basis for comparing the AFT and Cox proportional hazards models, since the Weibull distribution is unique in possessing both proportional hazards and AFT properties, meaning it can be expressed equivalently in both modeling frameworks:

$$\begin{array}{c} S_{0}\left(t\exp(-\beta X)\right)=\exp\left(-{\left(\frac{t\exp(-\beta X)}{b_{1}}\right)}^{a_{1}}\right)={\left(S_{0}\left(t\right)\right)}^{\exp(-a_{1}\beta X)}, \end{array}$$

where the log hazard ratio between X=0 and X=1 is then $\beta_{\text{Cox}}=-a_{1}\beta$.

In the time‐varying case, we fitted two types of AFT models: mixture cure with acceleration factors on the cumulative scale; and mixture cure with acceleration factors on the integral scale, following the Cox and Oakes time‐dependent model.

In addition to assessing the goodness of fit of the cumulative hazard function, we calculated the mean squared integrated error (MISE) between the estimated and true cumulative hazard functions from the origin to time points 2, 4, 6, 8, and 10, as follows:

$$\begin{array}{cc} \text{MISE}(t) & =E_{X}\left(\int_{0}^{t}{\left(\hat{H}\left(u|X\right)-H\left(u|X\right)\right)}^{2}du\right)\\ & =\frac{1}{2}\left(\int_{0}^{t}{\left(\hat{H}\left(u|X=0\right)-H\left(u|X=0\right)\right)}^{2}du\right.\\ & \;+\left.\int_{0}^{t}{\left(\hat{H}\left(u|X=1\right)-H\left(u|X=1\right)\right)}^{2}du\right), \end{array}$$

where $\hat{H}\left(t|X\right)$ is the estimated cumulative hazard function for the entire population (including both cured and uncured individuals), and H(t|X) represents the true cumulative hazard function from the data‐generating process.

Following Morris et al. (2019), we reported the relative bias and coverage for the estimates of interest. The relative absolute bias, calculated by taking the absolute values before computing the median, captured the overall magnitude of bias regardless of the direction of the deviation from the true value. We also report on the nonconvergence of the fitting algorithms.

### 4.2 Results

We observed that 83.7% of the AFT model fits converged. Specifically, the convergence rate was 99.2% for noncure AFT models, 89.6% for the nonmixture cure, and the lowest, at 63%, for the mixture cure models. Furthermore, convergence rates declined with increasing degrees of freedom for all model types. None of the mixture cure models fit to baseline type 2 converged for 4 and 5 degrees of freedom. The convergence rates in the time‐varying acceleration factor models were particularly poor for 3 degrees of freedom. The comprehensive tables of simulation results for the converged fits are presented in Tables 1, 2, 3, 4 and 6. These tables include the absolute values of relative bias and coverage for the estimates of both the cure fraction and the covariate effect (log acceleration factor, i.e., β). In addition, they provide Akaike information criterion (AIC) and Bayesian information criterion (BIC) values for model fits, as well as convergence rates. In the tables, we consistently report the medians of values, and for biases, we report the medians of absolute values. In this results section, we aim to clarify our findings by addressing specific questions.

**TABLE 1:** Simulation results for baseline type 1. “Cov.” stands for coverage and “Con.” stands for convergence. AIC and BIC values are represented in thousands.

		Noncure AFT					Mixture cure AFT									Nonmixture cure AFT						
Cure fraction	df	% Bias beta	% Cov. beta	AIC	BIC	% Con.	% Bias beta	% Cov. beta	% Bias cure frac.	% Cov. cure frac.	% Bias surv t=20	% Cov. surv t=20	AIC	BIC	% Con.	% Bias beta	% Cov. beta	% Bias cure frac.	% Cov. cure frac.	AIC	BIC	% Con.
0.0	2	8.1	0.0	27.2	27.2	99.6	6.8	0.0	0.9	0.0	0.9	0.0	27.0	27.0	99.9	0.9	92.5	1.3	0.0	26.3	26.3	99.9
	3	1.0	90.3	26.2	26.2	97.3	0.8	94.0	0.4	0.0	0.4	0.0	26.2	26.2	99.8	0.8	94.8	0.8	0.0	26.2	26.3	99.9
	4	1.3	79.1	26.0	26.0	96.4	1.4	78.8	0.0	0.0	0.3	0.0	26.0	26.0	95.7	1.3	79.2	1.4	0.0	26.1	26.2	98.7
	5	1.0	86.3	25.8	25.9	98.6	1.0	86.8	0.0	0.1	0.0	0.0	25.8	25.9	98.7	1.1	85.2	1.3	0.0	26.0	26.0	96.8
	6	0.7	95.1	25.8	25.8	99.1	0.7	95.1	0.0	0.0	0.0	1.0	25.8	25.8	98.9	0.7	94.5	1.1	0.0	25.9	25.9	97.1
0.1	2	21.0	0.0	29.9	29.9	95.1	6.6	0.6	6.7	68.5	6.7	69.6	27.5	27.5	100.0	1.2	90.5	36.8	0.0	27.1	27.1	99.6
	3	3.2	31.2	26.9	27.0	95.7	1.6	79.9	3.4	94.6	3.4	94.1	26.8	26.9	95.7	1.8	74.4	12.0	29.4	27.0	27.0	99.0
	4	1.4	84.5	26.8	26.8	96.6	1.7	74.7	4.4	89.0	6.0	77.8	26.7	26.7	93.4	1.9	71.8	6.7	66.4	26.8	26.9	99.0
	5	1.1	85.5	26.5	26.5	97.5	1.1	85.1	98.5	100.0	23.8	15.1	26.5	26.6	81.4	1.4	76.8	5.7	79.1	26.6	26.6	98.9
	6	0.8	94.3	26.5	26.5	98.3	0.8	95.0	3.7	96.2	3.7	96.1	26.5	26.5	99.9	0.7	94.2	4.9	82.8	26.5	26.6	95.2
0.5	2	43.7	0.0	24.1	24.1	100.0	6.4	19.7	1.1	92.1	1.1	92.1	21.6	21.6	99.0	5.1	48.7	11.8	0.0	21.8	21.8	100.0
	3	1.9	87.6	21.3	21.4	98.4	2.8	70.1	1.3	81.3	1.3	87.5	21.2	21.3	98.9	2.5	75.9	1.4	82.9	21.3	21.4	99.8
	4	4.2	51.7	21.3	21.4	94.6	2.0	82.5	1.1	91.3	1.1	90.0	21.1	21.2	98.2	2.0	88.7	1.8	72.8	21.4	21.4	96.8
	5	1.2	90.6	21.0	21.1	93.2	1.1	93.6	36.7	96.5	3.3	53.4	21.0	21.1	91.1	1.1	93.7	1.0	92.3	21.1	21.1	97.8
	6	1.2	94.7	21.1	21.1	98.3	1.1	94.3	1.0	95.8	1.0	95.1	21.0	21.1	98.9	1.1	94.1	1.0	92.7	21.1	21.1	96.7
0.9	2	56.9	0.0	7.4	7.4	100.0	6.5	77.6	0.3	94.3	0.3	94.4	6.8	6.8	100.0	9.5	71.0	1.3	24.8	6.9	6.9	100.0
	3	3.3	94.6	6.7	6.8	99.8	4.1	90.7	0.5	82.0	0.3	93.5	6.7	6.7	97.2	3.2	94.1	0.3	93.1	6.7	6.8	100.0
	4	6.9	70.6	6.7	6.8	99.5	5.1	78.1	74.3	98.0	0.4	90.3	6.7	6.7	94.6	5.3	84.6	0.3	93.4	6.7	6.8	99.7
	5	2.7	91.6	6.7	6.7	99.7	2.6	94.0	4.5	99.2	0.4	91.0	6.7	6.7	93.3	2.5	93.8	0.3	94.8	6.7	6.7	99.5
	6	2.9	93.3	6.7	6.7	99.8	2.6	93.5	0.8	98.6	0.4	92.2	6.7	6.7	63.0	2.7	94.1	0.3	95.1	6.7	6.7	99.7

**TABLE 2:** Simulation results for baseline type 2. “Cov.” stands for coverage and “Con.” stands for convergence. AIC and BIC values are represented in thousands.

		Noncure AFT					Mixture cure AFT									Nonmixture cure AFT						
Cure fraction	df	% Bias beta	% Cov. beta	AIC	BIC	% Con.	% Bias beta	% Cov. beta	% Bias cure frac.	% Cov. cure frac.	% Bias surv t=20	% Cov. surv t=20	AIC	BIC	% Con.	% Bias beta	% Cov. beta	% Bias cure frac.	% Cov. cure frac.	AIC	BIC	% Con.
0.0	2	18.7	8.6	12.3	12.4	97.6	18.8	8.6	0.0	21.1	3.5	0.0	12.3	12.3	95.2	2,949.9	0.0	29.2	0.0	65.4	65.4	99.4
	3	3.6	85.9	4.3	4.3	99.6	3.4	87.3	0.0	50.0	0.3	0.0	4.6	4.7	63.8	69,195.0	6.0	29.8	0.0	222.8	222.9	48.1
	4	33.1	0.5	4.0	4.1	99.5										39.4	0.0	3.8	0.0	4.7	4.7	80.9
	5	24.8	0.0	2.3	2.4	100.0										26.3	0.0	5.1	0.0	7.3	7.4	40.6
	6	15.6	0.5	1.8	1.8	99.7	13.6	7.1	0.0	35.7	1.0	0.0	5.5	5.5	1.4	19.6	0.0	4.4	0.0	5.8	5.8	48.5
0.1	2	9.4	68.6	13.6	13.7	99.9	9.5	68.5	100.0	99.1	18.2	24.4	13.6	13.7	99.9	3,005.5	0.0	231.5	0.0	60.1	60.1	99.8
	3	8.0	56.4	6.8	6.8	99.8	5.2	84.8	100.0	89.7	65.9	0.0	2.0	2.1	16.5	21.1	2.4	77.1	0.0	8.5	8.6	75.7
	4	15.7	32.0	6.8	6.8	99.6										52.5	0.2	35.7	0.0	7.3	7.4	47.3
	5	31.4	0.0	5.1	5.1	99.4										29.9	0.2	45.0	0.0	7.8	7.8	46.2
	6	17.5	0.1	4.6	4.6	99.1	18.5	0.0	100.0	100.0	6.3	81.0	4.7	4.7	2.0	21.0	0.0	40.0	0.0	7.1	7.1	48.2
0.5	2	17.5	60.4	14.7	14.7	100.0	17.0	62.1	100.0	99.6	15.0	0.6	14.6	14.6	98.1	3,009.0	0.0	9.1	0.0	38.6	38.6	98.6
	3	34.7	1.7	11.7	11.7	99.9	10.5	0.0	6.2	0.0	6.2	0.0	21.6	21.6	0.1	8.2	65.9	4.8	4.4	11.3	11.3	99.2
	4	36.2	0.1	11.2	11.2	99.3	0.5	100.0	99.5	100.0	4.4	0.0	21.5	21.6	0.1	19.2	40.9	4.7	6.9	12.0	12.1	89.3
	5	39.1	0.1	10.6	10.6	97.2	3.8	100.0	2.6	100.0	4.4	0.0	21.5	21.5	0.1	30.8	0.2	5.4	0.0	11.6	11.6	45.8
	6	17.5	9.1	10.3	10.4	98.8	3.9	100.0	2.9	100.0	4.5	0.0	21.5	21.5	0.1	20.3	5.1	4.6	2.4	11.2	11.3	45.4
0.9	2	41.0	63.1	5.9	5.9	100.0	30.3	76.1	100.0	99.7	2.3	23.2	5.9	5.9	100.0	3,047.3	5.6	1.3	20.6	10.1	10.2	99.5
	3	49.7	30.3	5.4	5.4	100.0	10.8	79.1	0.4	87.6	0.4	78.0	7.4	7.4	13.8	26.6	44.7	0.4	77.2	5.4	5.4	95.6
	4	28.2	33.3	5.2	5.3	99.8	6.2	97.0	23.4	99.2	0.3	95.5	7.4	7.4	13.2	20.0	60.2	0.4	78.7	5.3	5.4	95.2
	5	36.5	20.1	5.2	5.2	99.7	7.1	94.6	0.4	99.1	0.3	95.5	7.4	7.4	11.1	28.3	22.3	0.6	65.5	5.3	5.3	99.1
	6	15.8	53.5	5.1	5.2	99.7	6.3	93.9	18.4	99.0	0.3	95.9	7.3	7.4	9.8	18.4	47.1	0.5	73.9	5.2	5.3	99.8

**TABLE 3:** Simulation results for baseline type 3. “Cov.” stands for coverage and “Con.” stands for convergence. AIC and BIC values are represented in thousands.

		Noncure AFT					Mixture cure AFT									Nonmixture cure AFT						
Cure fraction	df	% Bias beta	% Cov. beta	AIC	BIC	% Con.	% Bias beta	% Cov. beta	% Bias cure frac.	% Cov. cure frac.	% Bias surv t=20	% Cov. surv t=20	AIC	BIC	% Con.	% Bias beta	% Cov. beta	% Bias cure frac.	% Cov. cure frac.	AIC	BIC	% Con.
0.0	2	14.2	6.4	30.2	30.2	99.8	14.3	6.4	0.0	66.6	1.5	0.0	30.2	30.3	99.5	50.6	0.0	27.2	0.0	35.2	35.2	39.4
	3	9.9	22.7	29.4	29.4	99.5	5.1	58.5	1.4	0.0	1.6	0.0	30.5	30.5	20.7	22.4	0.1	17.0	0.0	30.3	30.3	91.1
	4	5.4	50.8	29.2	29.2	99.3	2.2	92.5	5.8	0.0	5.8	0.0	30.3	30.4	20.7	1.7	91.0	7.6	0.0	29.2	29.3	89.2
	5	4.3	62.7	29.1	29.1	98.5	2.2	90.1	5.3	0.0	5.3	0.0	30.3	30.4	20.5	1.8	92.2	7.2	0.0	29.3	29.3	83.3
	6	2.2	87.4	29.0	29.1	98.6	1.7	90.8	3.5	0.0	3.6	0.0	30.3	30.3	19.6	1.7	92.7	7.2	0.0	29.4	29.5	60.1
0.1	2	8.5	43.8	29.4	29.4	100.0	8.8	41.1	100.0	81.0	48.0	1.0	29.4	29.4	99.9	50.7	0.0	230.1	0.0	33.3	33.3	46.7
	3	4.6	75.1	28.7	28.7	99.6	5.7	64.9	9.5	84.5	11.0	77.0	29.7	29.7	26.5	20.6	0.0	134.5	0.0	29.2	29.2	96.6
	4	5.1	68.9	28.6	28.7	99.2	2.2	89.0	51.7	0.4	51.6	0.0	29.5	29.6	26.7	2.5	83.9	59.0	0.0	28.5	28.5	94.8
	5	6.2	42.5	28.4	28.5	98.8	2.4	95.3	47.0	0.0	47.1	0.0	29.5	29.6	26.0	1.9	96.7	59.1	0.0	28.6	28.6	85.8
	6	2.3	88.8	28.3	28.4	98.3	2.2	93.5	39.3	37.6	33.7	30.6	29.5	29.5	24.5	1.7	96.1	61.0	0.0	28.7	28.7	68.8
0.5	2	8.4	68.3	21.5	21.5	99.8	6.3	83.7	17.3	51.4	11.4	8.7	21.5	21.5	93.6	51.3	0.0	20.6	0.0	23.1	23.2	77.0
	3	8.1	72.4	21.2	21.2	100.0	7.5	74.4	1.4	96.9	1.4	95.8	21.7	21.7	47.7	14.6	23.7	9.7	0.0	21.3	21.3	98.5
	4	11.0	61.0	21.2	21.2	98.8	3.4	93.4	5.6	8.6	5.6	8.6	21.6	21.6	48.6	4.5	84.0	6.1	4.6	21.2	21.2	98.7
	5	7.3	62.9	21.0	21.0	99.6	6.2	73.8	92.4	65.0	4.6	38.4	21.6	21.6	46.6	3.5	92.7	6.4	3.2	21.5	21.6	70.8
	6	3.2	93.5	21.0	21.1	97.8	3.2	93.5	61.4	92.2	1.8	85.2	21.6	21.6	38.5	3.1	95.0	6.6	1.8	21.4	21.5	80.5
0.9	2	22.6	69.2	6.5	6.6	100.0	13.7	89.1	1.4	81.7	1.0	70.9	6.5	6.5	100.0	50.3	6.8	1.6	5.1	6.8	6.8	100.0
	3	13.4	91.7	6.5	6.6	100.0	11.2	90.0	0.4	97.4	0.4	97.0	6.5	6.6	86.1	11.8	83.1	0.8	64.9	6.5	6.5	99.6
	4	18.0	75.1	6.5	6.5	100.0	10.5	86.7	0.8	90.7	0.8	73.0	6.5	6.6	81.6	9.6	91.2	0.6	77.7	6.5	6.6	97.8
	5	9.6	87.4	6.5	6.5	100.0	9.8	88.6	88.2	97.7	0.5	89.8	6.5	6.6	81.4	8.7	92.3	0.7	77.1	6.5	6.6	99.9
	6	8.3	91.5	6.5	6.5	100.0	7.7	91.3	20.3	99.1	0.4	96.4	6.5	6.6	64.7	8.4	93.2	0.7	74.0	6.5	6.6	99.2

**TABLE 4:** Simulation results for baseline type 4. 'Cov.' stands for coverage and 'Con.' stands for convergence. AIC and BIC values are represented in thousands

		Noncure AFT					Mixture cure AFT									Nonmixture cure AFT						
Cure fraction	df	% Bias beta	% Cov. beta	AIC	BIC	% Con.	% Bias beta	% Cov. beta	% Bias cure frac.	% Cov. cure frac.	% Bias surv t=20	% Cov. surv t=20	AIC	BIC	% Con.	% Bias beta	% Cov. beta	% Bias cure frac.	% Cov. cure frac.	AIC	BIC	% Con.
0.0	2	1.9	95.3	25.6	25.6	100.0	1.9	95.2	0.4	0.2	3.1	0.0	25.6	25.6	99.8	16.4	0.3	39.6	0.0	28.5	28.5	99.4
	3	2.0	95.7	25.6	25.6	100.0	2.0	95.8	0.7	0.0	3.2	0.0	25.6	25.7	99.3	3.5	71.8	27.6	0.0	26.6	26.7	99.7
	4	2.1	95.5	25.6	25.7	99.9	2.1	95.4	1.6	0.0	3.9	0.0	25.6	25.7	97.1	3.9	73.9	23.7	0.0	26.3	26.4	99.7
	5	2.1	95.1	25.6	25.7	100.0	2.1	95.1	3.6	0.0	5.1	0.0	25.6	25.7	95.7	2.4	94.2	22.7	0.0	26.2	26.3	99.5
	6	2.1	95.2	25.6	25.7	100.0	2.1	95.8	3.9	0.0	5.3	0.0	25.6	25.7	90.0	2.1	95.8	22.3	0.0	26.2	26.2	99.6
0.1	2	2.5	93.5	24.2	24.2	100.0	2.2	95.9	19.7	94.7	24.8	76.9	24.2	24.2	100.0	16.2	0.5	348.8	0.0	26.6	26.6	99.4
	3	2.2	96.1	24.2	24.2	100.0	2.2	96.3	28.8	92.5	27.3	78.6	24.2	24.2	99.8	3.0	81.0	241.3	0.0	25.0	25.1	99.9
	4	2.2	96.4	24.2	24.2	100.0	2.2	96.0	53.5	86.2	32.1	78.6	24.2	24.2	96.7	3.9	79.1	209.6	0.0	24.8	24.8	99.8
	5	2.1	95.7	24.2	24.2	100.0	2.1	95.6	59.6	84.6	32.7	81.2	24.2	24.2	96.8	2.5	94.0	201.7	0.0	24.7	24.8	99.9
	6	2.2	95.1	24.2	24.2	100.0	2.2	95.4	51.7	82.6	34.2	81.5	24.2	24.2	95.5	2.2	95.3	198.3	0.0	24.7	24.7	99.9
0.5	2	7.7	68.1	16.4	16.5	100.0	3.3	95.9	3.3	95.2	3.4	88.6	16.4	16.4	99.7	15.2	12.9	35.7	0.0	17.5	17.6	100.0
	3	3.6	96.5	16.4	16.4	100.0	3.3	96.1	4.6	94.1	3.9	88.2	16.4	16.4	99.2	3.0	93.3	24.7	0.0	16.9	16.9	100.0
	4	3.2	95.2	16.4	16.4	100.0	3.5	95.7	10.9	83.2	10.1	72.0	16.4	16.4	34.6	4.2	89.2	22.2	0.0	16.8	16.8	99.9
	5	3.3	96.1	16.4	16.4	100.0	3.4	94.0	65.6	82.4	11.9	69.9	16.3	16.4	31.9	3.7	95.2	21.6	0.0	16.7	16.8	99.9
	6	3.4	95.9	16.4	16.4	100.0	3.9	93.3	70.7	95.7	11.2	85.6	16.4	16.4	29.9	3.4	95.1	21.5	0.0	16.7	16.8	99.9
0.9	2	12.9	82.9	4.7	4.7	100.0	8.1	96.3	0.9	95.1	0.8	93.1	4.6	4.7	99.2	15.3	69.6	3.7	0.0	4.9	4.9	100.0
	3	9.0	95.9	4.6	4.7	100.0	8.7	93.7	1.1	90.4	1.1	80.5	4.5	4.6	33.8	7.1	95.7	2.6	0.2	4.8	4.8	99.9
	4	8.4	96.2	4.6	4.7	100.0	8.1	96.0	61.3	96.3	2.0	70.6	4.6	4.6	40.5	8.2	95.2	2.4	1.1	4.8	4.8	100.0
	5	8.6	95.7	4.6	4.7	100.0	8.6	95.8	60.1	96.9	1.7	77.8	4.6	4.7	45.0	9.4	93.7	2.4	1.2	4.8	4.8	99.9
	6	8.5	94.6	4.6	4.7	100.0	8.5	95.1	66.8	97.5	1.5	80.8	4.6	4.7	48.9	9.1	92.8	2.4	1.6	4.8	4.8	100.0

**TABLE 5:** Comparison of Cox PH and noncure AFT (df = 2 and 6) in estimating log hazard ratios (Cox PH) and log acceleration factors (AFT) for baseline type 4 (standard Weibull). AIC and BIC values are represented in thousands.

	Cox PH					Noncure AFT, df = 2					Noncure AFT, df = 6				
Cure fraction	% Bias beta	% Cov. beta	AIC	BIC	% Con.	% Bias beta	% Cov. beta	AIC	BIC	% Con.	% Bias beta	% Cov. beta	AIC	BIC	% Con.
0.0	2.0	95.6	65.0	65.0	100.0	1.9	95.3	25.6	25.6	100.0	2.1	95.2	25.6	25.7	100.0
0.1	5.5	56.3	58.8	58.8	100.0	2.5	93.5	24.2	24.2	100.0	2.2	95.1	24.2	24.2	100.0
0.5	20.4	0.2	33.4	33.4	100.0	7.7	68.1	16.4	16.5	100.0	3.4	95.9	16.4	16.4	100.0
0.9	29.3	12.5	6.8	6.8	100.0	12.9	82.9	4.7	4.7	100.0	8.5	94.6	4.6	4.7	100.0

**TABLE 6:** Simulation results for time‐varying acceleration factors. “Cov.” stands for coverage and “Con.” stands for convergence. AIC and BIC values are represented in thousands.

	Acceleration factor on cumulative scale									Cox and Oakes time‐varying AFT								
df	% Con.	% Bias cure frac.	% Cov. cure frac.	AIC	BIC	time	% Bias acc. factor	% Cov. acc. factor	MISE	% Con.	% Bias cure frac.	% Cov. cure frac.	AIC	BIC	time	% Bias acc. factor	% Cov. acc. factor	MISE
2	98.4	0.2	96.6	4.6	4.6	2	10.0	95.5	0.0	95.3	0.1	94.3	4.6	4.6	2	0.5	94.3	0.0
						4	5.1	84.9	0.1						4	0.6	95.2	0.1
						6	12.2	89.7	0.6						6	1.5	95.7	0.6
						8	24.9	92.3	1.8						8	1.1	94.1	1.8
						10	39.4	71.8	4.8						10	0.1	94.0	4.8
3	43.6	0.4	90.8	4.6	4.7	2	13.2	95.0	0.0	17.1	1.0	93.1	4.6	4.7	2	3.6	92.5	0.0
						4	4.0	77.5	0.1						4	2.7	91.4	0.1
						6	10.1	81.9	0.6						6	2.7	93.1	0.6
						8	23.0	91.5	1.8						8	2.2	89.6	1.8
						10	37.2	75.2	4.8						10	0.0	89.7	4.8
4	74.5	14.4	89.3	4.6	4.6	2	12.1	96.3	0.0	73.3	1.4	85.3	4.6	4.6	2	1.7	94.7	0.0
						4	5.0	84.0	0.1						4	0.3	95.5	0.1
						6	12.3	88.7	0.6						6	0.3	95.8	0.6
						8	24.5	92.7	1.8						8	0.7	94.2	1.8
						10	38.8	73.3	4.8						10	1.0	94.8	4.8
5	66.8	59.2	93.9	4.6	4.6	2	11.2	95.1	0.0	60.1	45.8	87.0	4.6	4.6	2	0.0	94.7	0.0
						4	4.4	80.9	0.1						4	1.4	95.1	0.1
						6	11.3	84.0	0.6						6	1.5	96.9	0.6
						8	24.1	92.4	1.8						8	1.1	92.8	1.8
						10	38.2	73.4	4.8						10	3.6	94.2	4.8
6	79.3	9.3	83.3	4.6	4.7	2	9.7	96.2	0.0	78.2	4.9	83.6	4.6	4.7	2	0.0	94.7	0.0
						4	4.4	81.9	0.1						4	1.4	94.9	0.1
						6	11.8	88.5	0.6						6	0.7	94.5	0.6
						8	24.5	92.4	1.8						8	1.1	93.5	1.8
						10	39.0	71.5	4.8						10	0.7	94.5	4.8

#### 4.2.1 Estimation of Covariate Effect

Scenario 2 proved particularly challenging: even when there was no cure in the data, the estimates for β were moderately biased. By contrast, in the remaining scenarios all AFT models—whether adjusted for cure or not—performed well in estimating the covariate effect provided at least 3 degrees of freedom were used, with relative bias capped at about 18%.

Notably, the AFT models reliably estimated covariate effects even under scenarios with high cure fractions, without explicitly modeling for cure as illustrated in Tables 1, 2, 3, 4. Cure for the baseline is largely observed within data for Scenario 1, whereas substantial hazards are estimated toward the end of follow‐up for Scenarios 3 and 4. For these scenarios, we observe that bias in covariate effect estimation increases with both the cure fraction and the extent to which the cure point lies beyond the follow‐up period. Nevertheless, the cure‐unadjusted AFT model maintained low bias and decent coverage. Indeed, as shown in Table 5, a noncure AFT model with only 2 degrees of freedom still outperformed the Cox model. Increasing the degrees of freedom further improved the model fit. In this case, where we compared the Cox and noncure AFT model, cure was not reached within the observed data, as illustrated in Figure 2. As previously noted in the design, this case provides an even playing field for both models, as the simple Weibull distribution satisfies both the proportional hazards and AFT assumptions.

The performance in estimating acceleration factors under the time‐varying scenario was generally good, with bias ranging from near zero to a maximum of 24%, as shown in Table 6. The Cox and Oakes integrated model outperformed the cumulative‐scale acceleration factor model, with a maximum bias in the acceleration factor of 3.8%.

#### 4.2.2 Estimation of Cure Fraction

Overall, the mixture cure model generally performed poorly in estimating cure fractions, although there were a few instances of good accuracy, rendering its performance inconsistent. We also observed poor coverage for estimates of the cure fraction in both mixture and nonmixture cure models, particularly for low cure values. This may be due to the estimates lying at the boundary of the parameter space, thereby rendering Hessian‐based inference inappropriate. In addition, because a logit link is used for the cure fraction (a strictly positive function), achieving coverage when the true cure fraction is exactly or nearly zero is unattainable. On the other hand, the nonmixture cure model showed excellent performance for higher cure fractions of 0.5 and 0.9 but was much less effective in estimating lower cure fractions of 0.1 and 0.

The estimation of the cure fraction was generally accurate in time‐varying acceleration factor cases (Table 6), except when the degrees of freedom were 5. Bias in the cure fraction was estimated more accurately using the Cox and Oakes integrated model compared to the cumulative model, with relative bias reaching a maximum of 4.9%, excluding the case where the degrees of freedom were 5, where the bias was 45.8%. Coverage was reasonable, decreasing from 94.3% to 83.6% as the degrees of freedom increased.

### 4.3 Commentary on Results

Now, we provide a rationale for why a flexible AFT model with sufficient degrees of freedom performs well in cure data sets, even without explicitly adjusting for cure. We illustrate this by fitting the model to a randomly selected data set from Scenario 1, where the cure may have been reached within the follow‐up period, and comparing it to the true generating process.

**FIGURE 3:**
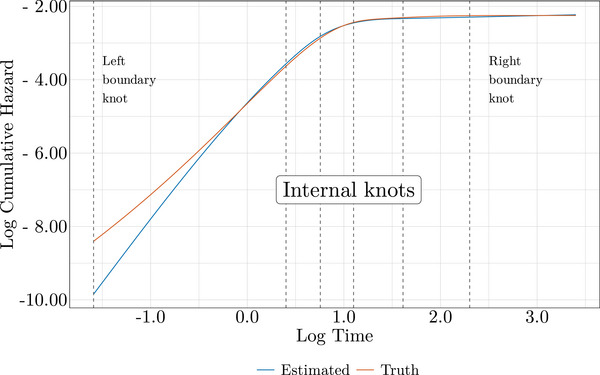
Noncure AFT fit for Scenario 1 with a cure fraction of 0.9: Log cumulative hazard on the log time scale, comparing estimated and true generating processes.

**FIGURE 4:**
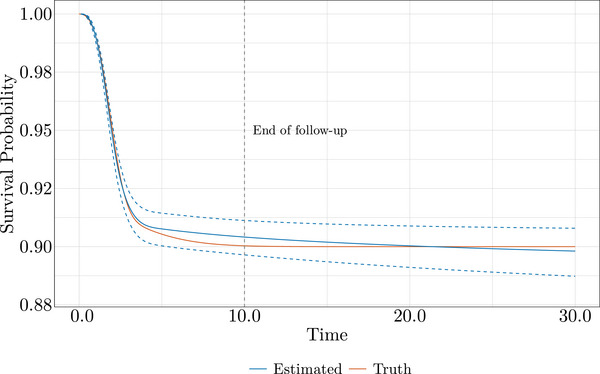
Noncure AFT fit for Scenario 1 with a cure fraction of 0.9: Survival curves comparing estimated and true generating processes.

In Figure 3, we observe the core of the model fit, where the estimation process is primarily shaped, on the log cumulative hazard and log time scale. The log cumulative hazard is already approaching its asymptote, and the last two spline knots effectively control the estimated log cumulative hazard, flattening it even in the absence of an explicitly modeled cure. This behavior translates to Figure 4, where survival is accurately estimated even far beyond the observed data. If the cure is achieved within the data, a flexible AFT model with sufficient degrees of freedom could be useful not only for estimating covariate effects but also for predicting survival beyond the observed follow‐up period.

This characteristic also partially explains the challenge in fitting the mixture cure model. The cumulative hazard can appear nearly bounded above even without explicitly modeling a cure fraction, due to the flexibility of the splines. We suspect this contributes to the presence of local optima, making optimization more difficult. When applying the mixture cure model with different initial values to the same data set from Scenario 1 with a cure fraction of 0.9, we observed sensitivity to the choice of initial values. The BFGS algorithm got stuck in local optima, converging to a cure fraction of 0.36 in one case and 0.9 in the other.

In addition, we observed a high correlation between the coefficients associated with spline functions and those of the cure fraction. This also might indicate that the curvature inherent in the splines may have already accounted for the aspects typically captured by the cure fraction component.

**FIGURE 5:**
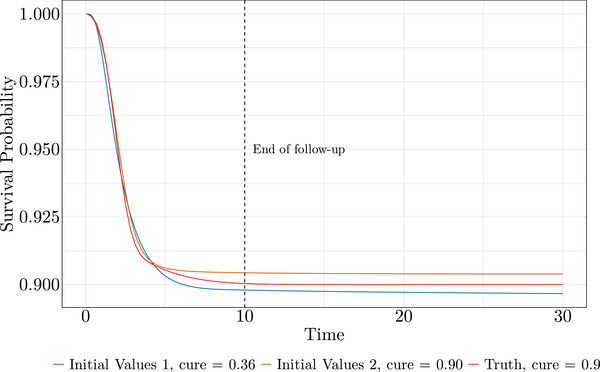
Survival curves for the same data set from two fits of the model using different initial values, compared with the true generating process.

Nevertheless, a fit that converges to a cure fraction of 0.36 when the true value is 0.9 is not necessarily useless. In our opinion, it still provides a reasonable fit. To illustrate this, we compare the survival curves from these two fits with the true generating process (Figure 5). Despite differing cure fraction estimates, they approximate survival relatively well, even far beyond follow‐up. While they will plateau at different cure fractions as time approaches infinity, the difference may not be critical for realistic future predictions of survival. For this reason, alongside relative biases and coverages of cure fraction estimates, in mixture cure models we also report relative bias and coverage for survival estimates at t=20, which is twice the length of the follow‐up period. This provided insight into cases where the bias in cure fraction estimates was high, yet the bias in survival estimates remained low.

## 5 Application: Colon Cancer Data Set and Time‐Varying Effects

The primary focus of this analysis was to identify the prognostic factors associated with colon cancer and to showcase the application of the proposed AFT model.

We utilized a synthetic, population‐based cancer registry data set consisting of 15,564 observations (Table 7). During a maximum follow‐up of 20.5 years, 10,918 deaths occurred, including 8369 deaths due to colon cancer.

**TABLE 7:** Baseline characteristics stratified by sex.

Characteristic	Overall (n=15,564)	Female (n=9224)	Male (n=6340)
**Age, Mean (SD)**	69.13 (12.42)	70.61 (12.12)	66.98 (12.54)
**Stage at diagnosis,** n **(%)**			
Localized	6274 (40.3%)	3654 (39.6%)	2620 (41.3%)
Regional	1787 (11.5%)	1072 (11.6%)	715 (11.3%)
Distant metastasis	5147 (33.1%)	3027 (32.8%)	2120 (33.4%)
Unknown	2356 (15.1%)	1471 (15.9%)	885 (14.0%)
**Survival time (years), Mean (SD)**	3.82 (4.40)	3.89 (4.51)	3.73 (4.23)
**Status at last follow‐up,** n **(%)**			
Alive	4642 (29.8%)	2765 (30.0%)	1877 (29.6%)
Dead: Colon cancer	8369 (53.8%)	5048 (54.7%)	3321 (52.4%)
Dead: Other causes	2549 (16.4%)	1409 (15.3%)	1140 (18.0%)
Lost to follow‐up	4 (0.0%)	2 (0.0%)	2 (0.0%)

We compared the rstpm2::aft() noncure AFT model with 2 degrees of freedom to a Weibull AFT model. As expected, both approaches produced identical results in estimating the effect of sex and age on survival in patients with localized disease stage.

**FIGURE 6:**
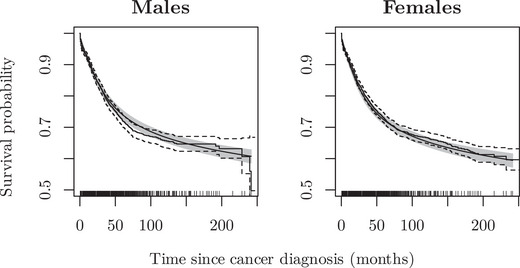
Predicted survival from a flexible parametric AFT model with 4 degrees of freedom adjusted for sex compared with the Kaplan–Meier estimates stratified by sex.

Figure 6 demonstrates that the predicted survival curves from our AFT model adjusting for sex were in good agreement with the Kaplan–Meier estimates. The survival curves for males and females tended to follow similar baselines, and increasing the splines degrees of freedom yielded a similar fit to the Kaplan–Meier estimates. This is due to the fact that the Kaplan–Meier curves were generated through separate fittings for each gender group, and therefore the shared baseline would not benefit from further adjustments. The results of the AFT model indicated that there was no statistically significant difference in survival between males and females.

**FIGURE 7:**
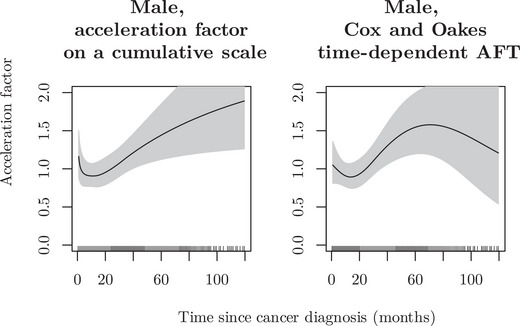
Time‐varying acceleration factor for males compared with females diagnosed with localized colon cancer at age 70 years, colon cancer.

The lack of any difference in survival between males and females may be confounded by differences in age. Incorporating natural splines for age at diagnosis (df = 4), and accounting for an integrated time‐varying effect of sex (df = 4), we found evidence for a potential time‐varying effect of sex on survival. Comparing the integrated Cox and Oakes model with a model where the cumulative effect was represented on a log‐linear time scale, the results between two models were similar: see Figure 7 for graphs of the time‐varying acceleration factors for patients aged 70 years at diagnosis with localized cancer and maximum follow‐up of 2 years. Both the model on the cumulative scale and the Cox and Oakes model suggested that males had an acceleration factor of approximately 1 in the first 20–30 months, with then an increased acceleration factor through to 60 months, after which the cumulative model continued to rise while the Cox and Oakes model had very wide confidence intervals. The minor differences between models may be attributed to their distinct model formulations.

**FIGURE 8:**
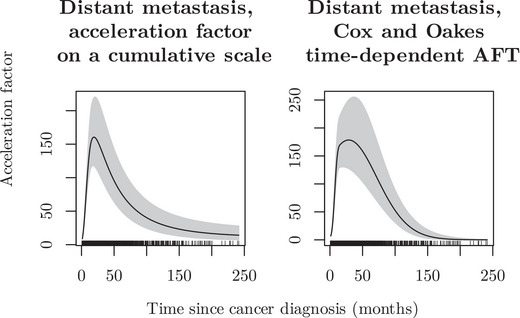
Time‐varying acceleration factor for males diagnosed with distant colon cancer (synthetic population‐based cancer registry).

We also compared patients with distant metastasis to those without, starting without any adjustment for cure. The AFT model, incorporating a time‐varying effect for the binary indicator variable representing distant metastasis, a time‐constant effect for sex, and adjusting for age, demonstrated a dramatic increase in the acceleration factor during the first year of follow‐up for the distant metastasis group, as illustrated in Figure 8.

**FIGURE 9:**
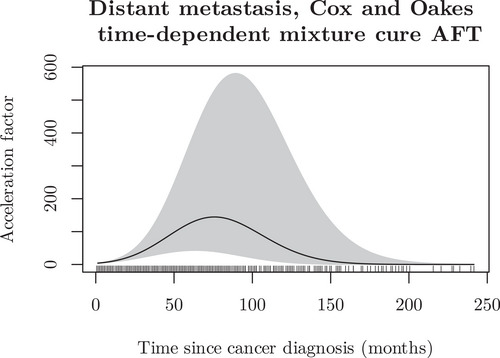
Time‐varying acceleration factor for males diagnosed with distant colon cancer accounting for cure (synthetic population‐based cancer registry).

To further explore the differences between patients with and without distant metastases, we accounted for the presence of a cure in the data set by fitting a mixture cure model with a time‐varying effect for the distant metastasis indicator, using the Cox and Oakes integrated acceleration factor formulation and adjusting for age. In addition, we allowed the cure fraction to differ between the two groups. As a result, we found that approximately 6.4% of patients with distant metastasis were cured, compared to 41.1% of patients without distant metastasis (localized or regional cancer). This aligns with the findings of Verdecchia et al. (1998), who reported a cure rate of 38.6% for colon cancer in the European population. Figure 9 illustrates the acceleration factor for 70‐year‐old males with distant metastasis compared to those without.

## 6 Discussion

In summary, we have improved and extended a flexible class of AFT models, which include cure and time‐varying acceleration factors. Moreover, we have proposed and implemented monotone natural splines that can be fitted by incorporating a linear inequality constraint as a penalty term in the likelihood. These developments and improvements, to our knowledge, have not been previously explored in the literature. They not only broaden the application of AFT models to a wider variety of real‐world data sets but also enhance their flexibility and adaptability in handling complex relationships and time‐dependent patterns in the data. This advancement paves the way for more detailed and insightful analyses. In addition, we found that AFT models not explicitly adjusted for cure performed well and were more robust to the presence of cured individuals in the data set compared to the Cox model, further strengthening the case for AFT models as a viable alternative to Cox regression.

Cure AFT models within a flexible parametric framework using mixture and nonmixture formulations adequately estimated the covariate effects, with a few exceptions in particularly challenging scenarios. The presence of cured individuals slightly increased the bias in the estimation of the covariate effect for the noncure AFT model. Moreover, despite the noncure AFT model being misspecified in this case, its performance remains comparable to that of the AFT models adjusted for cure. This observation suggests that the presence of a cure independent of covariates in the data does affect the estimation of a covariate's effect, but only to a modest extent. Nevertheless, we assumed that the cure was present at a similar level in both groups, and the situation might change if the cure fraction depends on the covariate. More importantly, the performance of the flexible AFT models varied significantly with the shape of the baseline. Some shapes of baselines were difficult to fit, even with spline degrees of freedom up to 6.

Our development of cure AFT models has faced several challenges, including low convergence rates for mixture cure models and biased estimation of cure fractions in certain scenarios for both mixture/nonmixture types. The performance of the mixture cure models in estimating the cure fraction exhibited considerable variability, with a pronounced dependence on the degrees of freedom and the characteristics of the baseline cumulative hazard function. The nonmixture cure AFT model performed very well in estimating cure fractions when the cure fraction was high, but performed poorly when the cure fraction was low.

The spline parameters played a critical role in the estimation process, highlighting the variability of obtaining accurate results with the shape of the baseline survival function. The variability in performance of the mixture cure models may be attributed to the intricacies inherent to the optimization process, where the behavior of the likelihood function becomes complex and is hindered by the existence of local extrema. For the mixture cure models, the optimization algorithm was highly sensitive to the selection of the initial values for the spline coefficients. Hanin and Huang (2014) state that short follow‐up or shared covariates may hinder the identifiability of the model. We undertook a range of additional simulations to investigate this behavior. Nonetheless, even with the assurance that every “uncured” observation was not censored and by employing a logistic model with only an intercept for the cure component, our mixture cure implementation still manifested issues in estimation. Solving this optimization issue is an open research question.

However, despite these optimization challenges, our findings suggest that mixture cure models can still provide useful survival estimates, even when the estimated cure fraction is biased. As demonstrated in our results, survival curves derived from fits with different cure fraction estimates still closely approximate the true generating process, particularly within a realistic time horizon. To further assess this, we also evaluated relative bias and coverage for survival estimates at t=20, which is twice the follow‐up period. This helped to clarify the cases where the bias in cure fraction estimates was high, yet the bias in survival estimates remained low, reinforcing the practical utility of these models in long‐term survival prediction.

Estimating the cure fraction is challenging, as it depends on whether the cure is reached within the observed data or at least not far beyond the follow‐up period. The flexible cure AFT models—both mixture and —that we developed performed well in estimating cure fractions when the cure was reached or close to being reached within the data. Surprisingly, they also succeeded in several cases where the cure was beyond follow‐up, though they failed in others, making them not entirely reliable in this regard.

Nevertheless, the most promising aspect of flexible AFT models, which we wish to emphasize, is their robustness in estimating covariate effects despite the presence of cure and regardless of whether the cure was reached within the data, provided sufficient flexibility in spline fitting. In addition, with enough flexibility, even AFT models unadjusted for cure yield good survival estimates, extending reasonably far into the future. This makes flexible AFT models a powerful tool when analyzing data without prior knowledge of whether a cure exists, whether it is reached within the data, or how far beyond follow‐up it might occur.

There are several potential extensions to the AFT models that could enhance their utility in diverse scenarios. One area of potential exploration pertains to the predictive capabilities of AFT models in terms of projecting future trends (Parsa and Van Keilegom 2023): can the AFT models really see into the future? Proportional hazards models, in contrast, conceptualize “time” in a literal sense, where times are modeled as observed. As a possible extension, one could explore AFT model selection based on the predictive performance out‐of‐sample (Tsamardinos et al. 2018).

There is an implication that AFT models might possess an innate ability to infer prospective evolutions for certain covariate patterns based on the knowledge of others. For example, if one covariate pattern is approaching cure, then that survival function may be used to predict the survival for other covariate patterns that have not yet approached cure.

Another possible extension involves the incorporation of random effects to accommodate correlated data, including clusters and recurrent events, which can help in capturing unobserved heterogeneity. In addition, the implementation of penalized splines can be achieved by either integrating random effects or employing a penalized log‐likelihood approach, aiding the smoothing of the model. Moreover, the estimation of life expectancy through the already implemented excess hazard or relative survival models represents another viable extension. AFT models can be useful in evaluating the predictive performance of survival models, such as RMST beyond the scope of observed data, as they are adept at modeling time‐to‐event distribution outside the range of observed data. One possible approach for this assessment is the utilization of strictly proper scoring rules using log scores. Furthermore, the application of AFT models in the context of competing risk and multistate models can also be explored. In addition, following a reviewer's suggestion, it would be interesting to investigate whether increasing the degrees of freedom for the baseline function affects the estimation of the time‐varying effect. These extensions collectively contribute to bolstering of AFT model's versatility in handling complex data structures and research questions.

## Conflicts of Interest

The authors declare no conflicts of interest.

## Open Research Badges

This article has earned an Open Data badge for making publicly available the digitally‐shareable data necessary to reproduce the reported results. The data is available in the Supporting Information section.

This article has earned an open data badge “**Reproducible Research**” for making publicly available the code necessary to reproduce the reported results. “The results reported in this article could fully be reproduced.”

## Supporting information

Supporting Information

## Figures and Tables

**FIGURE A1:**
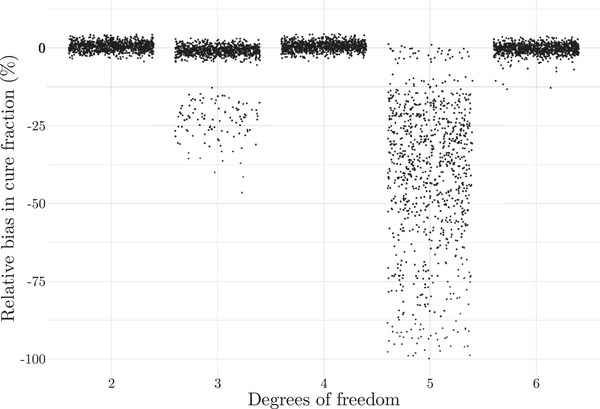
Relative bias in cure fraction (%) for mixture cure AFT model, baseline type 1, cure fraction 0.5.

**FIGURE A2:**
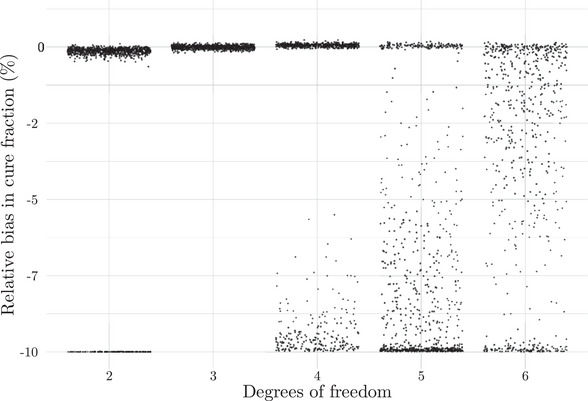
Relative bias in cure fraction (%) for mixture cure AFT model, baseline type 3, cure fraction 0.9.

**FIGURE A3:**
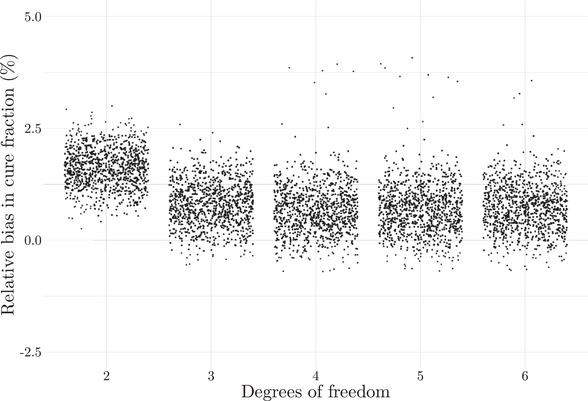
Relative bias in cure fraction (%) for nonmixture cure AFT model, baseline type 3, cure fraction 0.9.

**FIGURE A4:**
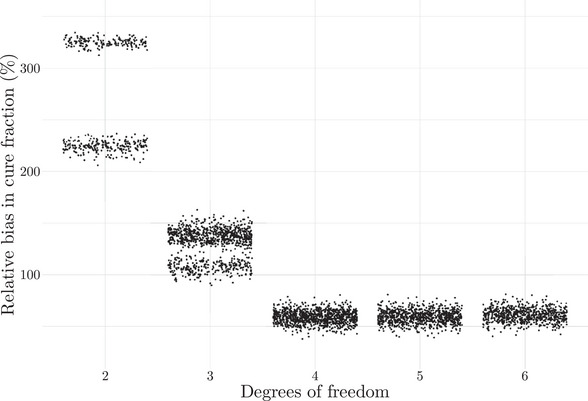
Relative bias in cure fraction (%) for nonmixture cure AFT model, baseline type 3, cure fraction 0.1.

**FIGURE A5:**
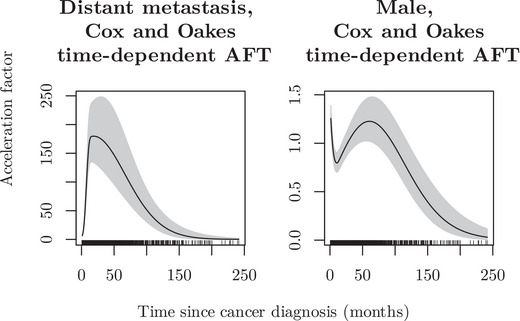
Time‐varying acceleration factor for patients diagnosed with distant colon cancer to patients without distant metastasis (synthetic population‐based cancer registry).

## Data Availability

The data that support the findings of this study are available in the supplementary material of this article.

## Appendix A Baseline Representation With an Integration for the Acceleration Factor

Under the baseline representation for $H_{0}$ and an integrated acceleration factor, and $\tilde{t}=\int_{0}^{t}\exp\left(-\beta^{T}x\left(u\right)\right)du$. Then, we can then express the survival, cumulative hazard, and hazard functions as

$$\begin{array}{cc} S(t|\bar{X}\left(t\right),\beta ,\gamma ) & =\exp\left(-\exp\left(B{\left(\log(\tilde{t})\right)}^{T}\gamma \right)\right)\\ H(t|\bar{X}\left(t\right),\beta ,\gamma ) & =\exp\left(B{\left(\log(\tilde{t})\right)}^{T}\gamma \right)\\ h(t|\bar{X}\left(t\right),\beta ,\gamma ) & =\exp\left(B{\left(\log(\tilde{t})\right)}^{T}\gamma \right)\left(B^{\prime }{\left(\log(\tilde{t})\right)}^{T}\gamma \right)\exp\left(-\beta^{T}x\left(t\right)\right)/\tilde{t}. \end{array}$$

### A.1 Gradients With Respect to β

$$\begin{array}{cc} \frac{\partial H(t|\bar{X}\left(t\right),\beta ,\gamma )}{\partial \beta_{j}}=\; & -H\left(t|\bar{X}\left(t\right),\beta ,\gamma \right)\left(B^{\prime }{\left(\log\left(\tilde{t}\right)\right)}^{T}\gamma \right)\\ & \times \int_{0}^{t}x_{j}\left(u\right)\exp\left(-\beta^{T}x\left(u\right)\right)du\;/\;\tilde{t}\\ \frac{\partial \log(h\left(t|\bar{X}\left(t\right),\beta ,\gamma \right))}{\partial \beta_{j}}=\; & -\frac{\left(B^{\prime \prime }{\left(\log\left(\tilde{t}\right)\right)}^{T}\gamma \right)\int_{0}^{t}x_{j}\left(u\right)\exp\left(-\beta^{T}x{\left(u\right)}^{T}\right)du}{\left(B^{\prime }{\left(\log\left(\tilde{t}\right)\right)}^{T}\gamma \right)\tilde{t}}\\ & +\frac{\int_{0}^{t}x_{j}\left(u\right)\exp\left(-\beta^{T}x{\left(u\right)}^{T}\right)du}{\tilde{t}}-x_{j}\left(t\right)\\ & -\left(B^{\prime }{\left(\log\left(\tilde{t}\right)\right)}^{T}\gamma \right)\left(\int_{0}^{t}x_{j}\left(u\right)\exp\left(-\beta^{T}x{\left(u\right)}^{T}\right)du\right)/\tilde{t}. \end{array}$$

### A.2 Gradients With Respect to γ

$$\begin{array}{cc} \frac{\partial H(t|\bar{X}\left(t\right),\beta ,\gamma )}{\partial \gamma_{j}} & =H\left(t|\bar{X}\left(t\right),\beta ,\gamma \right)B_{j}\left(\log\left(\tilde{t}\right)\right)\\ \frac{\partial \log(h\left(t|\bar{X}\left(t\right),\beta ,\gamma \right))}{\partial \gamma_{j}} & =B_{j}\left(\log\left(\tilde{t}\right)\right)+\frac{B_{j}^{\prime }\left(\log\left(\tilde{t}\right)\right)}{B^{\prime }{\left(\log\left(\tilde{t}\right)\right)}^{T}\gamma }. \end{array}$$

## Appendix B Natural Splines for Nonmixture Cure Models

There are at least three different approaches for natural splines: (i) using a truncated power basis; (ii) using a B‐splines basis with a null‐space projection for the boundary constraints using a QR decomposition; and (iii) using a B‐splines basis with an algebraic solution to the boundary constraints (Wang and Yan 2021). Extensions to nonmixture cure models with zero slope at the right boundary knot have been provided for approaches (i) and (ii) (Andersson et al. 2013; Jakobsen et al. 2020).

For an algebraic solution to using B‐splines for nonmixture cure models, with constraints for the second derivatives being zero at the left and right boundary knots, and the first derivative being zero at the right boundary, we can extend the approach followed by Wang and Yan (2021). Assuming cubic B‐splines with four repeated values for each boundary knot, we have a set of knots $t_{1}=t_{2}=t_{3}=t_{4}<t_{5}<\cdots <t_{k}=t_{k+1}=t_{k+2}=t_{k+3}$. For a B‐spline design matrix B, we have a null‐space projection matrix H, such that the transformed design matrix is BH. For no internal knots, we have $H={\left[\begin{array}{cccc} 1, & 1, & 1, & 1 \end{array}\right]}^{T}$, and BH is a vector of ones. We define $C=\left(t_{k-1}-t_{k-2}\right)/\left(t_{k}+t_{k-1}-2t_{k-2}\right)$. For one internal knot, we have

$$\begin{array}{c} H^{T}=\left[\begin{array}{ccccc} 1 & 1 & 1 & 1 & 1\\ 0 & C & 1 & 1 & 1 \end{array}\right]. \end{array}$$

For two internal knots, we have

$$\begin{array}{c} H^{T}=\left[\begin{array}{cccccc} 1 & 1 & 1 & 0 & 0 & 0\\ 0 & C & 1 & 1 & 1 & 1\\ 0 & 0 & 0 & 1 & 1 & 1 \end{array}\right]. \end{array}$$

Finally, for more than two internal knots, we have that

$$\begin{array}{c} H^{T}=\left[\begin{array}{ccccccc} 1 & 1 & 1 & 0^{T} & 0 & 0 & 0\\ 0 & C & 1 & 1^{T} & 1 & 1 & 1\\ 0 & 0 & 0 & I_{k-7} & 0 & 0 & 0\\ 0 & 0 & 0 & 0^{T} & 1 & 1 & 1 \end{array}\right], \end{array}$$

where $I_{k-7}$ is an identity matrix with k−7 rows and k−7 columns.

As a further extension, we can assume a constant hazard after the last knot, which is consistent with the log cumulative hazard function as a function of log time having a slope of 1 (Andersson et al. 2013). This can be modeled by setting the first derivative at the right boundary knot as being 1. If the coefficients for the projected splines are represented by β, then the linear predictor for the splines is B(Hβ+δ) for some offset term δ. The H matrices are as per the cure models. The offset terms can also be solved algebraically. Using the same set of knots defined earlier in this section, let $C_{1}=\left(t_{k}-t_{k-1}\right)/3$ and $C_{2}=\left(t_{k}-t_{k-2}\right)/3$. Then the offset terms are provided in Table A1.

**TABLE A1:** Offset terms for B‐spline projections with the first derivative at the right boundary knot equal to one.

Number of internal knots	Offset term δ
Zero	${\left[\begin{array}{cccc} 0, & C_{1}, & 2C_{1}, & 3C_{1} \end{array}\right]}^{T}$
One	${\left[\begin{array}{ccccc} C_{1}-C_{2}, & 0, & -C_{2}, & 0, & 3C_{1} \end{array}\right]}^{T}$
Even number	${\left[\begin{array}{cccc} 0_{k-4}^{T}, & -C_{2}, & 0, & C_{1} \end{array}\right]}^{T}$
Odd number of internal knots with at least three knots	${\left[\begin{array}{cccc} 0_{k-4}^{T}, & -C_{1}-C_{2}, & -C_{1}, & 0 \end{array}\right]}^{T}$

## Appendix C Gradients for the Mixture Cure Model

Following the notation from Section 2.6:

$$\begin{array}{cc} & \nabla H(t|\bar{X}\left(t\right),\beta ,\gamma ,\theta )\\ & \;=\left(\left(1-\pi \left(X\left(0\right)|\theta \right)\right)\exp\left(-H_{u}\left(t|\bar{X}\left(t\right),\beta ,\gamma \right)\right)\nabla H_{u}\left(t|\bar{X}\left(t\right),\beta ,\gamma \right)\right.\\ & \;\left.-\nabla \pi \left(X\left(0\right)|\theta \right)(1-\exp\left(-H_{u}\left(t|\bar{X}\left(t\right),\beta ,\gamma \right)\right))\right)/S\left(t|\bar{X}\left(t\right),\beta ,\gamma ,\theta \right)\\ & \nabla \log(h\left(t|\bar{X}\left(t\right),\beta ,\gamma ,\theta \right))\\ & \;=-\frac{\nabla \pi (X(0)|\theta )}{1-\pi (X(0)|\theta )}+\nabla \log\left(h_{u}\left(t|\bar{X}\left(t\right),\beta ,\gamma \right)\right)\\ & \;-\nabla H_{u}\left(t|\bar{X}\left(t\right),\beta ,\gamma \right)+\nabla H\left(t|\bar{X}\left(t\right),\beta ,\gamma ,\theta \right). \end{array}$$

If π(X(0)|θ) follows a logistic function, then

$$\begin{array}{c} \frac{\partial }{\partial \theta_{j}}\pi \left(X\left(0\right)|\theta \right)=X_{j}\left(0\right)\pi \left(X\left(0\right)|\theta \right)\left(1-\pi \left(X\left(0\right)|\theta \right)\right). \end{array}$$

## Appendix D Relative Survival

As a further extension, we consider a relative survival or *excess hazard* model, such that

$$\begin{array}{cc} h(t|\bar{X}\left(t\right),\beta ,\gamma )=\; & h_{0}\left(\int_{0}^{t}\exp\left(-\beta^{T}x\left(u\right)\right)du)|\;\gamma \right)\\ & \times \exp\left(-\beta^{T}x\left(t\right)\right)+h^{*}\left(t|\bar{X}\left(t\right)\right), \end{array}$$

where $h^{*}\left(t|\bar{X}\left(t\right)\right)$ is the background expected hazard based on life tables or another external data source, and $h_{0}\left(\int_{0}^{t}\exp\left(-\beta^{T}x\left(u\right)\right)du)|\;\gamma \right)\exp\left(-\beta^{T}x\left(t\right)\right)$ is the excess hazard. The most common application is to model for all‐cause mortality for a particular patient group and adjust for the background mortality for that group.

## Appendix E Simulation Results

### E.1 Does Noncure Accelerated Failure Time (AFT) Perform Worse in the Presence of Cure?

Performance in estimating the covariate effects of the noncure AFT model slightly deteriorates when cured individuals are present in the data set, as evidenced by increasing bias in the estimation of β (log acceleration factor) in Table A2. Increasing the degrees of freedom of splines did not consistently reduce bias. Similar results were consistent across all baseline types. Nevertheless, the performance of the noncure AFT model in estimating the covariate effect was comparable to that of the AFT models adjusted for cure.

**TABLE A2:** Relative bias (%) in the estimation of log acceleration factor for noncure AFT model for baseline type 4 (standard Weibull).

	**Cure fraction**			
**df**	0	0.1	0.5	0.9
2	1.9	2.5	7.7	12.9
3	2.0	2.2	3.6	9.0
4	2.1	2.2	3.2	8.4
5	2.1	2.1	3.3	8.6
6	2.1	2.2	3.4	8.5

### E.2 Complex Baseline Shapes

The bias in estimating the covariate effect was influenced by the shape of the baseline hazard function. For example, the bias was considerably worse when the baseline followed a complex shape (Table A3). In this scenario, increasing the degrees of freedom of splines did not consistently mitigate the issue.

**TABLE A3:** Relative bias (%) in the estimation of log acceleration factor for noncure AFT model for baseline type 2.

	**Cure fraction**			
**df**	0	0.1	0.5	0.9
2	18.7	9.4	17.5	41.0
3	3.6	8.0	34.7	49.7
4	33.1	15.7	36.2	28.2
5	24.8	31.4	39.1	36.5
6	15.6	17.5	17.5	15.8

### E.3 Estimation of Cure Fractions

The mixture cure model generally performed poorly in estimating the cure fractions across most scenarios. However, there were a few instances where it achieved good accuracy, making its performance inconsistent, as observed in Figures A1 and A2.

On the other hand, the nonmixture cure model showed excellent performance for higher cure fractions of 0.5 and 0.9 (Figure A3) but was much less effective in estimating lower cure fractions of 0.1 (Figure A4) and 0.

## Appendix F Colon Cancer Data Set With Time‐Varying Effects

The Implemented Methodology is capable of accommodating numerous time‐varying effects concurrently. In this analysis, we specifically model both sex and distant stage as having time‐varying effects, acceleration factors for distant metastases state and sex are presented in Figure A5.
