# Supplementary material for: Flexible Parametric Accelerated Failure Time Models With Cure
Source: Biom J. 2025 Sep 10;67(5):e70074. doi: 10.1002/bimj.70074 (PMC12423370; doi:10.1002/bimj.70074)
Supplement: Supplementary file 1 — Supporting Information [file BIMJ-67-e70074-s001.zip › code_and_data/ReproducibleResearch.pdf]

Flexible parametric accelerated failure time models with cure

Birzhan Akynkozhayev Benjamin Christoffersen Xingrong Liu
Keith Humphreys Mark Clements

2025-06-19

Contents

R packages **3**

Figure 1: Baseline hazard types for the uncured **3**

Figure 2: Survival curves under all four baseline scenarios with a cure fraction of 0.5 **6**

Simulations **8**

Loading Intermediate Results of Simulations . . . 9

Cleaning, Formatting, and Preparing Data from Intermediate Results for Time Constant Effects Simulations . . . 9

Convergence rates of model fits . . . 10

Tables 1-4 **11**

Table 5: Comparison of Cox PH and non-cure AFT (df = 2 and 6) in estimating log hazard ratios (Cox PH) and log **13**

Table 6: Simulation results for time-varying acceleration factors **19**

Figure 3: Non-cure AFT fit for Scenario 1 with a cure fraction of 0.9: Log-cumulative hazard on the log-time scale, comparing estimated and true generating processes **21**

Figure 4: Non-cure AFT fit for Scenario 1 with a cure fraction of 0.9: Survival curves comparing estimated and true generating processes **22**

Figure 5: Survival curves for the same dataset from two fits of the model using different initial values, compared with the true generating process **24**

Table 7: Baseline Characteristics Stratified by Sex **26**

Figure 6: Predicted survival from a flexible parametric AFT model with four degrees of freedom adjusted for sex compared with the Kaplan-Meier estimates stratified by sex **27**

Figure 7: Time-varying acceleration factor for males compared with females diagnosed with localised colon cancer at age 70 years, colon cancer **28**

Figure 8: Time-varying acceleration factor for males diagnosed with distant colon cancer **29**

Figure 9: Time-varying acceleration factor for males diagnosed with distant colon cancer accounting for cure **31**

Table 9 (Appendix): Relative bias in the estimation of log acceleration factor for non-cure AFT model for baseline type 4 **31**

Table 10 (Appendix): Relative bias (%) in the estimation of log acceleration factor for non-cure AFT model for baseline type 2 **33**

Figure 10 (Appendix): Relative bias in cure fraction (%) for mixture cure AFT model, baseline type 1, cure fraction 0.5 **34**

Figure 11 (Appendix): Relative bias in cure fraction (%) for mixture cure AFT model, baseline type 3, cure fraction 0.9 **35**

Figure 12 (Appendix):Relative bias in cure fraction (%) for non-mixture cure AFT model, baseline type 3, cure fraction 0.9 **36**

Figure 13 (Appendix): Relative bias in cure fraction (%) for non-mixture cure AFT model, baseline type 3, cure fraction 0.1 **37**

Figure 14: Time-varying acceleration factor for patients diagnosed with distant colon cancer to patients without distant metastasis **38**

Session infos **39**

Session info for this RMD 39

Session info for Time Varying Effects Simulations 40

Session info for Time Constant Effects Simulations 41

R packages

See Session info for this RMD document

```r
  library(rstpm2)
  library(ggplot2)
  library(hrbrthemes)
  library(extrafont)
  library(ggpubr)
  library(dplyr)
  library(officer)
  library(flextable)
  library(kableExtra)
  library(knitr)
  library(tidyr)
  library(scales)
  library(tableone)
  library(viridis)
  library(rio)
  library(flexlsx)

if (FALSE) { # change to TRUE to install and load missing packages
  pkgs <- c(
  "rstpm2",   "ggplot2",  "hrbrthemes", "extrafont", "ggpubr",
  "dplyr",    "officer",  "flextable",   "kableExtra","knitr",
  "tidyr",    "scales",   "tableone",    "viridis", "rio", "flexlsx")
  to_install <- pkgs[!pkgs %in% installed.packages()[, "Package"]]
  if (length(to_install)) install.packages(to_install)
  lapply(pkgs, library, character.only = TRUE)
}
```

Figure 1: Baseline hazard types for the uncured

```r
# Distributional parameters (shapes, scales, mixing parameters) for a mixture of
# Weibull distributions for four different scenarios

# Transformation of scale parameter from Crowther Lambert to R Weibul scale
scale_tr = \(lambda, gamma) c(lambda[1]^(-1 / gamma[1]), lambda[2]^(-1 /
                                                                   gamma[2]))
# args: sce (scenario 1-4); returns: list with mixing_par, shape, and scale
dist <- \(sce) {
  switch(sce,
    list(
      mixing_par = 0.8,
      shape = c(3, 1.6),
      scale = scale_tr(c(0.1, 0.1), c(3, 1.6)) # 2.154435 4.216965
    ),
    list(
      mixing_par = 0.5,
      shape = c(1.5, 0.5),
      scale = scale_tr(c(1, 1), c(1.5, 0.5)) # 1 1
```

```r
    ),
    list(
      mixing_par = 0.26,
      shape = c(3, 0.7),
      scale = scale_tr(c(0.02, 0.5), c(3, 0.7)) # 3.684031 2.691800
    ),
    list( # This is a simple Weibull
      mixing_par = 0.5,
      shape = c(1.2, 1.2),
      scale = scale_tr(c(0.1, 0.1), c(1.2, 1.2)) # 6.812921 6.812921
    )
  )
}

# Baseline hazards for the mixutre of Weibulls
# args: t (time); sce (scenario 1-4); returns: hazard at time t
hazard <- \(t, sce) {
  distr <- dist(sce)
  (
    distr$mixing_par * dweibull(t, distr$shape[1], distr$scale[1]) +
      (1 - distr$mixing_par) * dweibull(t, distr$shape[2], distr$scale[2])
  ) / (
    distr$mixing_par * (1-pweibull(t, distr$shape[1], distr$scale[1])) +
      (1 - distr$mixing_par) * (1-pweibull(t, distr$shape[2], distr$scale[2]))
  )
}

# Plotting the hazards for the uncured

# theme setting for ggplot, will be reused in most plots
base_theme <- theme_ipsum() +
  theme(
    panel.border    = element_rect(fill = NA),
    legend.position = "none",
    axis.text.x     = element_text(
      size = 12,
      color = "black",
      family = "LM Roman 10"
    ),
    axis.text.y     = element_text(
      size = 12,
      color = "black",
      family = "LM Roman 10"
    ),
    axis.title.x    = element_text(
      size = 24,
      color = "black",
      family = "LM Roman 10",
      hjust = 0.5
    ),
    axis.title.y    = element_text(
      size = 24,
      color = "black",
```

```
      family = "LM Roman 10",
      hjust = 0.5
    )
  )

# hazard plot fot a single scenario
# args: sce (scenario 1-4); returns: ggplot of the hazard function
make_hazard_plot <- \(sce) {
  ggplot() +
    xlim(0, 5) + ylim(0, 1.2) +
    geom_function(fun = hazard, args = list(sce = sce)) +
    base_theme +
    labs(x = "time (years)", y = "hazard")
}

# generate hazard plots for sce = 1:4
plots <- lapply(1:4, make_hazard_plot)

# strip individual x/y labels (we'll add shared ones later)
plots_nolabs <- lapply(plots, \(p) p + rremove("xlab") + rremove("ylab"))

# arrange them in a 2X2 grid
figure_1 <- do.call(ggarrange, c(
  plots_nolabs,
  list(
    labels     = paste("Type", 1:4),
    font.label = list(size = 14, color = "black", family = "LM Roman 10"),
    ncol       = 2, nrow = 2
  )
))

# add the shared axis titles
figure_1 <- annotate_figure(
  figure_1,
  left   = text_grob(
    "Hazard",
    family = "LM Roman 10",
    rot = 90,
    vjust = 1,
    size = 18
  ),
  bottom = text_grob("Time", family = "LM Roman 10", size = 18)
)


if(interactive()) {print(figure_1)}
# figures look better in saved versions, please see ./results/figures/

# save
ggsave(
  "./results/figures/Figure_1.pdf",
  plot   = figure_1,
  device = cairo_pdf,
```

```
  width  = 300,
  height = 200,
  units  = "mm",
  create.dir = TRUE
)
```

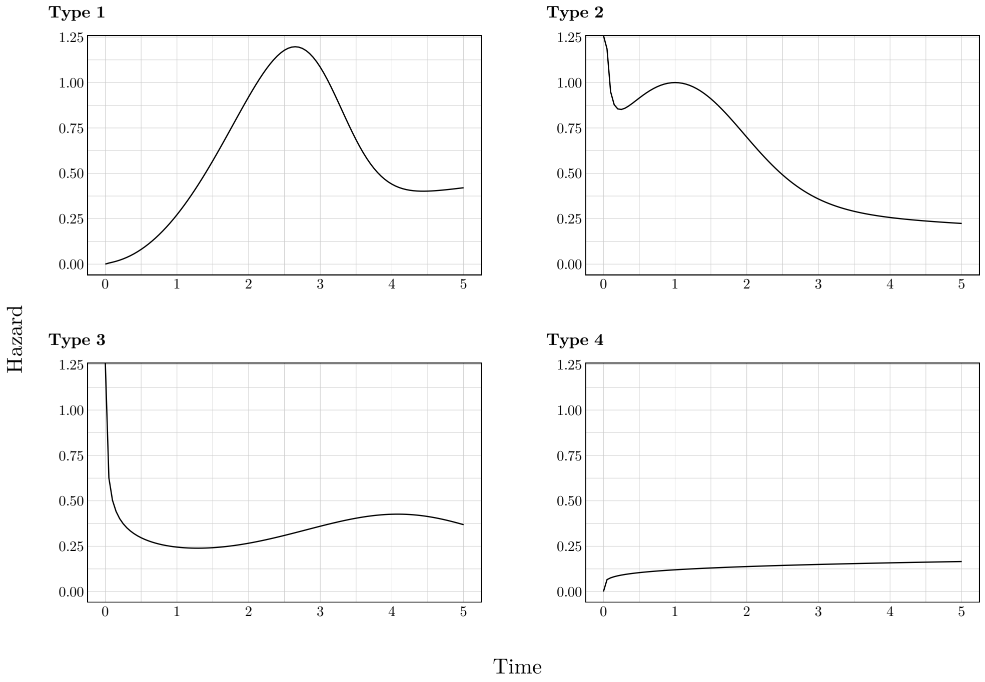

Figure 1: Baseline hazard types for the uncured

Figure 2: Survival curves under all four baseline scenarios with a cure fraction of 0.5

```
# Survival for the mixutre of Weibulls
# args: sce (scenario 1-4), t (time), X (covariate value), cf (cure fraction);
# returns: survival probability
theoretical_survival <- function(sce, t, X = 0, cf = 0.5) {
  d      <- dist(sce)
  mix    <- d$mixing_par
  sh     <- d$shape
  sc     <- d$scale
  t_star <- t * exp(-X) # beta = 1, aft formulation
  S1     <- pweibull(t_star, shape = sh[1], scale = sc[1], lower.tail = FALSE)
  S2     <- pweibull(t_star, shape = sh[2], scale = sc[2], lower.tail = FALSE)
  cf + (1 - cf) * (mix * S1 + (1 - mix) * S2)
}

t_seq <- seq(0, 20, length.out = 300)
```

```r
# Plot surival for each scenario
plots2 <- lapply(1:4, \(sce) {
  df <- do.call(rbind, lapply(0:1, \(x) {
    data.frame(
      time = t_seq,
      surv = vapply(t_seq,
                    \(tt) theoretical_survival(sce, tt, X = x, cf = 0.5),
                    numeric(1)),
      X = factor(paste0("X=", x))
    )
  }))
  ggplot(df, aes(time, surv, color = X)) +
    geom_line(size = 0.5) +
    geom_vline(xintercept = 10, linetype = 2, size = 0.5) +
    scale_x_continuous(limits = c(0, 20)) +
    scale_y_continuous(limits = c(0.48, 1)) +
    scale_color_manual("", values = c("X=0" = "#0072B2", "X=1" = "#D55E00")) +
    labs(x = NULL, y = NULL) +
    base_theme +
    theme(
      axis.title      = element_text(size = 16),
      legend.position = "right",
      legend.text     = element_text(size = 12, color = "black",
                                     family = "LM Roman 10")
    )
})

# strip x/y labels and arrange in 2X2
plots2_clean <- lapply(plots2, \(p)
  p + rremove("xlab") + rremove("ylab"))
fig2 <- do.call(ggarrange, c(
  plots2_clean,
  list(
    ncol          = 2,
    nrow = 2,
    common.legend = TRUE,
    legend = "right",
    labels        = paste("Type", 1:4),
    font.label    = list(size = 14, family = "LM Roman 10")
  )
))

# add one big axis labels
figure_2 <- annotate_figure(
  fig2,
  left   = text_grob(
    "Survival Probability",
    rot = 90,
    size = 16,
    family = "LM Roman 10"
  ),
  bottom = text_grob("Time", size = 16, family = "LM Roman 10")
)
```

```r
if(interactive()){print(figure_2)}
# figures look better in saved versions, please see ./results/figures/
# save
ggsave(
  "./results/figures/Figure_2.pdf",
  plot       = figure_2,
  device     = cairo_pdf,
  width      = 300,
  height     = 200,
  units      = "mm",
  create.dir = TRUE
)
```

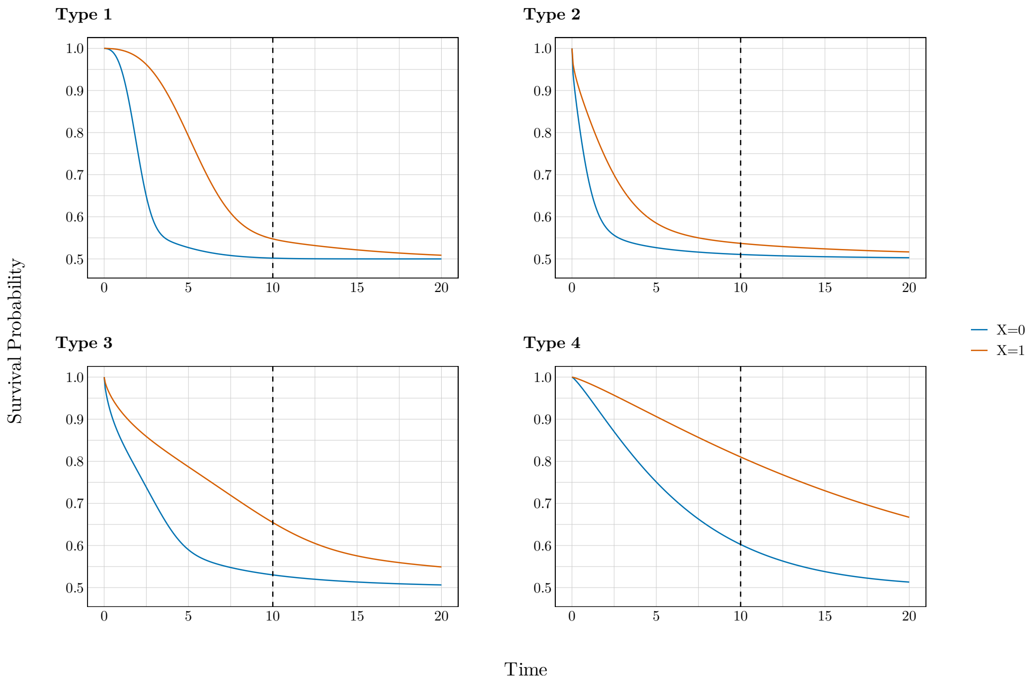

Figure 2: Survival curves under all four baseline scenarios with a cure fraction of 0.5. The dashed line marks the end of follow-up at 10

Simulations

Run the following tow code files to generate the datasets, fit the models, and collect various summary statistics from each fitted model. With the current seeds they will reproduce exactly results in the manuscript. All simulations for time-constant effects were run on a node with two AMD EPYC 9274F 24-core processors and require at least 64 GB of RAM, and are completed in under four hours. `sim_fits()` function accepts a `cores` argument.

Time constant effects simulations

simulations_time_constant_effects.R

See Session info

Time varying effects simulations

simulations_time_varying_effects.R

See Session info

Since the simulations are computationally intensive, we provide pre-computed RData files, you can load the intermediate results directly.

Loading Intermediate Results of Simulations

```r
load("./simulations/Intermediate_Results_TCE.RData")
load("./simulations/Intermediate_Results_TVE.RData")
```

Cleaning, Formatting, and Preparing Data from Intermediate Results for Time Constant Effects Simulations

```r
# merge results, fix column types, calculate and add absolute value columns,
# add convergence flag based on conditions listed below
clean_results <-
  list(results_cf_0, results_cf_01, results_cf_05, results_cf_09) |>
  bind_rows() |>
  mutate(
    # numeric
    across(c(
      sce, sim_num, cure_frac, df, cens_prop,
      bias_beta, bias_relative_beta,
      bias_cure_frac, bias_relative_cure_frac,
      bias_cf_f, bias_cf_f_rel,
      AIC, BIC, sum_gradients, D_KL0, D_KL1
    ), as.numeric),
    # absolute values
    across(c(
      bias_beta, bias_relative_beta,
      bias_cure_frac, bias_relative_cure_frac,
      bias_cf_f, bias_cf_f_rel
    ), ~ abs(.), .names = "{.col}_abs"),
    # logical
    across(c(
      coverage_beta, coverage_cure_frac, coverage_cure_frac_f,
      NA_vcov, neg_diag_vcov
    ), as.logical),
    # convergence criteria:
    # no NA entries in the variance covariance matrix
    # no negative diagonal entries in vcov
    # sum_gradients < 3 (sum of absolute values of gradients,
    # from simulations code)
    # no errors
    # all coxph fits converged
    convergence = (
      !NA_vcov & !neg_diag_vcov & sum_gradients < 3 &
```

```
      (is.na(err_msg) | err_msg == "")
    ) | model == "coxph"
  )
```

Convergence rates of model fits

```
# Convergence by model type
conv_by_model <- clean_results |>
  group_by(model) |>
  summarise(
    total            = n(),
    converged        = sum(convergence, na.rm = TRUE),
    convergence_rate = converged / total
  ) |>
  mutate(across(where(is.numeric), ~ signif(.x, 3)))

# Convergence by df for mixture_cure
conv_by_df <- clean_results |>
  filter(model == "mixture_cure") |>
  group_by(df) |>
  summarise(
    total            = n(),
    converged        = sum(convergence, na.rm = TRUE),
    convergence_rate = converged / total
  ) |>
  mutate(across(where(is.numeric), ~ signif(.x, 3)))

cat("**Convergence by model type**\n\n")
```

```
## **Convergence by model type**
```

```
print(kable(conv_by_model,  booktabs = TRUE))
```

```
##
##
## |model            | total| converged| convergence_rate|
## |:----------------|-----:|---------:|----------------:|
## |coxph            |  4000|      4000|            1.000|
## |mixture_cure     | 79900|     50300|            0.629|
## |non_cure_aft     | 80000|     79300|            0.992|
## |non_mixture_cure | 80000|     71500|            0.894|
```

```
cat("\n\n**Convergence by degrees of freedom for mixture_cure**\n\n")
```

```
##
##
## **Convergence by degrees of freedom for mixture_cure**
```

```
print(kable(conv_by_df,    booktabs = TRUE))
```

```
##
##
## | df| total| converged| convergence_rate|
## |--:|-----:|---------:|----------------:|
## |  2| 16000|     15800|            0.990|
## |  3| 15900|      9950|            0.628|
## |  4| 16000|      8420|            0.526|
## |  5| 16000|      8200|            0.513|
## |  6| 16000|      7860|            0.491|
```

Tables 1-4

```
# table border settings
std_border <- fp_border(color = "black")
# helper function to save flextables

save_flextable_xlsx <- \(ft, dir, sheet = "Sheet1", cell = "B2") {
  wb <- openxlsx2::wb_workbook()$add_worksheet(sheet)
  wb <- wb_add_flextable(wb, sheet, ft, dims = cell)
  wb$save(dir)
  invisible()
}
tabulator_to_df <- function(tab) {
  stopifnot("body" %in% names(tab))

  df <- as.data.frame(
    lapply(tab$body, function(col) {
      # Convert paragraphs to text
      sapply(col, as.character)
    }),
    stringsAsFactors = FALSE
  )

  names(df) <- names(tab$columns)
  df
}
# one flextable per scenario
# args: sce_num (scenario index);
# returns: tibble of summary metrics for that scenario
make_table_for_sce <- \(sce_num) {
  conv_summary <- clean_results |>
    filter(sce == sce_num, model != "coxph") |>
    group_by(cure_frac, model, df) |>
    summarise(
      total                  = n(),
      converged              = sum(convergence,
                                   na.rm = TRUE),
      conv_rate              = 100 * converged / total,
```

```
    bias_rel_beta_abs_med      = median(bias_relative_beta_abs[convergence],
                                        na.rm = TRUE),
    coverage_beta_avg          = mean(coverage_beta[convergence],
                                      na.rm = TRUE)*100,
    bias_rel_cure_frac_abs_med = median(
      bias_relative_cure_frac_abs[convergence],
                                        na.rm = TRUE),
    bias_cure_frac_avg         = median(bias_cure_frac_abs[convergence],
                                        na.rm = TRUE),
    coverage_cure_frac_avg     = mean(coverage_cure_frac[convergence],
                                      na.rm = TRUE)*100,
    bias_cf_f_rel_abs_med      = median(bias_cf_f_rel_abs[convergence],
                                        na.rm = TRUE),
    coverage_cure_frac_f_avg   = mean(coverage_cure_frac_f[convergence],
                                      na.rm = TRUE) * 100,
    AIC_med                    = median(AIC[convergence],
                                        na.rm = TRUE) / 1000,
    BIC_med                    = median(BIC[convergence],
                                        na.rm = TRUE) / 1000,
    D_KL0_med                  = median(D_KL0[convergence],
                                        na.rm = TRUE),
    D_KL1_med                  = median(D_KL1[convergence],
                                        na.rm = TRUE),
    .groups = "drop"
  )

  tab <- tabulator(
    x       = conv_summary,
    rows    = c("cure_frac", "df"),
    columns = "model",
    `Total runs`                = as_paragraph(total),
    `% converged`               = as_paragraph(conv_rate),
    `% bias beta`               = as_paragraph(bias_rel_beta_abs_med),
    `% coverage beta`           = as_paragraph(coverage_beta_avg),
    `bias_cure_frac`            = as_paragraph(bias_cure_frac_avg),
    `% bias cure fraction`      = as_paragraph(bias_rel_cure_frac_abs_med),
    `% coverage cure fraction`  = as_paragraph(coverage_cure_frac_avg),
    `% bias cure fraction f`    = as_paragraph(bias_cf_f_rel_abs_med),
    `% coverage cure fraction f` = as_paragraph(coverage_cure_frac_f_avg),
    `AIC (x1000)`               = as_paragraph(AIC_med),
    `BIC (x1000)`               = as_paragraph(BIC_med)
  )

   ft <- as_flextable(tab) |>
    vline(border = fp_border(color = "black")) |>
    theme_booktabs()

# save
subdir <- paste0("./results/tables/table_", sce_num)
filename <- paste0("table_", sce_num)
xlsx_file <- file.path(subdir, paste0(filename, ".xlsx"))
csv_file  <- file.path(subdir, paste0(filename, ".csv"))
dir.create(subdir, recursive = TRUE)
```

```r
  # as xlsx through "flexlsx" package
  save_flextable_xlsx(ft, dir = xlsx_file)
  # convert to .csv via "rio" package
  convert(xlsx_file, csv_file,
          in_opts = list(.name_repair = "minimal"))
  ft
}

tables <- lapply(1:4, make_table_for_sce)

# only print in an interactive session
if (interactive()) {
  for (i in seq_along(tables)) {
    cat("\n\n### Scenario", i, "\n\n")
    print(tables[[i]])
  }
}
```

These summary tables are too wide to include legibly in the PDF in this `print(tables[[i]])` format. To view them at full size, please run the above chunk interactively in R. We have translated them into LaTeX `sidewaystable` and included them below.

Table 5: Comparison of Cox PH and non-cure AFT (df = 2 and 6) in estimating log hazard ratios (Cox PH) and log

acceleration factors (AFT) for baseline type 4 (standard Weibull)

```r
# calculate the summary stats

# args: df (data frame with convergence, bias_relative_beta_abs, coverage_beta,
# AIC, BIC); returns: tibble with the summary stats
compute_metrics <- \(df) {
  df |>
    summarise(
      `% Bias beta`   = signif(median(bias_relative_beta_abs[convergence],
                                      na.rm = TRUE), 3),
      `% Cov. beta`   = signif(100 * mean(coverage_beta[convergence],
                                          na.rm = TRUE), 3),
      AIC             = signif(median(AIC[convergence],
                                      na.rm = TRUE) / 1000, 3),
      BIC             = signif(median(BIC[convergence],
                                      na.rm = TRUE) / 1000, 3),
      `% Converged`   = signif(100 * sum(convergence,
                                         na.rm = TRUE) / n(), 3)
    )
}

# compute for each model type; we are comparing only coxph & non_cure_aft
df_cox  <- clean_results |>
  filter(sce == 4, model == "coxph") |>
  group_by(cure_frac) |>
```

cure fraction	df	non-cure AFT					mixture cure AFT									non-mixture cure AFT						
		% Bias beta	% Cov. beta	AIC	BIC	% Con.	% Bias beta	% Cov. beta	% Bias cure frac.	% Cov. cure frac.	% Bias surv t=20	% Cov. surv t=20	AIC	BIC	% Con.	% Bias beta	% Cov. beta	% Bias cure frac.	% Cov. cure frac.	AIC	BIC	% Con.
0.0	2	8.1	0.0	27.2	27.2	99.6	6.8	0.0	0.9	0.0	0.9	0.0	27.0	27.0	99.9	0.9	92.5	1.3	0.0	26.3	26.3	99.9
	3	1.0	90.3	26.2	26.2	97.3	0.8	94.0	0.4	0.0	0.4	0.0	26.2	26.2	99.8	0.8	94.8	0.8	0.0	26.2	26.3	99.9
	4	1.3	79.1	26.0	26.0	96.4	1.4	78.8	0.0	0.0	0.3	0.0	26.0	26.0	95.7	1.3	79.2	1.4	0.0	26.1	26.2	98.7
	5	1.0	86.3	25.8	25.9	98.6	1.0	86.8	0.0	0.1	0.0	0.0	25.8	25.9	98.7	1.1	85.2	1.3	0.0	26.0	26.0	96.8
	6	0.7	95.1	25.8	25.8	99.1	0.7	95.1	0.0	0.0	0.0	1.0	25.8	25.8	98.9	0.7	94.5	1.1	0.0	25.9	25.9	97.1
0.1	2	21.0	0.0	29.9	29.9	95.1	6.6	0.6	6.7	68.5	6.7	69.6	27.5	27.5	100.0	1.2	90.5	36.8	0.0	27.1	27.1	99.6
	3	3.2	31.2	26.9	27.0	95.7	1.6	79.9	3.4	94.6	3.4	94.1	26.8	26.9	95.7	1.8	74.4	12.0	29.4	27.0	27.0	99.0
	4	1.4	84.5	26.8	26.8	96.6	1.7	74.7	4.4	89.0	6.0	77.8	26.7	26.7	93.4	1.9	71.8	6.7	66.4	26.8	26.9	99.0
	5	1.1	85.5	26.5	26.5	97.5	1.1	85.1	98.5	100.0	23.8	15.1	26.5	26.6	81.4	1.4	76.8	5.7	79.1	26.6	26.6	98.9
	6	0.8	94.3	26.5	26.5	98.3	0.8	95.0	3.7	96.2	3.7	96.1	26.5	26.5	99.9	0.7	94.2	4.9	82.8	26.5	26.6	95.2
0.5	2	43.7	0.0	24.1	24.1	100.0	6.4	19.7	1.1	92.1	1.1	92.1	21.6	21.6	99.0	5.1	48.7	11.8	0.0	21.8	21.8	100.0
	3	1.9	87.6	21.3	21.4	98.4	2.8	70.1	1.3	81.3	1.3	87.5	21.2	21.3	98.9	2.5	75.9	1.4	82.9	21.3	21.4	99.8
	4	4.2	51.7	21.3	21.4	94.6	2.0	82.5	1.1	91.3	1.1	90.0	21.1	21.2	98.2	2.0	88.7	1.8	72.8	21.4	21.4	96.8
	5	1.2	90.6	21.0	21.1	93.2	1.1	93.6	36.7	96.5	3.3	53.4	21.0	21.1	91.1	1.1	93.7	1.0	92.3	21.1	21.1	97.8
	6	1.2	94.7	21.1	21.1	98.3	1.1	94.3	1.0	95.8	1.0	95.1	21.0	21.1	98.9	1.1	94.1	1.0	92.7	21.1	21.1	96.7
0.9	2	56.9	0.0	7.4	7.4	100.0	6.5	77.6	0.3	94.3	0.3	94.4	6.8	6.8	100.0	9.5	71.0	1.3	24.8	6.9	6.9	100.0
	3	3.3	94.6	6.7	6.8	99.8	4.1	90.7	0.5	82.0	0.3	93.5	6.7	6.7	97.2	3.2	94.1	0.3	93.1	6.7	6.8	100.0
	4	6.9	70.6	6.7	6.8	99.5	5.1	78.1	74.3	98.0	0.4	90.3	6.7	6.7	94.6	5.3	84.6	0.3	93.4	6.7	6.8	99.7
	5	2.7	91.6	6.7	6.7	99.7	2.6	94.0	4.5	99.2	0.4	91.0	6.7	6.7	93.3	2.5	93.8	0.3	94.8	6.7	6.7	99.5
	6	2.9	93.3	6.7	6.7	99.8	2.6	93.5	0.8	98.6	0.4	92.2	6.7	6.7	63.0	2.7	94.1	0.3	95.1	6.7	6.7	99.7

Table 1: Simulation results for baseline type 1. 'Cov.' stands for coverage and 'Con.' stands for convergence. AIC and BIC values are represented in thousands

cure fraction	df	non-cure AFT					mixture cure AFT									non-mixture cure AFT						
		% Bias beta	% Cov. beta	AIC	BIC	% Con.	% Bias beta	% Cov. beta	% Bias cure frac.	% Cov. cure frac.	% Bias surv t=20	% Cov. surv t=20	AIC	BIC	% Con.	% Bias beta	% Cov. beta	% Bias cure frac.	% Cov. cure frac.	AIC	BIC	% Con.
0.0	2	18.7	8.6	12.3	12.4	97.6	18.8	8.6	0.0	21.1	3.5	0.0	12.3	12.3	95.2	2,949.9	0.0	29.2	0.0	65.4	65.4	99.4
	3	3.6	85.9	4.3	4.3	99.6	3.4	87.3	0.0	50.0	0.3	0.0	4.6	4.7	63.8	69,195.0	6.0	29.8	0.0	222.8	222.9	48.1
	4	33.1	0.5	4.0	4.1	99.5										39.4	0.0	3.8	0.0	4.7	4.7	80.9
	5	24.8	0.0	2.3	2.4	100.0										26.3	0.0	5.1	0.0	7.3	7.4	40.6
	6	15.6	0.5	1.8	1.8	99.7	13.6	7.1	0.0	35.7	1.0	0.0	5.5	5.5	1.4	19.6	0.0	4.4	0.0	5.8	5.8	48.5
0.1	2	9.4	68.6	13.6	13.7	99.9	9.5	68.5	100.0	99.1	18.2	24.4	13.6	13.7	99.9	3,005.5	0.0	231.5	0.0	60.1	60.1	99.8
	3	8.0	56.4	6.8	6.8	99.8	5.2	84.8	100.0	89.7	65.9	0.0	2.0	2.1	16.5	21.1	2.4	77.1	0.0	8.5	8.6	75.7
	4	15.7	32.0	6.8	6.8	99.6										52.5	0.2	35.7	0.0	7.3	7.4	47.3
	5	31.4	0.0	5.1	5.1	99.4										29.9	0.2	45.0	0.0	7.8	7.8	46.2
	6	17.5	0.1	4.6	4.6	99.1	18.5	0.0	100.0	100.0	6.3	81.0	4.7	4.7	2.0	21.0	0.0	40.0	0.0	7.1	7.1	48.2
0.5	2	17.5	60.4	14.7	14.7	100.0	17.0	62.1	100.0	99.6	15.0	0.6	14.6	14.6	98.1	3,009.0	0.0	9.1	0.0	38.6	38.6	98.6
	3	34.7	1.7	11.7	11.7	99.9	10.5	0.0	6.2	0.0	6.2	0.0	21.6	21.6	0.1	8.2	65.9	4.8	4.4	11.3	11.3	99.2
	4	36.2	0.1	11.2	11.2	99.3	0.5	100.0	99.5	100.0	4.4	0.0	21.5	21.6	0.1	19.2	40.9	4.7	6.9	12.0	12.1	89.3
	5	39.1	0.1	10.6	10.6	97.2	3.8	100.0	2.6	100.0	4.4	0.0	21.5	21.5	0.1	30.8	0.2	5.4	0.0	11.6	11.6	45.8
	6	17.5	9.1	10.3	10.4	98.8	3.9	100.0	2.9	100.0	4.5	0.0	21.5	21.5	0.1	20.3	5.1	4.6	2.4	11.2	11.3	45.4
0.9	2	41.0	63.1	5.9	5.9	100.0	30.3	76.1	100.0	99.7	2.3	23.2	5.9	5.9	100.0	3,047.3	5.6	1.3	20.6	10.1	10.2	99.5
	3	49.7	30.3	5.4	5.4	100.0	10.8	79.1	0.4	87.6	0.4	78.0	7.4	7.4	13.8	26.6	44.7	0.4	77.2	5.4	5.4	95.6
	4	28.2	33.3	5.2	5.3	99.8	6.2	97.0	23.4	99.2	0.3	95.5	7.4	7.4	13.2	20.0	60.2	0.4	78.7	5.3	5.4	95.2
	5	36.5	20.1	5.2	5.2	99.7	7.1	94.6	0.4	99.1	0.3	95.5	7.4	7.4	11.1	28.3	22.3	0.6	65.5	5.3	5.3	99.1
	6	15.8	53.5	5.1	5.2	99.7	6.3	93.9	18.4	99.0	0.3	95.9	7.3	7.4	9.8	18.4	47.1	0.5	73.9	5.2	5.3	99.8

Table 2: Simulation results for baseline type 2. 'Cov.' stands for coverage and 'Con.' stands for convergence. AIC and BIC values are represented in thousands

cure fraction	df	non-cure AFT					mixture cure AFT									non-mixture cure AFT						
		% Bias beta	% Cov. beta	AIC	BIC	% Con.	% Bias beta	% Cov. beta	% Bias cure frac.	% Cov. cure frac.	% Bias surv t=20	% Cov. surv t=20	AIC	BIC	% Con.	% Bias beta	% Cov. beta	% Bias cure frac.	% Cov. cure frac.	AIC	BIC	% Con.
0.0	2	14.2	6.4	30.2	30.2	99.8	14.3	6.4	0.0	66.6	1.5	0.0	30.2	30.3	99.5	50.6	0.0	27.2	0.0	35.2	35.2	39.4
	3	9.9	22.7	29.4	29.4	99.5	5.1	58.5	1.4	0.0	1.6	0.0	30.5	30.5	20.7	22.4	0.1	17.0	0.0	30.3	30.3	91.1
	4	5.4	50.8	29.2	29.2	99.3	2.2	92.5	5.8	0.0	5.8	0.0	30.3	30.4	20.7	1.7	91.0	7.6	0.0	29.2	29.3	89.2
	5	4.3	62.7	29.1	29.1	98.5	2.2	90.1	5.3	0.0	5.3	0.0	30.3	30.4	20.5	1.8	92.2	7.2	0.0	29.3	29.3	83.3
	6	2.2	87.4	29.0	29.1	98.6	1.7	90.8	3.5	0.0	3.6	0.0	30.3	30.3	19.6	1.7	92.7	7.2	0.0	29.4	29.5	60.1
0.1	2	8.5	43.8	29.4	29.4	100.0	8.8	41.1	100.0	81.0	48.0	1.0	29.4	29.4	99.9	50.7	0.0	230.1	0.0	33.3	33.3	46.7
	3	4.6	75.1	28.7	28.7	99.6	5.7	64.9	9.5	84.5	11.0	77.0	29.7	29.7	26.5	20.6	0.0	134.5	0.0	29.2	29.2	96.6
	4	5.1	68.9	28.6	28.7	99.2	2.2	89.0	51.7	0.4	51.6	0.0	29.5	29.6	26.7	2.5	83.9	59.0	0.0	28.5	28.5	94.8
	5	6.2	42.5	28.4	28.5	98.8	2.4	95.3	47.0	0.0	47.1	0.0	29.5	29.6	26.0	1.9	96.7	59.1	0.0	28.6	28.6	85.8
	6	2.3	88.8	28.3	28.4	98.3	2.2	93.5	39.3	37.6	33.7	30.6	29.5	29.5	24.5	1.7	96.1	61.0	0.0	28.7	28.7	68.8
0.5	2	8.4	68.3	21.5	21.5	99.8	6.3	83.7	17.3	51.4	11.4	8.7	21.5	21.5	93.6	51.3	0.0	20.6	0.0	23.1	23.2	77.0
	3	8.1	72.4	21.2	21.2	100.0	7.5	74.4	1.4	96.9	1.4	95.8	21.7	21.7	47.7	14.6	23.7	9.7	0.0	21.3	21.3	98.5
	4	11.0	61.0	21.2	21.2	98.8	3.4	93.4	5.6	8.6	5.6	8.6	21.6	21.6	48.6	4.5	84.0	6.1	4.6	21.2	21.2	98.7
	5	7.3	62.9	21.0	21.0	99.6	6.2	73.8	92.4	65.0	4.6	38.4	21.6	21.6	46.6	3.5	92.7	6.4	3.2	21.5	21.6	70.8
	6	3.2	93.5	21.0	21.1	97.8	3.2	93.5	61.4	92.2	1.8	85.2	21.6	21.6	38.5	3.1	95.0	6.6	1.8	21.4	21.5	80.5
0.9	2	22.6	69.2	6.5	6.6	100.0	13.7	89.1	1.4	81.7	1.0	70.9	6.5	6.5	100.0	50.3	6.8	1.6	5.1	6.8	6.8	100.0
	3	13.4	91.7	6.5	6.6	100.0	11.2	90.0	0.4	97.4	0.4	97.0	6.5	6.6	86.1	11.8	83.1	0.8	64.9	6.5	6.5	99.6
	4	18.0	75.1	6.5	6.5	100.0	10.5	86.7	0.8	90.7	0.8	73.0	6.5	6.6	81.6	9.6	91.2	0.6	77.7	6.5	6.6	97.8
	5	9.6	87.4	6.5	6.5	100.0	9.8	88.6	88.2	97.7	0.5	89.8	6.5	6.6	81.4	8.7	92.3	0.7	77.1	6.5	6.6	99.9
	6	8.3	91.5	6.5	6.5	100.0	7.7	91.3	20.3	99.1	0.4	96.4	6.5	6.6	64.7	8.4	93.2	0.7	74.0	6.5	6.6	99.2

Table 3: Simulation results for baseline type 3. 'Cov.' stands for coverage and 'Con.' stands for convergence. AIC and BIC values are represented in thousands

cure fraction	df	non-cure AFT					mixture cure AFT									non-mixture cure AFT						
		% Bias beta	% Cov. beta	AIC	BIC	% Con.	% Bias beta	% Cov. beta	% Bias cure frac.	% Cov. cure frac.	% Bias surv t=20	% Cov. surv t=20	AIC	BIC	% Con.	% Bias beta	% Cov. beta	% Bias cure frac.	% Cov. cure frac.	AIC	BIC	% Con.
0.0	2	1.9	95.3	25.6	25.6	100.0	1.9	95.2	0.4	0.2	3.1	0.0	25.6	25.6	99.8	16.4	0.3	39.6	0.0	28.5	28.5	99.4
	3	2.0	95.7	25.6	25.6	100.0	2.0	95.8	0.7	0.0	3.2	0.0	25.6	25.7	99.3	3.5	71.8	27.6	0.0	26.6	26.7	99.7
	4	2.1	95.5	25.6	25.7	99.9	2.1	95.4	1.6	0.0	3.9	0.0	25.6	25.7	97.1	3.9	73.9	23.7	0.0	26.3	26.4	99.7
	5	2.1	95.1	25.6	25.7	100.0	2.1	95.1	3.6	0.0	5.1	0.0	25.6	25.7	95.7	2.4	94.2	22.7	0.0	26.2	26.3	99.5
	6	2.1	95.2	25.6	25.7	100.0	2.1	95.8	3.9	0.0	5.3	0.0	25.6	25.7	90.0	2.1	95.8	22.3	0.0	26.2	26.2	99.6
0.1	2	2.5	93.5	24.2	24.2	100.0	2.2	95.9	19.7	94.7	24.8	76.9	24.2	24.2	100.0	16.2	0.5	348.8	0.0	26.6	26.6	99.4
	3	2.2	96.1	24.2	24.2	100.0	2.2	96.3	28.8	92.5	27.3	78.6	24.2	24.2	99.8	3.0	81.0	241.3	0.0	25.0	25.1	99.9
	4	2.2	96.4	24.2	24.2	100.0	2.2	96.0	53.5	86.2	32.1	78.6	24.2	24.2	96.7	3.9	79.1	209.6	0.0	24.8	24.8	99.8
	5	2.1	95.7	24.2	24.2	100.0	2.1	95.6	59.6	84.6	32.7	81.2	24.2	24.2	96.8	2.5	94.0	201.7	0.0	24.7	24.8	99.9
	6	2.2	95.1	24.2	24.2	100.0	2.2	95.4	51.7	82.6	34.2	81.5	24.2	24.2	95.5	2.2	95.3	198.3	0.0	24.7	24.7	99.9
0.5	2	7.7	68.1	16.4	16.5	100.0	3.3	95.9	3.3	95.2	3.4	88.6	16.4	16.4	99.7	15.2	12.9	35.7	0.0	17.5	17.6	100.0
	3	3.6	96.5	16.4	16.4	100.0	3.3	96.1	4.6	94.1	3.9	88.2	16.4	16.4	99.2	3.0	93.3	24.7	0.0	16.9	16.9	100.0
	4	3.2	95.2	16.4	16.4	100.0	3.5	95.7	10.9	83.2	10.1	72.0	16.4	16.4	34.6	4.2	89.2	22.2	0.0	16.8	16.8	99.9
	5	3.3	96.1	16.4	16.4	100.0	3.4	94.0	65.6	82.4	11.9	69.9	16.3	16.4	31.9	3.7	95.2	21.6	0.0	16.7	16.8	99.9
	6	3.4	95.9	16.4	16.4	100.0	3.9	93.3	70.7	95.7	11.2	85.6	16.4	16.4	29.9	3.4	95.1	21.5	0.0	16.7	16.8	99.9
0.9	2	12.9	82.9	4.7	4.7	100.0	8.1	96.3	0.9	95.1	0.8	93.1	4.6	4.7	99.2	15.3	69.6	3.7	0.0	4.9	4.9	100.0
	3	9.0	95.9	4.6	4.7	100.0	8.7	93.7	1.1	90.4	1.1	80.5	4.5	4.6	33.8	7.1	95.7	2.6	0.2	4.8	4.8	99.9
	4	8.4	96.2	4.6	4.7	100.0	8.1	96.0	61.3	96.3	2.0	70.6	4.6	4.6	40.5	8.2	95.2	2.4	1.1	4.8	4.8	100.0
	5	8.6	95.7	4.6	4.7	100.0	8.6	95.8	60.1	96.9	1.7	77.8	4.6	4.7	45.0	9.4	93.7	2.4	1.2	4.8	4.8	99.9
	6	8.5	94.6	4.6	4.7	100.0	8.5	95.1	66.8	97.5	1.5	80.8	4.6	4.7	48.9	9.1	92.8	2.4	1.6	4.8	4.8	100.0

Table 4: Simulation results for baseline type 4. 'Cov.' stands for coverage and 'Con.' stands for convergence. AIC and BIC values are represented in thousands

```r
  compute_metrics()

df_aft2 <- clean_results |>
  filter(sce == 4, model == "non_cure_aft", df == 2) |>
  group_by(cure_frac) |>
  compute_metrics()

df_aft6 <- clean_results |>
  filter(sce == 4, model == "non_cure_aft", df == 6) |>
  group_by(cure_frac) |>
  compute_metrics()

# join
df_final <- Reduce(
  f = \(x, y) full_join(x, y, by = "cure_frac"),
  x = list(df_cox, df_aft2, df_aft6)
)

# only print in an interactive session
if (interactive()) {print(df_final)}
# save
dir.create("./results/tables/table_5/", recursive = TRUE)
write.csv(df_final, "./results/tables/table_5/table_5.csv",
          row.names = FALSE)

cat(
  df_final |>
    kable(
      format    = "latex",
      booktabs  = TRUE,
      align     = c("r", rep("r", ncol(df_final) - 1))
    ) |>
    add_header_above(c(
      " "                       = 1,
      "Cox PH"                  = 5,
      "non-cure AFT, df = 2" = 5,
      "non-cure AFT, df = 6" = 5
    )) |>
    kable_styling(
      latex_options = c("scale_down", "hold_position"),
      font_size     = 8
    ),
  sep = "\n"
)
```

	Cox PH					non-cure AFT, df = 2					non-cure AFT, df = 6				
cure_frac	% Bias beta.x	% Cov. beta.x	AIC.x	BIC.x	% Converged.x	% Bias beta.y	% Cov. beta.y	AIC.y	BIC.y	% Converged.y	% Bias beta	% Cov. beta	AIC	BIC	% Converged
0.0	1.98	95.6	65.00	65.00	100	1.91	95.3	25.60	25.60	100	2.06	95.2	25.60	25.70	100
0.1	5.55	56.3	58.80	58.80	100	2.49	93.5	24.20	24.20	100	2.19	95.1	24.20	24.20	100
0.5	20.40	0.2	33.40	33.40	100	7.69	68.1	16.40	16.50	100	3.40	95.9	16.40	16.40	100
0.9	29.30	12.5	6.79	6.79	100	12.90	82.9	4.65	4.67	100	8.54	94.6	4.64	4.69	100

We transformed it to LaTeX:

cure fraction	Cox PH					non-cure AFT, df = 2					non-cure AFT, df = 6				
	% Bias beta	% Cov. beta	AIC	BIC	% Con.	% Bias beta	% Cov. beta	AIC	BIC	% Con.	% Bias beta	% Cov. beta	AIC	BIC	% Con.
0.0	2.0	95.6	65.0	65.0	100.0	1.9	95.3	25.6	25.6	100.0	2.1	95.2	25.6	25.7	100.0
0.1	5.5	56.3	58.8	58.8	100.0	2.5	93.5	24.2	24.2	100.0	2.2	95.1	24.2	24.2	100.0
0.5	20.4	0.2	33.4	33.4	100.0	7.7	68.1	16.4	16.5	100.0	3.4	95.9	16.4	16.4	100.0
0.9	29.3	12.5	6.8	6.8	100.0	12.9	82.9	4.7	4.7	100.0	8.5	94.6	4.6	4.7	100.0

Table 5: Comparison of Cox PH and non-cure AFT (df = 2 and 6) in estimating log hazard ratios (Cox PH) and log acceleration factors (AFT) for baseline type 4 (standard Weibull). AIC and BIC values are represented in thousands

Table 6: Simulation results for time-varying acceleration factors

```r
# filter out errors and reshape to have rows for times 2,4,..,10
res_tvc <- results_TD |>
  filter(is.na(err_msg)) |>
  select(-MISE_inf) |>
  pivot_longer(
    cols = matches("^(bias_af|bias_relative_af|coverage_af|MISE)_\\d+"),
    names_to = c(".value", "time"),
    names_pattern = "(bias_af|bias_relative_af|coverage_af|MISE)_(\\d+)") |>
  mutate(
    time = as.numeric(time),
    convergence = convergence == "TRUE" & !NA_vcov & !neg_diag_vcov & sum_gradients < 1
  ) |>
  filter(sce == "linear time")

dat_TVC <- res_tvc |>
  group_by(model, df, time) |>
  summarise(
    conv = 100 * mean(convergence, na.rm = TRUE),
    bcf  = abs(median(bias_relative_cure_frac[convergence], na.rm = TRUE)),
    ccf  = 100 * mean(coverage_cure_frac[convergence], na.rm = TRUE),
    baf  = abs(median(bias_relative_af[convergence], na.rm = TRUE)),
    caf  = 100 * mean(coverage_af[convergence], na.rm = TRUE),
    mise = median(MISE[convergence], na.rm = TRUE),
    AIC  = median(AIC[convergence], na.rm = TRUE) / 1000,
    BIC  = median(BIC[convergence], na.rm = TRUE) / 1000,
    .groups = "drop"
  )
# build the flextable
tab <- tabulator(
  x       = dat_TVC,
  rows    = c("df", "time"),
  columns = "model",
  `% Con.`                = as_paragraph(conv),
  `% bias cure frac`      = as_paragraph(bcf),
  `coverage cure frac`    = as_paragraph(ccf),
  `% bias AF`             = as_paragraph(baf),
```

```r
  `coverage AF`          = as_paragraph(caf),
  `MISE`                 = as_paragraph(mise),
  `AIC (*1000)`          = as_paragraph(AIC),
  `BIC (*1000)`          = as_paragraph(BIC)
)

ft <- as_flextable(tab) |>
  vline(border = fp_border(color = "black")) |>
  theme_booktabs()

# save:
  dir.create("./results/tables/table_6/", recursive = TRUE)
  # as xlsx through "flexlsx" package
  save_flextable_xlsx(ft, dir = "./results/tables/table_6/table_6.xlsx")
  # convert to .csv via "rio" package
  convert("./results/tables/table_6/table_6.xlsx",
          "./results/tables/table_6/table_6.csv",
          in_opts = list(.name_repair = "minimal"))
# display only if in interactive session, does not fit to pdf in this format
if (interactive()) {print(ft)}
```

Again this table is too wide to include in the PDF in this format. To view it at full size, please run the above chunk interactively in R. We have translated this table into LaTeX and include it below.

	Acceleration factor on cumulative scale									Cox and Oakes time-varying AFT								
df	% Con.	% Bias cure frac.	% Cov. cure frac.	AIC	BIC	time	% Bias acc. factor	% Cov. acc. factor	MISE	% Con.	% Bias cure frac.	% Cov. cure frac.	AIC	BIC	time	% Bias acc. factor	% Cov. acc. factor	MISE
2	98.4	0.2	96.6	4.6	4.6	2	10.0	95.5	0.0	95.3	0.1	94.3	4.6	4.6	2	0.5	94.3	0.0
						4	5.1	84.9	0.1						4	0.6	95.2	0.1
						6	12.2	89.7	0.6						6	1.5	95.7	0.6
						8	24.9	92.3	1.8						8	1.1	94.1	1.8
						10	39.4	71.8	4.8						10	0.1	94.0	4.8
3	43.6	0.4	90.8	4.6	4.7	2	13.2	95.0	0.0	17.1	1.0	93.1	4.6	4.7	2	3.6	92.5	0.0
						4	4.0	77.5	0.1						4	2.7	91.4	0.1
						6	10.1	81.9	0.6						6	2.7	93.1	0.6
						8	23.0	91.5	1.8						8	2.2	89.6	1.8
						10	37.2	75.2	4.8						10	0.0	89.7	4.8
4	74.5	14.4	89.3	4.6	4.6	2	12.1	96.3	0.0	73.3	1.4	85.3	4.6	4.6	2	1.7	94.7	0.0
						4	5.0	84.0	0.1						4	0.3	95.5	0.1
						6	12.3	88.7	0.6						6	0.3	95.8	0.6
						8	24.5	92.7	1.8						8	0.7	94.2	1.8
						10	38.8	73.3	4.8						10	1.0	94.8	4.8
5	66.8	59.2	93.9	4.6	4.6	2	11.2	95.1	0.0	60.1	45.8	87.0	4.6	4.6	2	0.0	94.7	0.0
						4	4.4	80.9	0.1						4	1.4	95.1	0.1
						6	11.3	84.0	0.6						6	1.5	96.9	0.6
						8	24.1	92.4	1.8						8	1.1	92.8	1.8
						10	38.2	73.4	4.8						10	3.6	94.2	4.8
6	79.3	9.3	83.3	4.6	4.7	2	9.7	96.2	0.0	78.2	4.9	83.6	4.6	4.7	2	0.0	94.7	0.0
						4	4.4	81.9	0.1						4	1.4	94.9	0.1
						6	11.8	88.5	0.6						6	0.7	94.5	0.6
						8	24.5	92.4	1.8						8	1.1	93.5	1.8
						10	39.0	71.5	4.8						10	0.7	94.5	4.8

Table 6: Simulation results for time-varying acceleration factors. 'Cov.' stands for coverage and 'Con.' stands for convergence. AIC and BIC values are represented in thousands

Figure 3: Non-cure AFT fit for Scenario 1 with a cure fraction of 0.9: Log-cumulative hazard on the log-time scale, comparing estimated and true generating processes

```r
# Fit AFT with df = 6 not adjusted for cure
fit   <- aft(
  Surv(observed_time, delta) ~ X, data = dat_vector_sce_1_cf_0.9[[3]], df = 6)

knots    <- c(fit@args$boundaryKnots[1],
              fit@args$interiorKnots,
              fit@args$boundaryKnots[2])

log_time <- seq(knots[1], log(30), length.out = 4000)

# Estimate logH from the fit
df_est <- data.frame( log_time = log_time,
  logH_est = log(
    predict(
      fit, type    = "cumhaz",
      newdata = data.frame(observed_time = exp(log_time), X = 0)
    )
  )
)

# True logH
df_true <- data.frame(
  log_time  = log_time,
  logH_true = log(-log(theoretical_survival(1, exp(log_time), X = 0, 0.9)))
)

# Plot both curves for true and estimated logH
plot_logH <- ggplot() +
  geom_line(data = df_est, aes(log_time, logH_est,  color = "Estimated"),
            size = 0.5) +
  geom_line(data = df_true, aes(log_time, logH_true, color = "Truth"),
            size = 0.5) +
  geom_vline(xintercept = knots, linetype = 2, color = "gray40") +
  scale_color_manual("", values = c(Estimated = "#0072B2", Truth = "#D55E00")) +
  labs(x = "Log(Time)", y = "Log Cumulative Hazard") +
  base_theme +
  theme(legend.position = "bottom",
        legend.text     = element_text(size = 20, family = "LM Roman 10")) +
  #text for knot locations:
  annotate("label", x = 0.3, y = -7,
           label = "Internal knots",
           hjust = 0,
           family = "LM Roman 10",
           size = 10)  +
    annotate("text", x = 2.5, y = -4,
           label = "Right \nboundary\nknot",
           hjust = 0,
           family = "LM Roman 10",
```

```
             size = 6) +
    annotate("text", x = -1.5, y = -4,
             label = "Left \nboundary \nknot",
             hjust = 0,
             family = "LM Roman 10",
             size = 6)
#print and save
if(interactive()){print(plot_logH)}
# figures look better in saved versions, please see ./results/figures/
ggsave(
  "./results/figures/Figure_3.pdf",
  plot   = plot_logH,
  device = cairo_pdf,
  width  = 300,
  height = 200,
  units  = "mm"
)
```

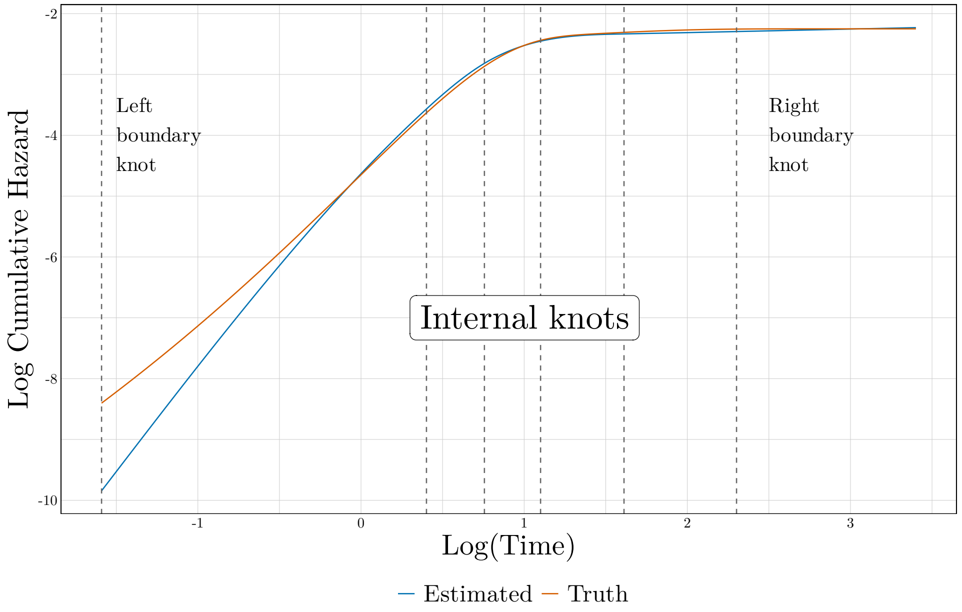

Figure 3: Non-cure AFT fit for Scenario 1 with a cure fraction of 0.9: Log-cumulative hazard on the log-time scale, comparing estimated and true generating processes

Figure 4: Non-cure AFT fit for Scenario 1 with a cure fraction of 0.9: Survival curves comparing estimated and true generating processes

```
# Estimated survival from the aft fit (df=6, no cure adjustment)
#    (`fit` from previous chunk)
```

```r
time <- seq(0.1, 30, length.out = 1000)
pred  <- predict(fit, type = "surv",
                 newdata = data.frame(observed_time = time, X = 0),
                 se.fit  = TRUE)
df_est <- data.frame(
  time     = time,
  estimate = pred$Estimate,
  lower    = pred$lower,
  upper    = pred$upper
)
# True survival from generating process
df_true <- data.frame(
  time = time,
  surv = theoretical_survival(1, time, X = 0, cf = 0.9)
)
# Plot the two survival curves
plot_S <- ggplot() +
  geom_line(data = df_true, aes(time, surv, color = "Truth"), size = 0.5) +
  geom_line(data = df_est, aes(time, estimate, color = "Estimated"),
            size = 0.5) +
    # Estimated 95% confidence bands (dashed blue lines; not mapped to legend)
    geom_line(data = df_est, aes(x = time, y = lower),
              color = "#0072B2", linetype = "dashed", size = 0.5,
              show.legend = FALSE) +
    geom_line(data = df_est, aes(x = time, y = upper),
              color = "#0072B2", linetype = "dashed", size = 0.5,
              show.legend = FALSE) +
    # Vertical line at t = 10 for end of follow-up
    geom_vline(aes(xintercept = 10, color = "End of follow-up"),
               linetype = "dashed", size = 0.5, show.legend = FALSE) +
   annotate("text", x = 10.5, y = 0.95, label = "End of follow-up",
            hjust = 0, family = "LM Roman 10", size = 6) +
  scale_color_manual("", values = c(Truth = "#D55E00", Estimated = "#0072B2")) +
  scale_x_continuous(labels = label_number(accuracy = 0.1)) +
  scale_y_continuous(limits = c(0.88, 1), labels =
                       label_number(accuracy = 0.01)) +
  labs(x = "Time", y = "Survival Probability") +
  base_theme +
  theme(
    legend.position = "bottom",
    legend.text     = element_text(size = 20, family = "LM Roman 10")
  )
# print and save
if(interactive()){print(plot_S)}
# figures look better in saved versions, please see ./results/figures/
ggsave(
  "./results/figures/Figure_4.pdf",
  plot   = plot_S,
  device = cairo_pdf,
  width  = 300,
  height = 200,
  units  = "mm"
)
```

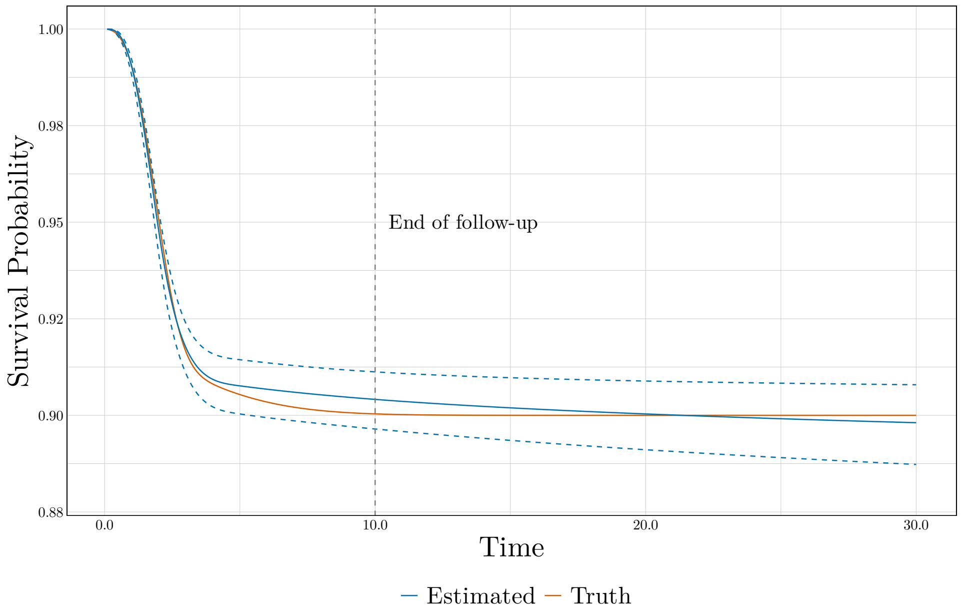

Figure 4: Non-cure AFT fit for Scenario 1 with a cure fraction of 0.9: Survival curves comparing estimated and true generating processes

Figure 5: Survival curves for the same dataset from two fits of the model using different initial values, compared with the true generating process

```
# fit mixture cure AFT model to dataset generated with cure fraction = 0.9
fit1 <- aft(
  Surv(observed_time, delta) ~ X,
  data = dat_vector_sce_1_cf_0.9[[2]],
  df = 4,
  mixture = TRUE
)
# fit same mixture cure model but with a set of different initial values
# to the same dataset
fit1_init <- aft(
  Surv(observed_time, delta) ~ X,
  data = dat_vector_sce_1_cf_0.9[[2]],
  df = 4,
  mixture = TRUE,
  init = c(
    "X" = 1.11828576033048,
    "cure.(Intercept)" = -0.577276363082236,
    "nsx(logtstar, df, intercept = TRUE)1" = 3.25044989814831,
    "nsx(logtstar, df, intercept = TRUE)2" = 7.56830621681516,
    "nsx(logtstar, df, intercept = TRUE)3" = -26.1178397471224,
    "nsx(logtstar, df, intercept = TRUE)4" = 13.2790868936077
```

```r
  )
)
# Cure fraction estimate from first fit:
plogis(unname(coef(fit1)[2]))
```

```
## [1] 0.9044196
```

```r
# Cure fraction estimate from fit with different init vals:
plogis(unname(coef(fit1_init)[2]))
```

```
## [1] 0.359559
```

```r
# Let's plot estimated survivals from these two fits and compare them to the
# true generating process
time <- seq(0.1, 30, length.out = 100)
# Estimated survival from fit with estimated cure frac = 0.36
df_fit1 <- data.frame(x = time,
                      y = predict(fit1_init, type = "surv",
                                  newdata = data.frame(X = 0,
                                                       observed_time = time)),
                      group = "Initial Values 1, cure = 0.36")
df_fit2 <- data.frame(x = time,
                      y = predict(fit1, type = "surv",
                                  newdata = data.frame(X = 0,
                                                       observed_time = time)),
                      group = "Initial Values 2, cure = 0.90")
# True survival from generating process
df_true <- data.frame(
  x = time,
  y = theoretical_survival(sce = 1, time, X = 0, cf = 0.9),
  group = "Truth, cure = 0.9"
)
# plot
color_map <- c("Truth, cure = 0.9" = "red",
               "Initial Values 1, cure = 0.36" = "#0072B2",
               "Initial Values 2, cure = 0.90" = "#D55E00")
plot_3Survs = ggplot(rbind(df_true, df_fit1, df_fit2),
                     aes(x = x, y = y, color = group)) +
  geom_line(linewidth = 0.5) +
  geom_vline(xintercept = 10, linetype = "dashed", colour = "black") +
  annotate("text", x = 10.5, y = 0.95,
           label = "End of follow-up",
           hjust = 0, family = "LM Roman 10", size = 6) +
  scale_color_manual(values = color_map) +  # assign colors according to map
  labs(x = "Time",
       y = "Survival Probability",
       color = "") + base_theme +
  theme(legend.position = "bottom",
        legend.text     =
          element_text(size = 20, color = "black", family = "LM Roman 10"),
        legend.title    =
          element_text(size = 0, color = "black", family = "LM Roman 10"),)
```

```r
# print and save
if(interactive()){print(plot_3Survs)}
# figures look better in saved versions, please see ./results/figures/
ggsave(
  "./results/figures/Figure_5.pdf",
  plot   = plot_3Survs,
  device = cairo_pdf,
  width  = 300,
  height = 200,
  units  = "mm"
)
```

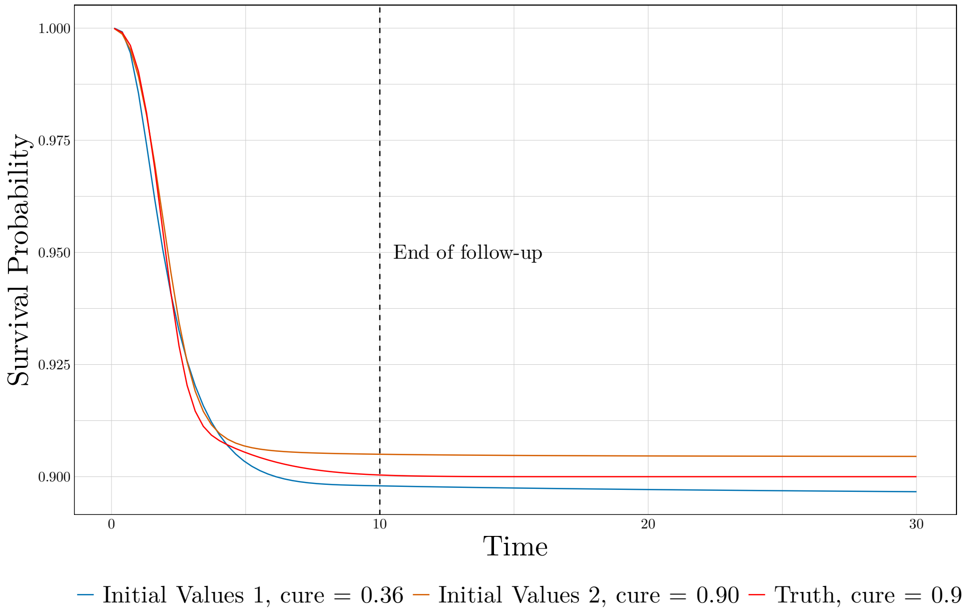

Figure 5: Survival curves for the same dataset from two fits of the model using different initial values, compared with the true generating process

Table 7: Baseline Characteristics Stratified by Sex

```r
# synthetic, population-based cancer registry dataset
colon <- biostat3::colon

# Table 1 by sex
table1_sex <- CreateTableOne(vars = c("age", "stage", "surv_yy", "status"),
                             factorVars = c("stage", "status"), data = colon,
                             strata = "sex", addOverall = TRUE)

# save
to_save <- print(table1_sex, printToggle = FALSE)
dir.create("./results/tables/table_7/", recursive = TRUE)
```

```r
write.csv(to_save, file = "./results/tables/table_7/table_7.csv")
# print in this form if in interactive session
if(interactive()){print(table1_sex)}
```

We transformed it to LatTeX:

Table 7: Baseline Characteristics Stratified by Sex

Characteristic	Overall (n = 15,564)	Female (n = 9,224)	Male (n = 6,340)
**Age, Mean (SD)**	69.13 (12.42)	70.61 (12.12)	66.98 (12.54)
**Stage at Diagnosis, n (%)**			
Localised	6,274 (40.3%)	3,654 (39.6%)	2,620 (41.3%)
Regional	1,787 (11.5%)	1,072 (11.6%)	715 (11.3%)
Distant Metastasis	5,147 (33.1%)	3,027 (32.8%)	2,120 (33.4%)
Unknown	2,356 (15.1%)	1,471 (15.9%)	885 (14.0%)
**Survival Time (years), Mean (SD)**	3.82 (4.40)	3.89 (4.51)	3.73 (4.23)
**Status at Last Follow-Up, n (%)**			
Alive	4,642 (29.8%)	2,765 (30.0%)	1,877 (29.6%)
Dead: Colon Cancer	8,369 (53.8%)	5,048 (54.7%)	3,321 (52.4%)
Dead: Other Causes	2,549 (16.4%)	1,409 (15.3%)	1,140 (18.0%)
Lost to Follow-Up	4 (0.0%)	2 (0.0%)	2 (0.0%)

Figure 6: Predicted survival from a flexible parametric AFT model with four degrees of freedom adjusted for sex compared with the Kaplan-Meier estimates stratified by sex

```r
colon = transform(colon,
                  male=0+(sex=="Male"),
                  Unknown=0+(stage=="Unknown"),
                  Localised=0+(stage=="Localised"),
                  Regional=0+(stage=="Regional"),
                  Distant=0+(stage=="Distant"))
localised = subset(colon, stage=="Localised")
# maximum survival
max(colon$surv_yy)
```

```
## [1] 20.5
```

```r
# fit aft not adjusted for cure
fit <- aft(Surv(surv_mm, status=="Dead: cancer") ~ male, df=4, data=localised)
# plot and comapre with KM
par(mfrow=1:2)
par(oma = c(3, 3, 0, 0), mar = c(1,1,6,3))
par(family = "LM Roman 10")
# for males
plot(fit, newdata=data.frame(male=1), type="surv",
     main="Males",
     ylab = "",
     xlab = "",
     ylim=c(0.5,1))
```

```r
lines(survfit(Surv(surv_mm, status=="Dead: cancer") ~ 1, data=localised,
              subset=(male==1)))
# for females
plot(fit, newdata=data.frame(male=0), type="surv",
     main="Females",
     ylab = "",
     xlab = "",
     ylim=c(0.5,1))
lines(survfit(Surv(surv_mm, status=="Dead: cancer") ~ 1, data=localised,
              subset=(male==0)))
# axis names
mtext('Time since cancer diagnosis (months)', side = 1, outer = TRUE, line = 2)
mtext('Survival probability', side = 2, outer = TRUE, line = 2)
```

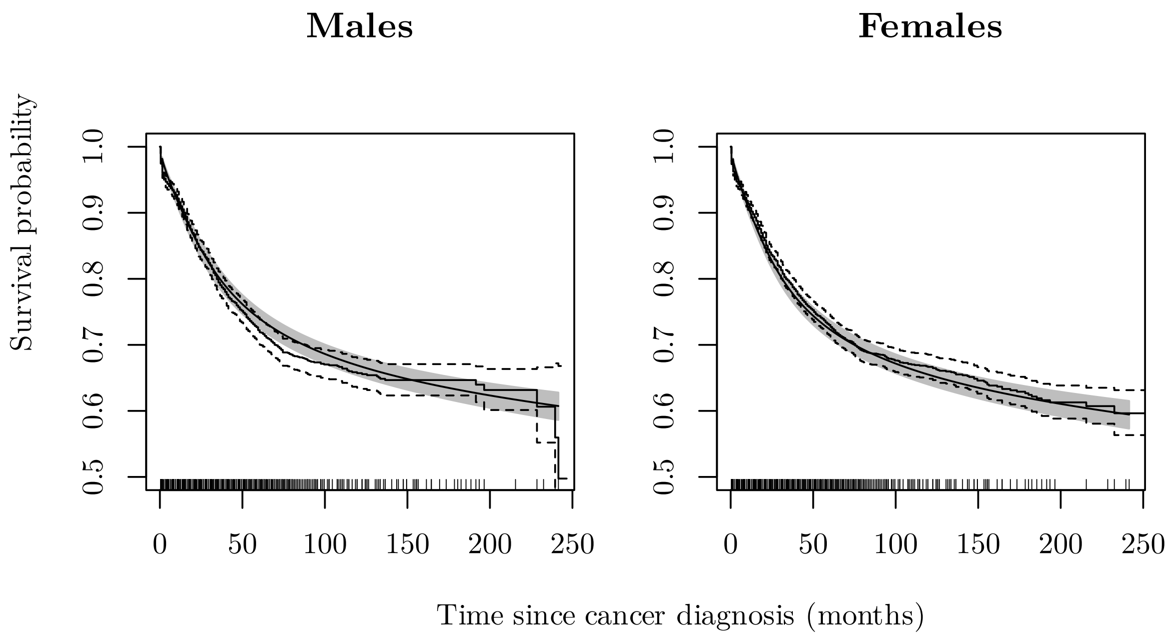

Figure 6: Predicted survival from a flexible parametric AFT model with four degrees of freedom adjusted for sex compared with the Kaplan-Meier estimates stratified by sex

Figure 7: Time-varying acceleration factor for males compared with females diagnosed with localised colon cancer at age 70 years, colon cancer

```r
# aft not adjusted for cure, up to 10 years of follow-up,
# time-varying acceleration factor (cumulative formulation) for effect of sex
fit <- aft(Surv(surv_mm, status=="Dead: cancer") ~ ns(age,df=4)+male,
           data=localised[localised$surv_mm<120,], df=3, tvc=list(male=3),
```

```
           tvc.intercept=FALSE)
# same model, time-varying acceleration factor is in integrated formulation
fit_integrated <- aft(Surv(surv_mm, status=="Dead: cancer") ~ ns(age,df=4)+male,
                      data=localised[localised$surv_mm<120,], df=3,
                      tvc=list(male=3),
                    tvc.integrated = TRUE, tvc.intercept=FALSE)
# plot time-varying acceleration factors
par(mfrow=c(1,2), oma = c(3,3,0,0), mar = c(1,1,6,3))
par(family = "LM Roman 10")
plot(fit, type="accfac", newdata=data.frame(age=70, male=0), ylim=c(0,2),
     var="male", main="Male, \n acceleration factor \n on a cumulative scale",
     ylab ="", xlab ="", pty = "m")
plot(fit_integrated, type="accfac", newdata=data.frame(age=70, male=0),
     ylim=c(0,2), var="male",
     main="Male, \n Cox and Oakes \n time-dependent AFT", ylab ="", xlab ="",
     pty = "m")
mtext('Time since cancer diagnosis (months)', side = 1, outer = TRUE, line = 2)
mtext('Acceleration factor', side = 2, outer = TRUE, line = 2)
```

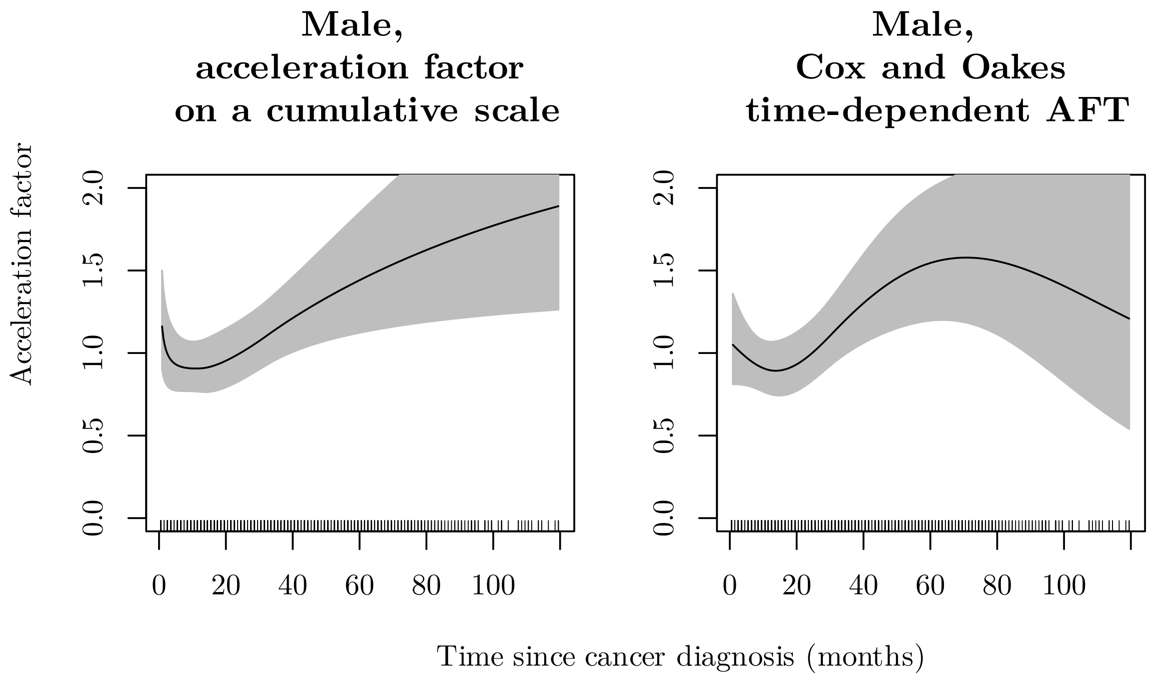

Figure 7: Time-varying acceleration factor for males compared with females diagnosed with localised colon cancer at age 70 years, colon cancer

Figure 8: Time-varying acceleration factor for males diagnosed with distant colon cancer

```r
# aft not adjusted for cure, time-varying acceleration factor (cumulative form.)
# for Distant metastasis
fit <- aft(Surv(surv_mm, status=="Dead: cancer") ~ ns(age,df=2)+male+Distant,
           data=colon, df=4, tvc=list(Distant=3), tvc.intercept=FALSE)
# same model but acceleration factors on integrated formulation
fit_integrated <- aft(Surv(surv_mm, status=="Dead: cancer") ~ ns(age,df=2) +
                        male+Distant,
            data=colon, df=4, tvc=list(Distant=3),
            tvc.intercept=FALSE, tvc.integrated = TRUE)
# plot acceleration factors
par(mfrow=c(1,2), oma = c(4,5,0,0), mar = c(1,1,6,3))
par(family = "LM Roman 10")
plot(fit, type="accfac", newdata=data.frame(age=70, male=0, Distant=0),
     var="Distant",
     main="Distant metastasis, \n acceleration factor \n on a cumulative scale",
     ylab = "", xlab = "")
plot(fit_integrated, type="accfac",
     newdata=data.frame(age=70, male=0, Distant=0), var ="Distant",
     main="Distant metastasis, \n Cox and Oakes \n time-dependent AFT",
     ylab = "", xlab = "")
mtext('Time since cancer diagnosis (months)', side = 1, outer = TRUE, line = 2)
mtext('Acceleration factor', side = 2, outer = TRUE, line = 2)
```

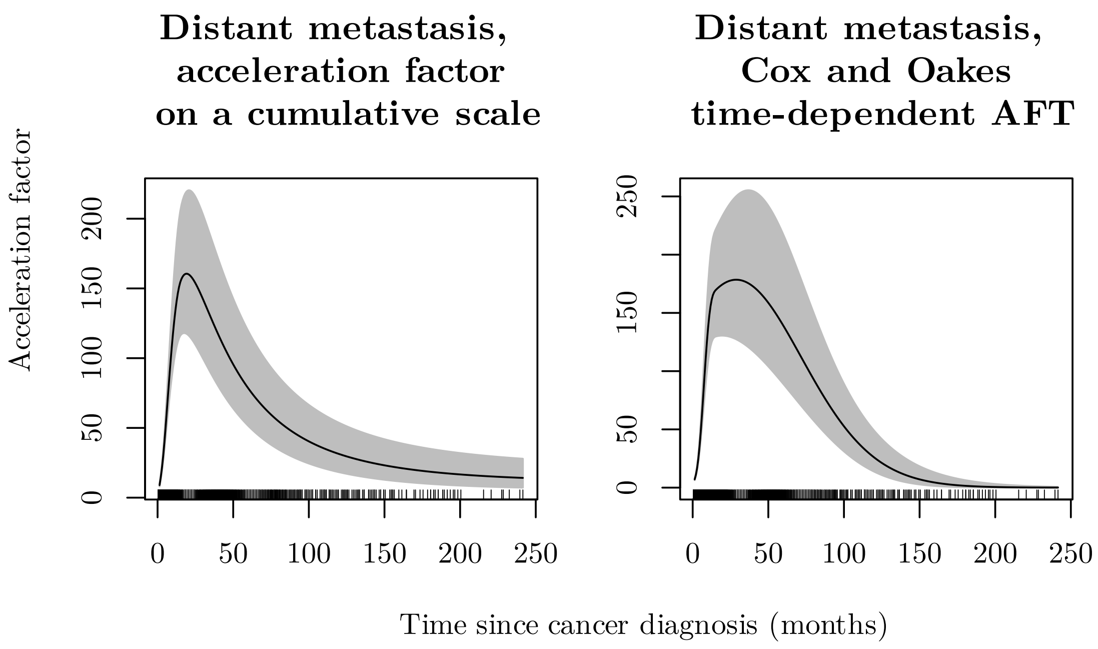

Figure 8: Time-varying acceleration factor for males diagnosed with distant colon cancer (synthetic population-based cancer registry)

Figure 9: Time-varying acceleration factor for males diagnosed with distant colon cancer accounting for cure

```
# mixture cure aft, cure fraction varies by distant metastasis status,
# time-varying acceleration factor for distant metastasis
fit <- aft(Surv(surv_mm, status == "Dead: cancer")~ Distant + ns(age,3),
              cure.formula = ~Distant, data = colon, mixture = TRUE,
              tvc = list(Distant = 2), tvc.intercept = FALSE,
              tvc.integrated = TRUE, df = 3)
# cure fraction for patients without distant metastasis
plogis(unname(coef(fit)[7]))
```

```
## [1] 0.4108218
```

```
# cure fraction for patiens with distant metastasis
plogis(unname(coef(fit)[7]+coef(fit)[8]))
```

```
## [1] 0.06424982
```

```
# plot the acceleration factor
par(mfrow=c(1,1))
par(family = "LM Roman 10")
plot(fit, type = "accfac", newdata = data.frame(Distant = 0, age = 70),
     var = "Distant", add = FALSE,
     ylab = "Acceleration factor",
     xlab = 'Time since cancer diagnosis (months)',
     main =
       "Distant metastasis, Cox and Oakes \n time-dependent mixture cure AFT")
```

Table 9 (Appendix): Relative bias in the estimation of log acceleration factor for non-cure AFT model for baseline type 4

```
# relative bias beta (%) by degrees of freedom and by cure fraction
# for aft model not adjusted for cure

# args: sce_num (scenario);
# returns: flextable  of median abs. beta bias by df(rows) and cure frac. (cols)
bias_beta_table <- \(sce_num) {
  dat <- clean_results |>
    filter(sce == sce_num, model == "non_cure_aft") |>
    group_by(cure_frac, df) |>
    summarise(
      across(where(is.numeric),
        list( avg = ~ median(abs(.x), na.rm = TRUE))), .groups = "drop")

  tab <- tabulator(x = dat, rows = "df", columns = "cure_frac",
    `% bias beta` = as_paragraph(bias_relative_beta_avg))
```

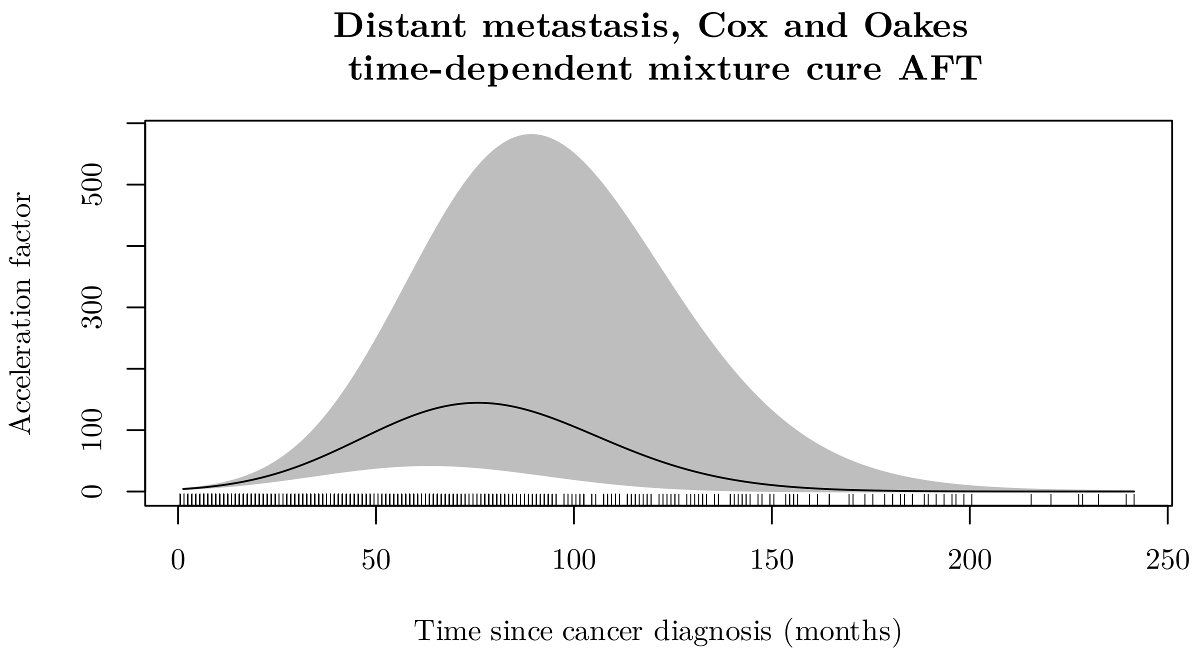

Figure 9: Time-varying acceleration factor for males diagnosed with distant colon cancer accounting for cure (synthetic population-based cancer registry)

```
  ft <- as_flextable(tab) |> theme_booktabs()
    # save
  subdir <- ifelse(sce_num == 4 ,
                   "./results/tables/table_9/",
                   "./results/tables/table_10/")
  filename <- ifelse(sce_num == 4 ,
                   "table_9",
                   "table_10")
  xlsx_file <- file.path(subdir, paste0(filename, ".xlsx"))
  csv_file  <- file.path(subdir, paste0(filename, ".csv"))

  dir.create(subdir, recursive = TRUE)
  # as xlsx through "flexlsx" package
  save_flextable_xlsx(ft, dir = xlsx_file)
  # convert to .csv via "rio" package
  convert(xlsx_file, csv_file,
          in_opts = list(.name_repair = "minimal"))
  # print
  ft
  }
bias_beta_table(4)
```

df	0	0.1	0.5	0.9
2	1.9	2.5	7.7	12.9

df	0	0.1	0.5	0.9
3	2.0	2.2	3.6	9.0
4	2.1	2.2	3.2	8.4
5	2.1	2.1	3.3	8.6
6	2.1	2.2	3.4	8.5

We translated it to LaTeX:

	**Cure fraction**			
**df**	0	0.1	0.5	0.9
2	1.9	2.5	7.7	12.9
3	2.0	2.2	3.6	9.0
4	2.1	2.2	3.2	8.4
5	2.1	2.1	3.3	8.6
6	2.1	2.2	3.4	8.5

Table 9: Relative bias (%) in the estimation of log acceleration factor for non-cure AFT model for baseline type 4 (standard Weibull).

Table 10 (Appendix): Relative bias (%) in the estimation of log acceleration factor for non-cure AFT model for baseline type 2

```
# relative bias beta (%) by degrees of freedom and by cure fraction
# for aft model not adjusted for cure, for sce = 2,
# reusing function from previous chunk
bias_beta_table(2)
```

df	0	0.1	0.5	0.9
2	18.6	9.5	17.5	41.0
3	3.6	8.0	34.7	49.7
4	33.1	15.7	36.2	28.2
5	24.8	31.4	38.8	36.4
6	15.6	17.5	17.5	15.9

We translated it to LaTeX:

	**Cure fraction**			
**df**	0	0.1	0.5	0.9
2	18.7	9.4	17.5	41.0
3	3.6	8.0	34.7	49.7
4	33.1	15.7	36.2	28.2
5	24.8	31.4	39.1	36.5
6	15.6	17.5	17.5	15.8

Table 11: Relative bias (%) in the estimation of log acceleration factor for non-cure AFT model for baseline type 2.

Figure 10 (Appendix): Relative bias in cure fraction (%) for mixture cure AFT model, baseline type 1, cure fraction 0.5

```
converged <- clean_results |> filter(convergence == TRUE)
# plot relative bias in cure fraction, sce = 1, cure frac = 0.5
jitter <- converged |>
  filter(model == "mixture_cure" & cure_frac == 0.5 & sce == 1) |>
  ggplot( aes(x = factor(df), y= bias_relative_cure_frac, fill= df)) +
  scale_fill_viridis(discrete = FALSE, alpha=0.6) +
  geom_jitter(color="black", size=0.30, alpha=0.65) +
  base_theme + theme(panel.border = element_blank())+
  xlab("Degrees of freedom") +
  ylab("Relative bias in cure fraction (%)") + ylim(c(-100,10))
# print and save
if(interactive()){print(jitter)}
# figures look better in saved versions, please see ./results/figures/
ggsave(
  "./results/figures/Figure_10.pdf",
  plot = jitter,
  device = cairo_pdf,
  width  = 300,
  height = 200,
  units  = "mm"
)
```

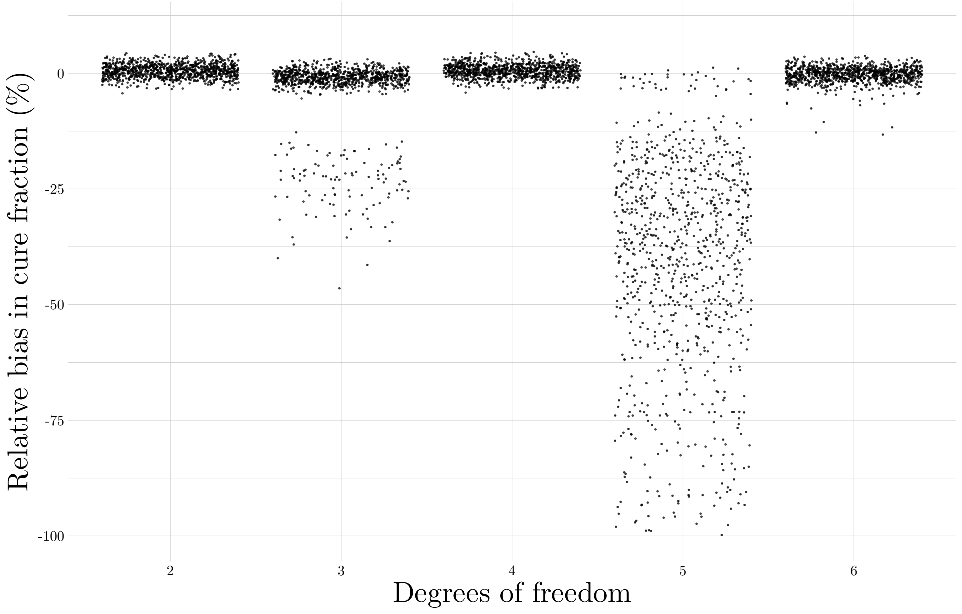

Figure 10: Relative bias in cure fraction (%) for mixture cure AFT model, baseline type 1, cure fraction 0.5.

Figure 11 (Appendix): Relative bias in cure fraction (%) for mixture cure AFT model, baseline type 3, cure fraction 0.9

```r
# Mixture cure, cf=0.9, sce=3
jitter <- converged |>
  filter(model == "mixture_cure", cure_frac == 0.9, sce == 3) |>
  ggplot(aes(x = factor(df), y = bias_relative_cure_frac, fill = df)) +
    scale_fill_viridis(discrete = FALSE, alpha = 0.6) +
    geom_jitter(color = "black", size = 0.3, alpha = 0.65) +
    base_theme +
    theme(panel.border = element_blank()) +
    xlab("Degrees of freedom") +
    ylab("Relative bias in cure fraction (%)") +
    ylim(-100, 10)
# print and save
if(interactive()){print(jitter)}
# figures look better in saved versions, please see ./results/figures/
ggsave(
  "./results/figures/Figure_11.pdf",
  plot   = jitter,
  device = cairo_pdf,
  width  = 300,
  height = 200,
  units  = "mm"
)
```

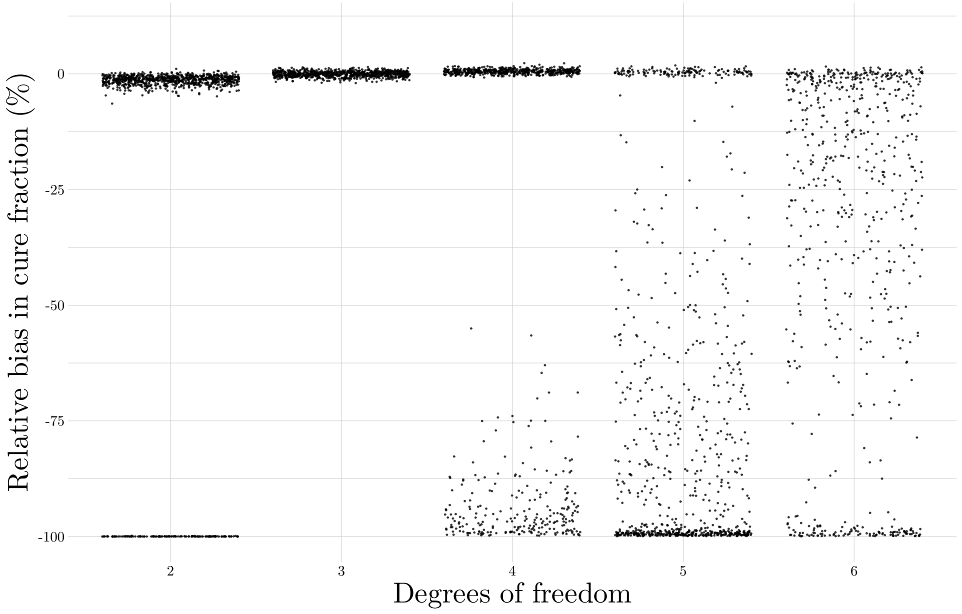

Figure 11: Relative bias in cure fraction (%) for mixture cure AFT model, baseline type 3, cure fraction 0.9.

Figure 12 (Appendix):Relative bias in cure fraction (%) for non-mixture cure AFT model, baseline type 3, cure fraction 0.9

```
# Figure 12: non_mixture_cure, sce = 3, cure_frac = 0.9
jitter <- converged |>
  filter(model == "non_mixture_cure", cure_frac == 0.9, sce == 3) |>
  ggplot(aes(x = factor(df), y = bias_relative_cure_frac, fill = df)) +
    scale_fill_viridis(discrete = FALSE, alpha = 0.6) +
    geom_jitter(color = "black", size = 0.3, alpha = 0.65) +
    base_theme +
    theme(panel.border = element_blank()) +
    xlab("Degrees of freedom") +
    ylab("Relative bias in cure fraction (%)") +
    ylim(-2.5, 5)
# print and save
if(interactive()){print(jitter)}
# figures look better in saved versions, please see ./results/figures/
ggsave(
  "./results/figures/Figure_12.pdf",
  plot   = jitter,
  device = cairo_pdf,
  width  = 300,
  height = 200,
  units  = "mm"
)
```

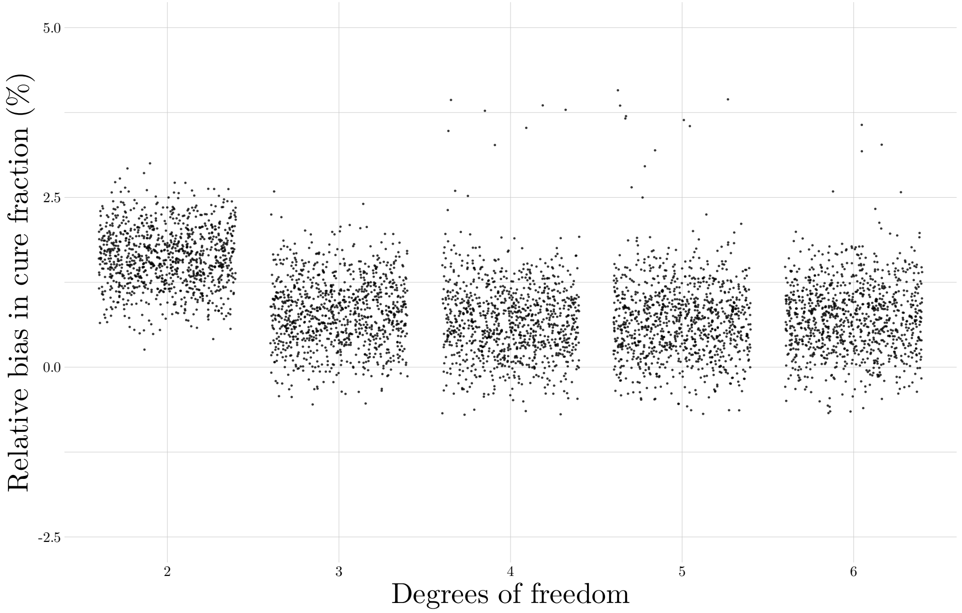

Figure 12: Relative bias in cure fraction (%) for non-mixture cure AFT model, baseline type 3, cure fraction 0.9.

Figure 13 (Appendix): Relative bias in cure fraction (%) for non-mixture cure AFT model, baseline type 3, cure fraction 0.1

```
# non_mixture_cure, sce = 3, cure_frac = 0.1
jitter <- converged |>
  filter(model == "non_mixture_cure" & cure_frac == 0.1 & sce == 3) |>
  ggplot(aes(x = factor(df), y = bias_relative_cure_frac, fill = df)) +
    scale_fill_viridis(discrete = FALSE, alpha = 0.6) +
    geom_jitter(color = "black", size = 0.3, alpha = 0.65) +
    base_theme +
    theme(panel.border = element_blank()) +
    xlab("Degrees of freedom") +
    ylab("Relative bias in cure fraction (%)") +
    ylim(-40, 350)
# print and save
if(interactive()){print(jitter)}
# figures look better in saved versions, please see ./results/figures/
ggsave(
  "./results/figures/Figure_13.pdf",
  plot   = jitter,
  device = cairo_pdf,
  width  = 300,
  height = 200,
  units  = "mm"
)
```

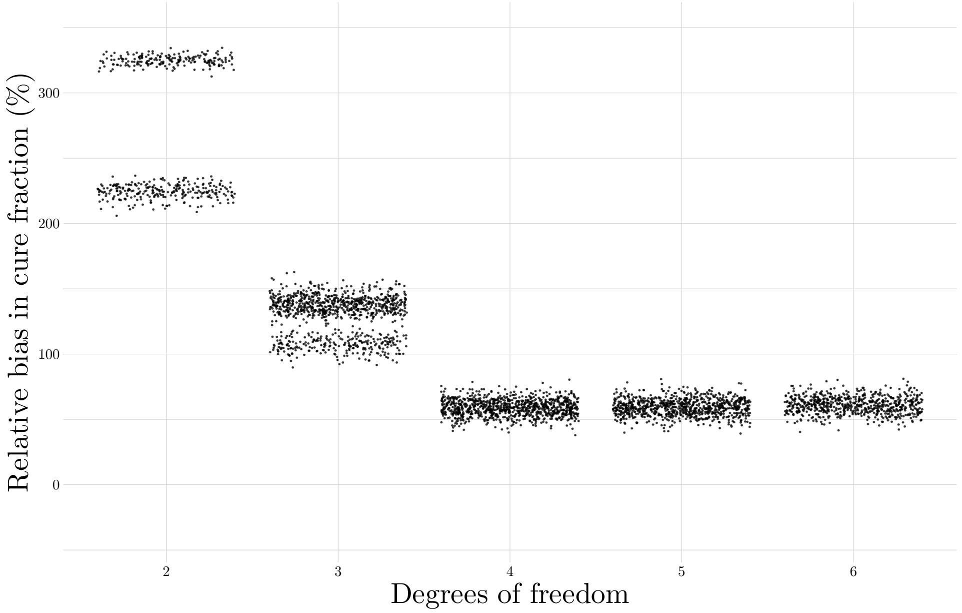

Figure 13: Relative bias in cure fraction (%) for non-mixture cure AFT model, baseline type 3, cure fraction 0.1.

Figure 14: Time-varying acceleration factor for patients diagnosed with distant colon cancer to patients without distant metastasis

```
# aft not adjusted for cure, time-varying acceleration
# factors (integrated formulation) for Distant metastasis and sex
fit <- aft(Surv(surv_mm, status=="Dead: cancer") ~ ns(age,df=2)+male+Distant,
           data=colon, df=4, tvc=list(Distant=3, male = 3),
           tvc.intercept=FALSE, tvc.integrated = TRUE)
# plot the acceleration factors
par(mfrow=c(1,2), oma = c(4,5,0,0), mar = c(1,1,6,3))
par(family = "LM Roman 10")
plot(fit, type="accfac", newdata=data.frame(age=70, male=0, Distant=0),
     var="Distant",
     main="Distant metastasis, \n Cox and Oakes \n time-dependent AFT",
     ylab = "", xlab = "")
plot(fit, type="accfac", newdata=data.frame(age=70, male=0, Distant = 0),
     var ="male",
     main="Male, \n Cox and Oakes \n time-dependent AFT",
     ylab = "", xlab = "")
mtext('Time since cancer diagnosis (months)', side = 1, outer = TRUE, line = 2)
mtext('Acceleration factor', side = 2, outer = TRUE, line = 2)
```

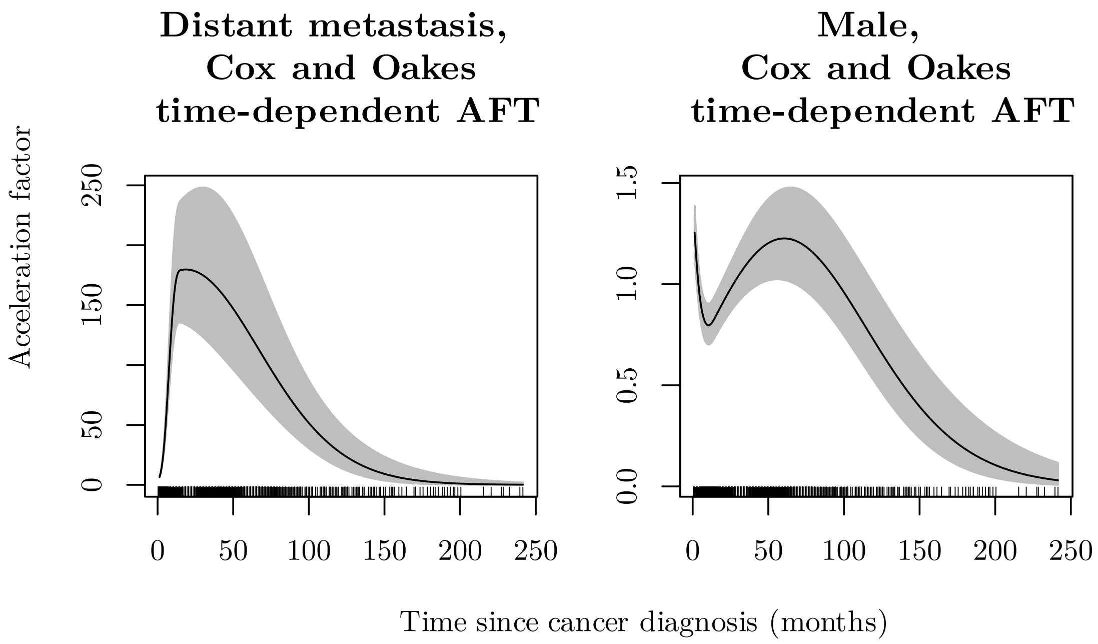

Figure 14: Time-varying acceleration factor for patients diagnosed with distant colon cancer to patients without distant metastasis (synthetic population-based cancer registry)

Session infos

Session info for this RMD

Take me back ↑↑↑

```r
sessionInfo()
```

```
## R version 4.5.1 (2025-06-13)
## Platform: x86_64-pc-linux-gnu
## Running under: Ubuntu 24.04.2 LTS
##
## Matrix products: default
## BLAS:   /usr/local/lib/R/lib/libRblas.so
## LAPACK: /usr/lib/x86_64-linux-gnu/lapack/liblapack.so.3.12.0  LAPACK version 3.12.0
##
## locale:
##  [1] LC_CTYPE=en_US.UTF-8       LC_NUMERIC=C
##  [3] LC_TIME=en_GB.UTF-8        LC_COLLATE=en_US.UTF-8
##  [5] LC_MONETARY=en_GB.UTF-8    LC_MESSAGES=en_US.UTF-8
##  [7] LC_PAPER=en_GB.UTF-8       LC_NAME=C
##  [9] LC_ADDRESS=C               LC_TELEPHONE=C
## [11] LC_MEASUREMENT=en_GB.UTF-8 LC_IDENTIFICATION=C
##
## time zone: Europe/Stockholm
## tzcode source: system (glibc)
##
## attached base packages:
## [1] splines   stats     graphics  grDevices utils     datasets  methods
## [8] base
##
## other attached packages:
##  [1] flexlsx_0.3.5     rio_1.2.3         viridis_0.6.5     viridisLite_0.4.2
##  [5] tableone_0.13.2   scales_1.4.0      tidyr_1.3.1       knitr_1.50
##  [9] kableExtra_1.4.0  flextable_0.9.7   officer_0.6.8     dplyr_1.1.4
## [13] ggpubr_0.6.0      extrafont_0.19    hrbrthemes_0.8.7  ggplot2_3.5.2
## [17] rstpm2_1.6.9      survival_3.8-3
##
## loaded via a namespace (and not attached):
##  [1] DBI_1.2.3               gridExtra_2.3           readxl_1.4.5
##  [4] rlang_1.1.6             magrittr_2.0.3          e1071_1.7-16
##  [7] compiler_4.5.1          mgcv_1.9-3              systemfonts_1.2.3
## [10] vctrs_0.6.5             stringr_1.5.1           pkgconfig_2.0.3
## [13] fastmap_1.2.0           backports_1.5.0         labeling_0.4.3
## [16] rmarkdown_2.29          haven_2.5.4             ragg_1.4.0
## [19] purrr_1.0.4             xfun_0.52               labelled_2.14.1
## [22] biostat3_0.2.2          uuid_1.2-1              broom_1.0.8
## [25] R6_2.6.1                stringi_1.8.7           RColorBrewer_1.1-3
## [28] car_3.1-3               extrafontdb_1.0         cellranger_1.1.0
## [31] numDeriv_2016.8-1.1     Rcpp_1.0.14             zoo_1.8-14
## [34] R.utils_2.13.0          Matrix_1.7-3            tidyselect_1.2.1
## [37] rstudioapi_0.17.1       abind_1.4-8             yaml_2.3.10
## [40] lattice_0.22-7          tibble_3.2.1            withr_3.0.2
```

```
## [43] askpass_1.2.1          evaluate_1.0.3         proxy_0.4-27
## [46] survey_4.4-2           zip_2.3.3              xml2_1.3.8
## [49] pillar_1.10.2          carData_3.0-5          stats4_4.5.1
## [52] generics_0.1.4         hms_1.1.3              openxlsx2_1.17
## [55] class_7.3-23           glue_1.8.0             gdtools_0.4.2
## [58] tools_4.5.1            data.table_1.17.2      ggsignif_0.6.4
## [61] forcats_1.0.0          mvtnorm_1.3-3          cowplot_1.1.3
## [64] grid_4.5.1             mitools_2.4            bbmle_1.0.25.1
## [67] Rttf2pt1_1.3.12        bdsmatrix_1.3-7        nlme_3.1-168
## [70] Formula_1.2-5          cli_3.6.5              textshaping_1.0.1
## [73] fontBitstreamVera_0.1.1 svglite_2.2.1         gtable_0.3.6
## [76] R.methodsS3_1.8.2      rstatix_0.7.2          digest_0.6.37
## [79] fontquiver_0.2.1       farver_2.1.2           htmltools_0.5.8.1
## [82] R.oo_1.27.1            lifecycle_1.0.4        fontLiberation_0.1.0
## [85] openssl_2.3.2          MASS_7.3-65
```

Session info for Time Varying Effects Simulations

Take me back ↑↑↑

```
print(sinfo_TVC)
```

```
## R version 4.4.3 (2025-02-28)
## Platform: x86_64-pc-linux-gnu
## Running under: Red Hat Enterprise Linux 9.5 (Plow)
##
## Matrix products: default
## BLAS/LAPACK: FlexiBLAS OPENBLAS;  LAPACK version 3.11.0
##
## Random number generation:
##  RNG:     L'Ecuyer-CMRG
##  Normal:  Inversion
##  Sample:  Rejection
##
## locale:
##  [1] LC_CTYPE=en_US.UTF-8       LC_NUMERIC=C
##  [3] LC_TIME=en_US.UTF-8        LC_COLLATE=en_US.UTF-8
##  [5] LC_MONETARY=en_US.UTF-8    LC_MESSAGES=en_US.UTF-8
##  [7] LC_PAPER=en_US.UTF-8       LC_NAME=C
##  [9] LC_ADDRESS=C               LC_TELEPHONE=C
## [11] LC_MEASUREMENT=en_US.UTF-8 LC_IDENTIFICATION=C
##
## time zone: Europe/Stockholm
## tzcode source: system (glibc)
##
## attached base packages:
## [1] parallel  splines   stats     graphics  grDevices utils     datasets
## [8] methods   base
##
## other attached packages:
## [1] rstpm2_1.6.9   survival_3.8-3
##
## loaded via a namespace (and not attached):
```

```
##  [1] MASS_7.3-65         compiler_4.4.3      Matrix_1.7-3
##  [4] mgcv_1.9-3          Rcpp_1.0.14         bbmle_1.0.25.1
##  [7] mvtnorm_1.3-3       bdsmatrix_1.3-7     nlme_3.1-168
## [10] grid_4.4.3          numDeriv_2016.8-1.1 stats4_4.4.3
## [13] lattice_0.22-7
```

Session info for Time Constant Effects Simulations

Take me back ↑↑↑

```r
print(sinfo)
```

```
## R version 4.4.3 (2025-02-28)
## Platform: x86_64-pc-linux-gnu
## Running under: Red Hat Enterprise Linux 9.5 (Plow)
##
## Matrix products: default
## BLAS/LAPACK: FlexiBLAS OPENBLAS;  LAPACK version 3.11.0
##
## Random number generation:
##  RNG:     L'Ecuyer-CMRG
##  Normal:  Inversion
##  Sample:  Rejection
##
## locale:
##  [1] LC_CTYPE=en_US.UTF-8       LC_NUMERIC=C
##  [3] LC_TIME=en_US.UTF-8        LC_COLLATE=en_US.UTF-8
##  [5] LC_MONETARY=en_US.UTF-8    LC_MESSAGES=en_US.UTF-8
##  [7] LC_PAPER=en_US.UTF-8       LC_NAME=C
##  [9] LC_ADDRESS=C               LC_TELEPHONE=C
## [11] LC_MEASUREMENT=en_US.UTF-8 LC_IDENTIFICATION=C
##
## time zone: Europe/Stockholm
## tzcode source: system (glibc)
##
## attached base packages:
## [1] parallel  splines   stats     graphics  grDevices utils     datasets
## [8] methods   base
##
## other attached packages:
## [1] rstpm2_1.6.9   survival_3.8-3
##
## loaded via a namespace (and not attached):
##  [1] MASS_7.3-65         compiler_4.4.3      Matrix_1.7-3
##  [4] mgcv_1.9-3          Rcpp_1.0.14         bbmle_1.0.25.1
##  [7] mvtnorm_1.3-3       bdsmatrix_1.3-7     nlme_3.1-168
## [10] grid_4.4.3          numDeriv_2016.8-1.1 stats4_4.4.3
## [13] lattice_0.22-7
```
