# Supplementary material for: Flexible Parametric Accelerated Failure Time Models With Cure
Source: Biom J. 2025 Sep 10;67(5):e70074. doi: 10.1002/bimj.70074 (PMC12423370; doi:10.1002/bimj.70074)
Supplement: Supplementary file 1 — Supporting Information [file BIMJ-67-e70074-s001.zip › code_and_data/results/figures/Figure_7-1.pdf]

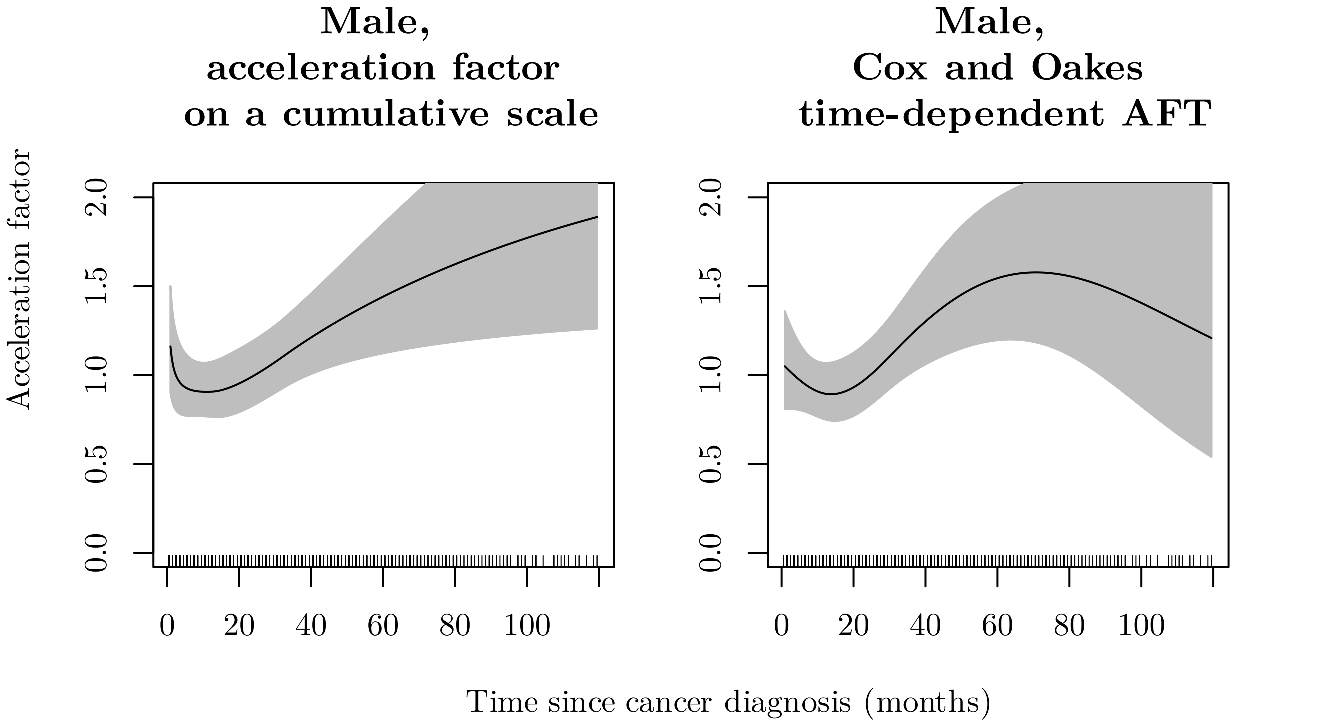
Male,
acceleration factor
on a cumulative scale
Male,
Cox and Oakes
time-dependent AFT
Acceleration factor
0.0
0.5
1.0
1.5
2.0
0
20
40
60
80
100
Time since cancer diagnosis (months)
