# Supplementary material for: Flexible Parametric Accelerated Failure Time Models With Cure
Source: Biom J. 2025 Sep 10;67(5):e70074. doi: 10.1002/bimj.70074 (PMC12423370; doi:10.1002/bimj.70074)
Supplement: Supplementary file 1 — Supporting Information [file BIMJ-67-e70074-s001.zip › code_and_data/results/figures/Figure_8-1.pdf]

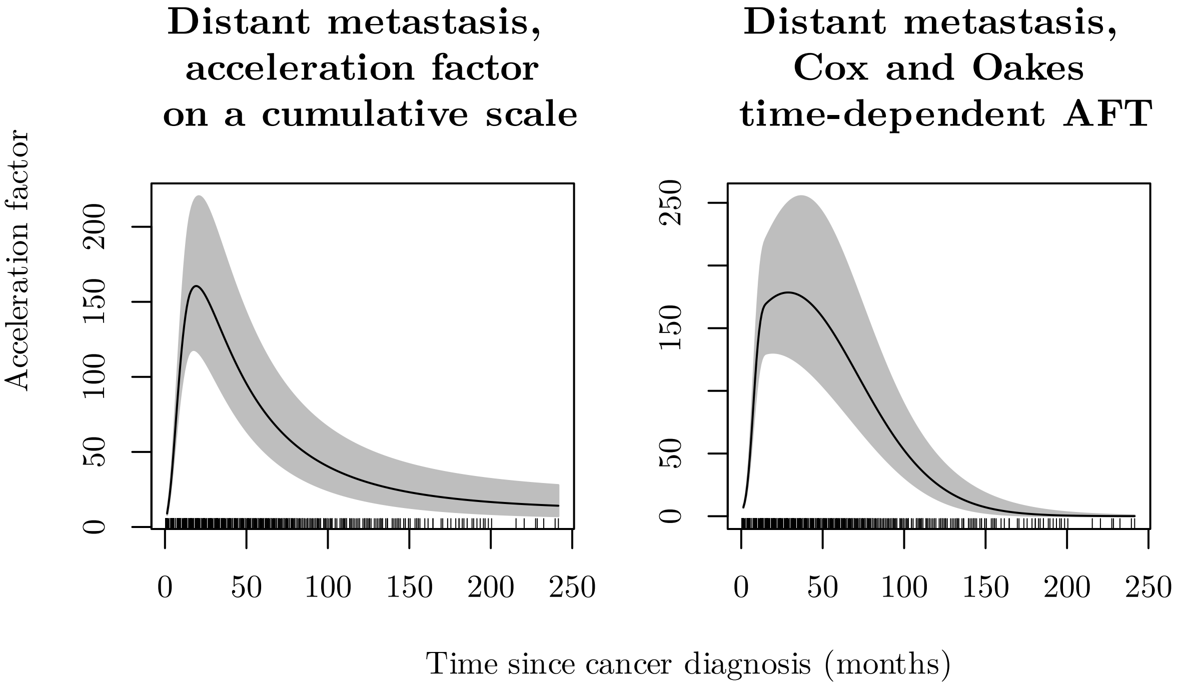
Distant metastasis,
acceleration factor
on a cumulative scale
Distant metastasis,
Cox and Oakes
time-dependent AFT
Acceleration factor
0
50
100
150
200
250
Time since cancer diagnosis (months)
