# Supplementary figures and images for: Flexible Parametric Accelerated Failure Time Models With Cure
Source: Biom J. 2025 Sep 10;67(5):e70074. doi: 10.1002/bimj.70074 (PMC12423370; doi:10.1002/bimj.70074)

**Type 1**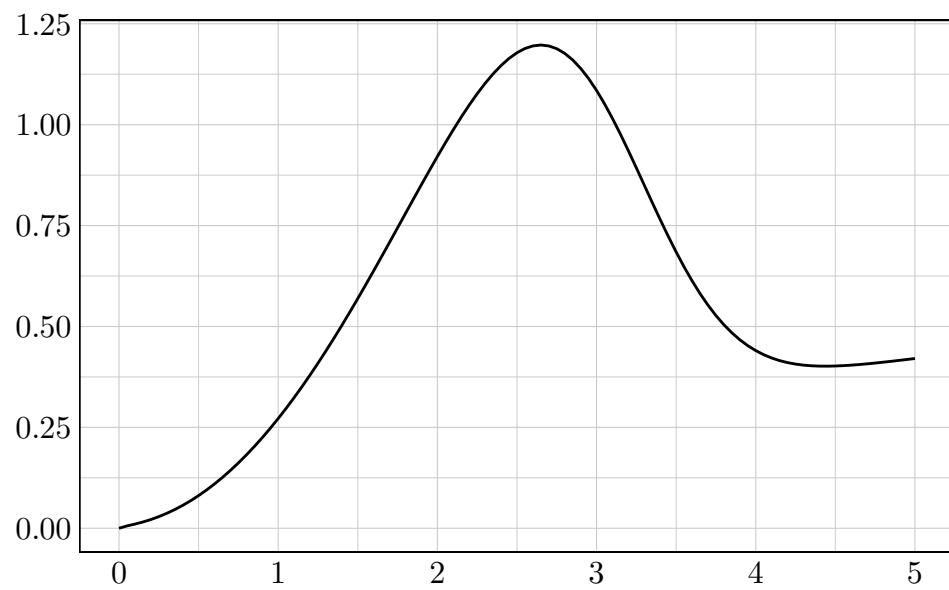**Type 2**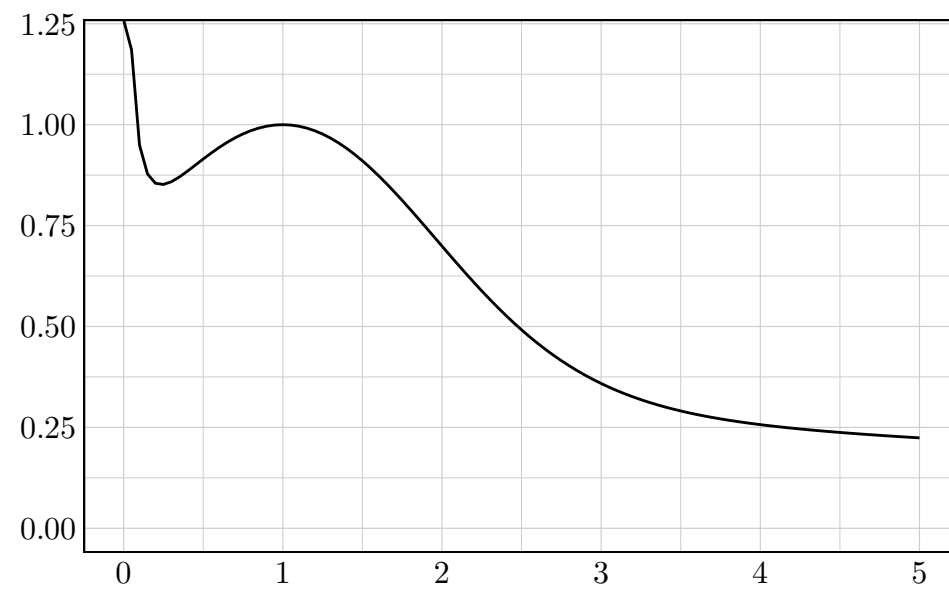**Type 3**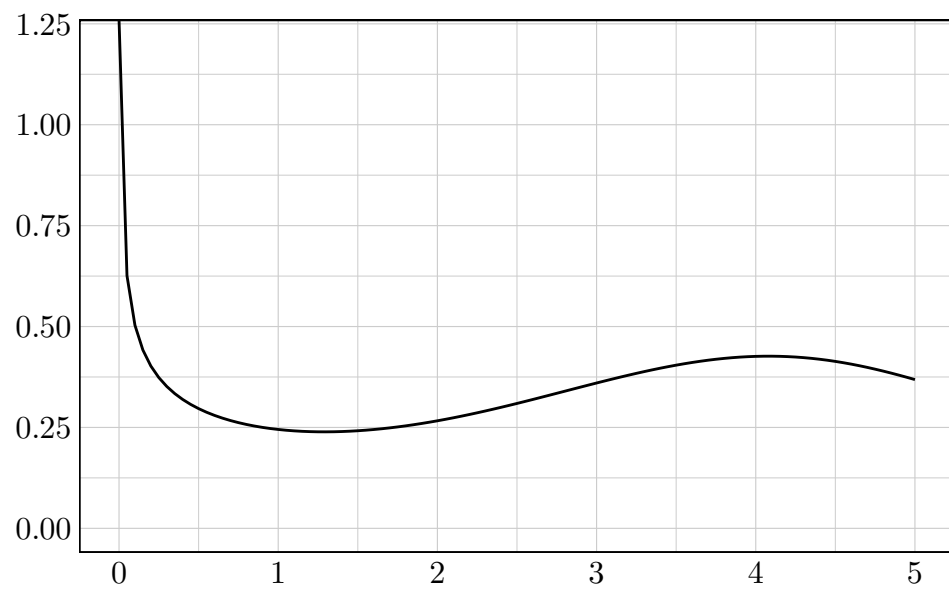**Type 4**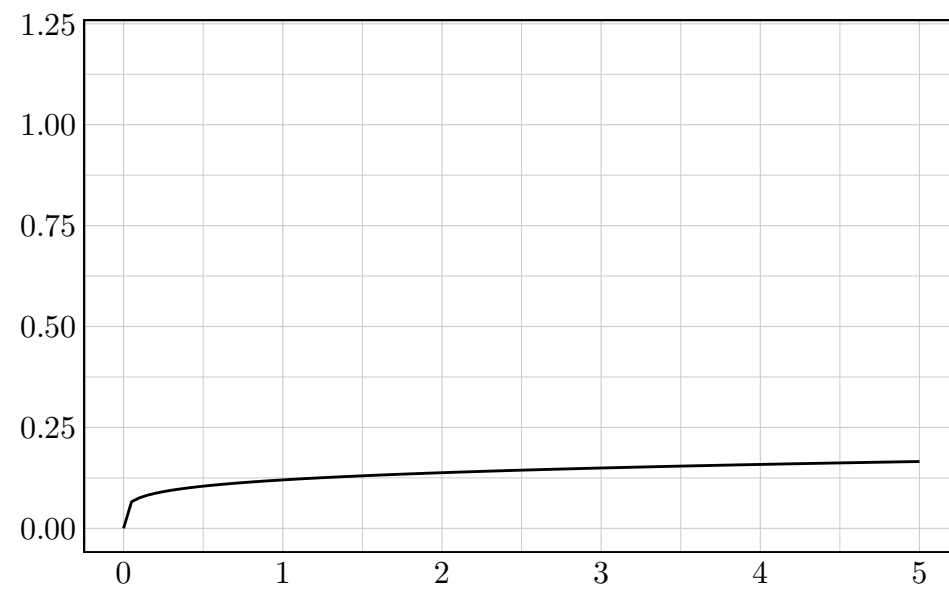

Time

Supplement: Supplementary file 1 — Supporting Information [file BIMJ-67-e70074-s001.zip › code_and_data/results/figures/Figure_1.pdf]

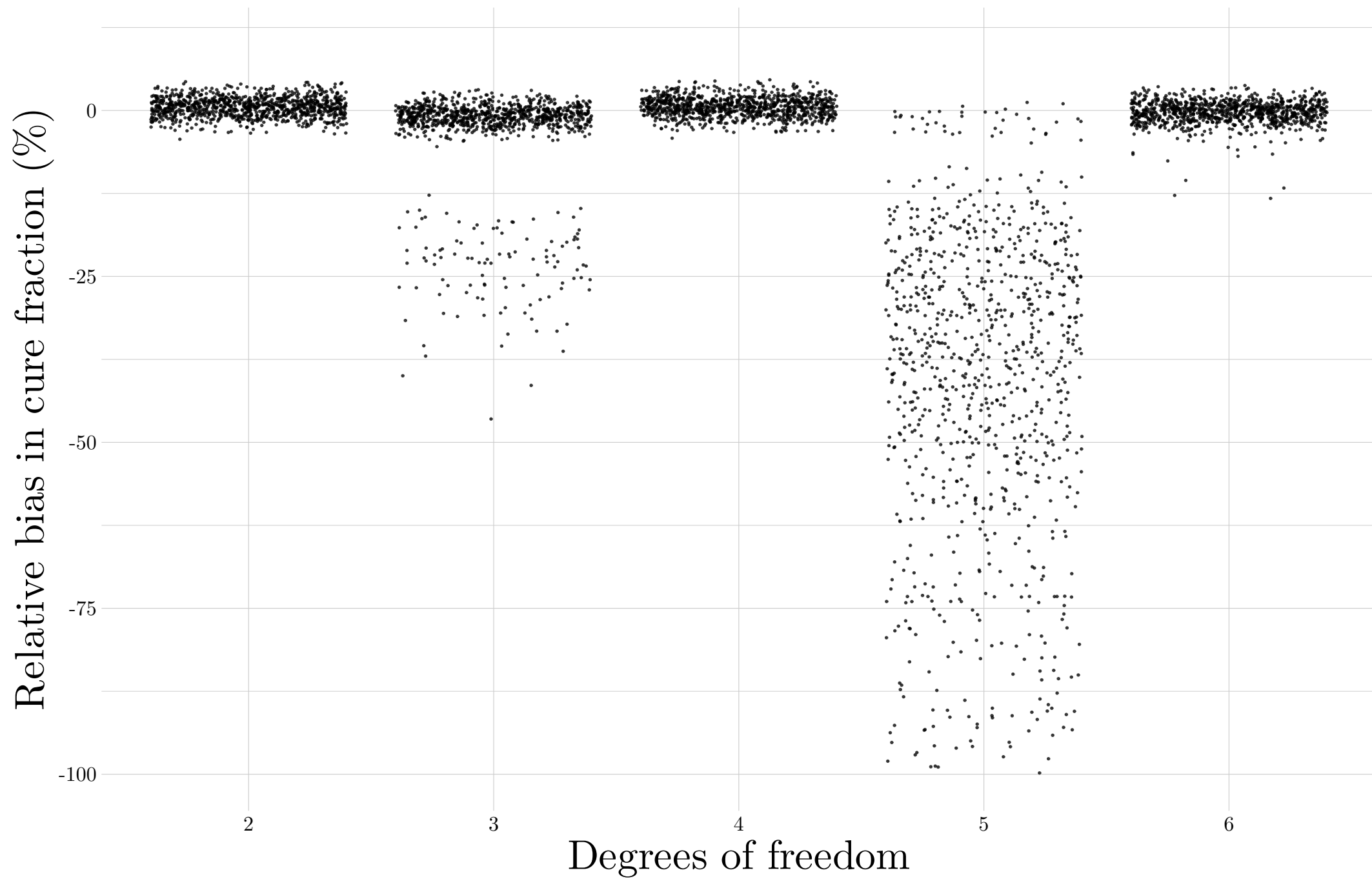

Supplement: Supplementary file 1 — Supporting Information [file BIMJ-67-e70074-s001.zip › code_and_data/results/figures/Figure_10.pdf]

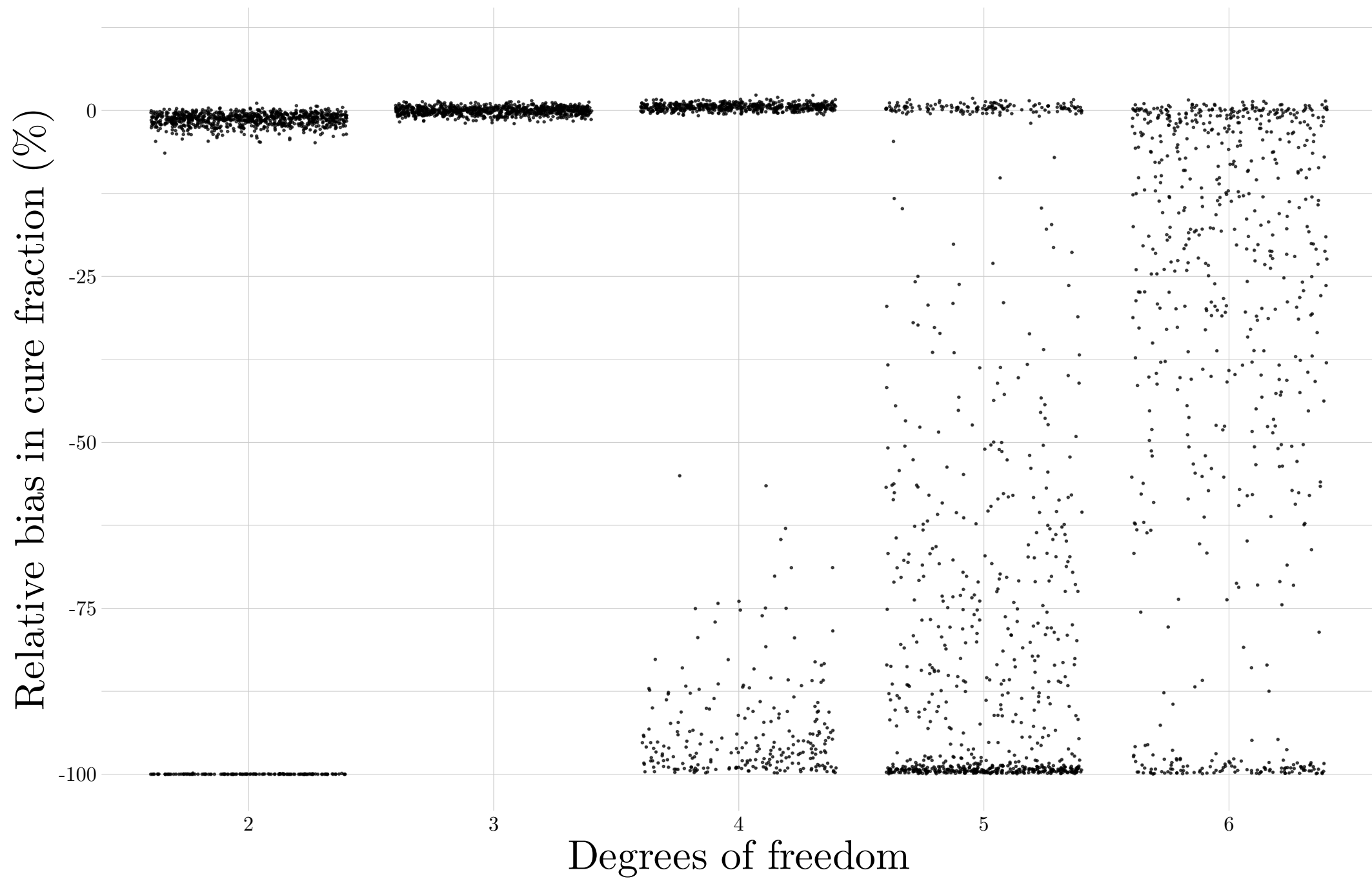

Supplement: Supplementary file 1 — Supporting Information [file BIMJ-67-e70074-s001.zip › code_and_data/results/figures/Figure_11.pdf]

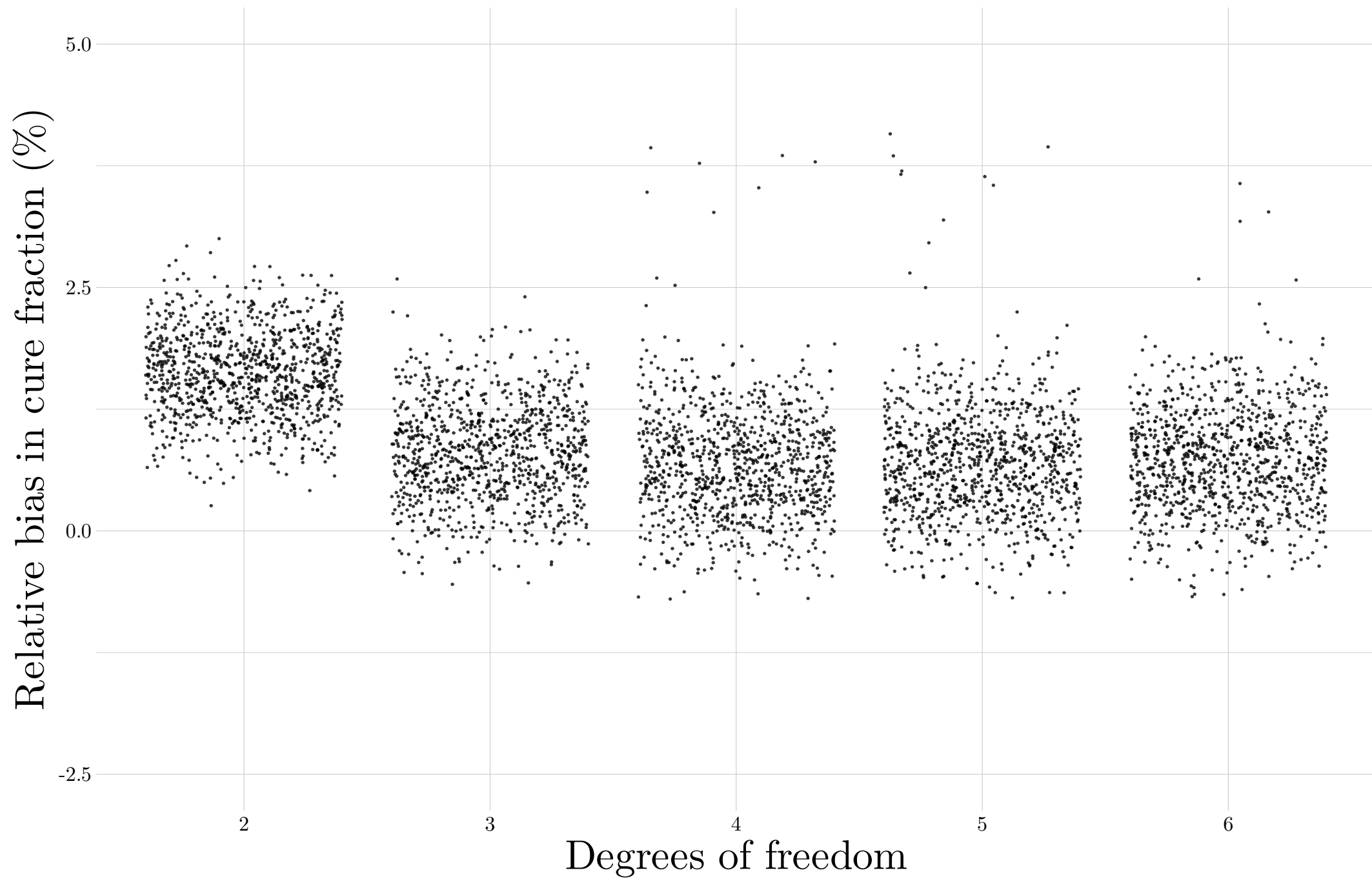

Supplement: Supplementary file 1 — Supporting Information [file BIMJ-67-e70074-s001.zip › code_and_data/results/figures/Figure_12.pdf]

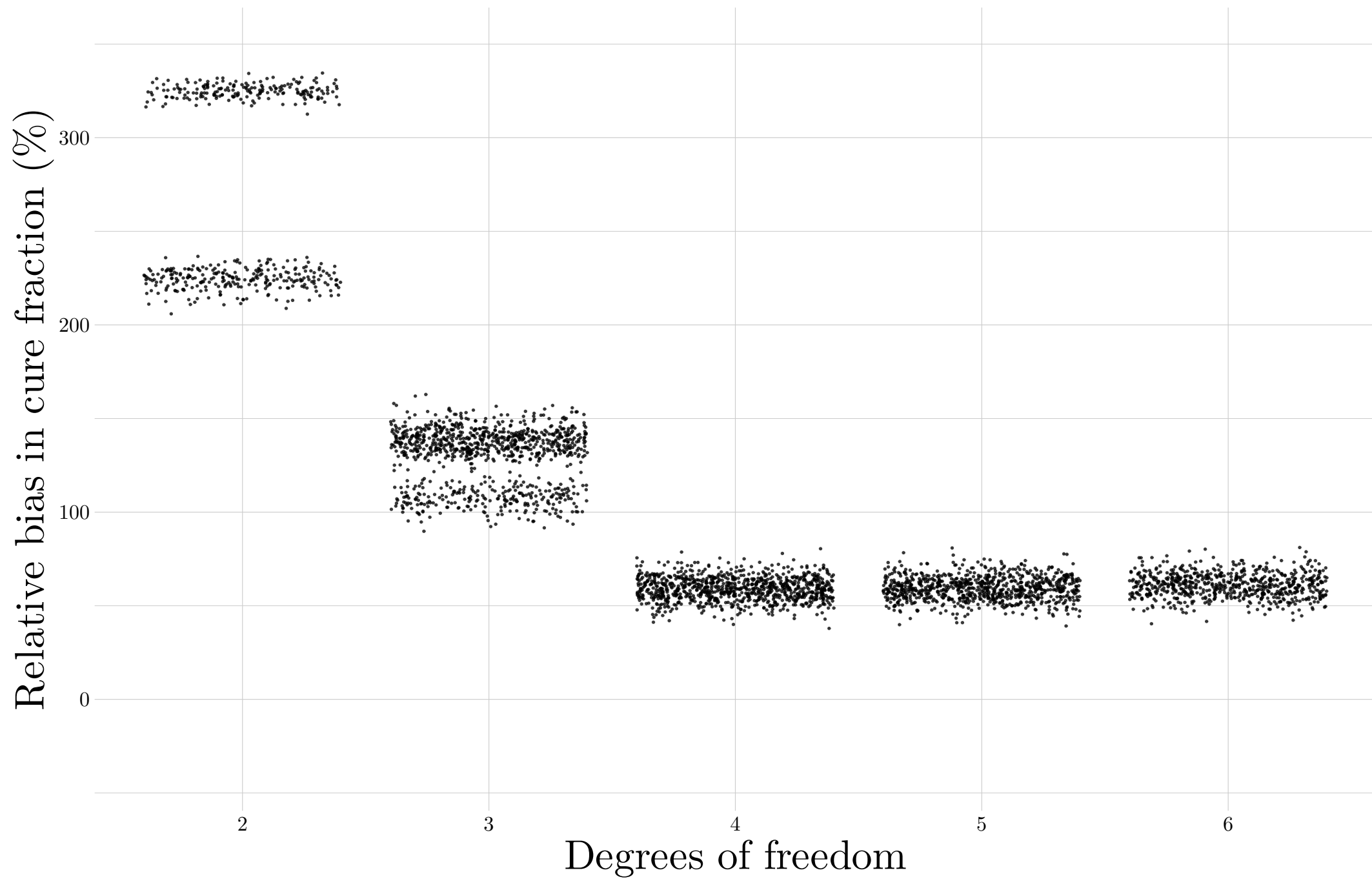

Supplement: Supplementary file 1 — Supporting Information [file BIMJ-67-e70074-s001.zip › code_and_data/results/figures/Figure_13.pdf]

Survival Probability

**Type 1**

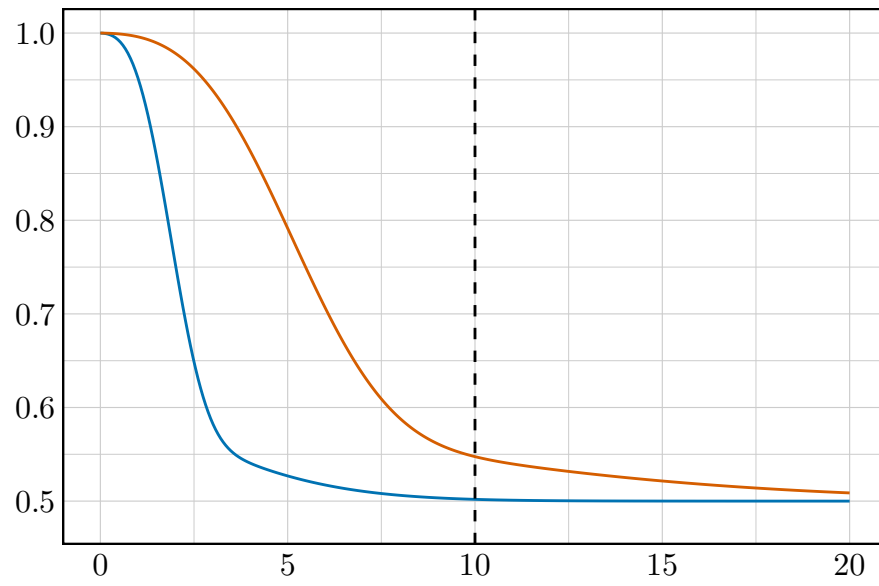

**Type 2**

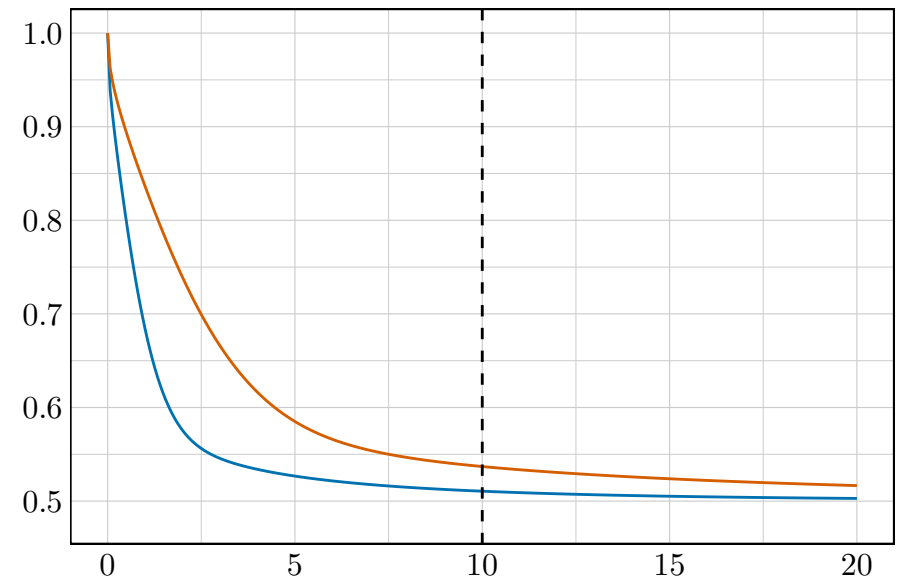

**Type 3**

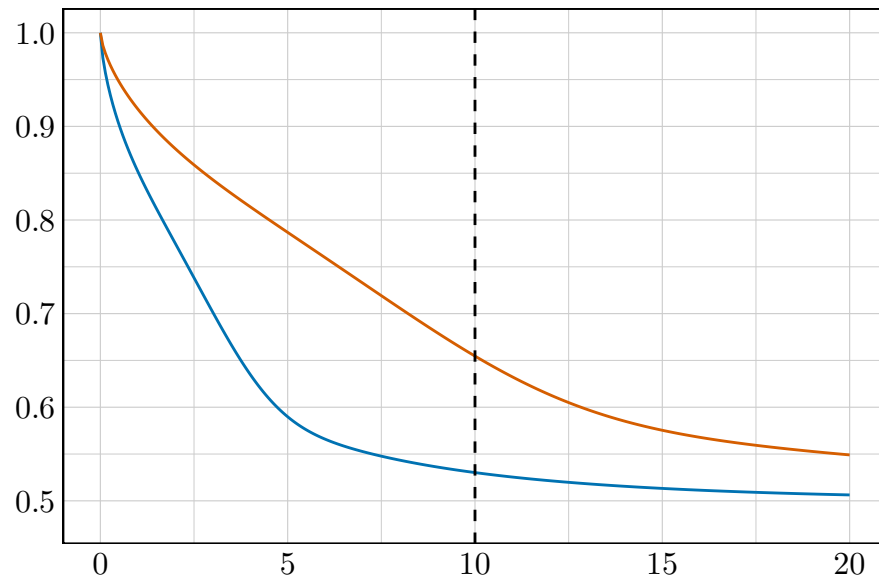

**Type 4**

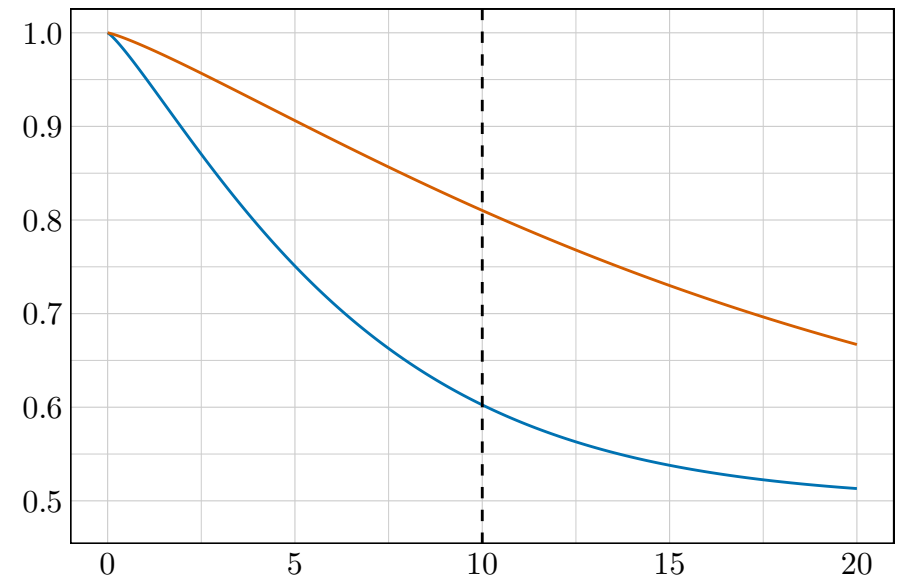

— X=0  
— X=1

Time

Supplement: Supplementary file 1 — Supporting Information [file BIMJ-67-e70074-s001.zip › code_and_data/results/figures/Figure_2.pdf]

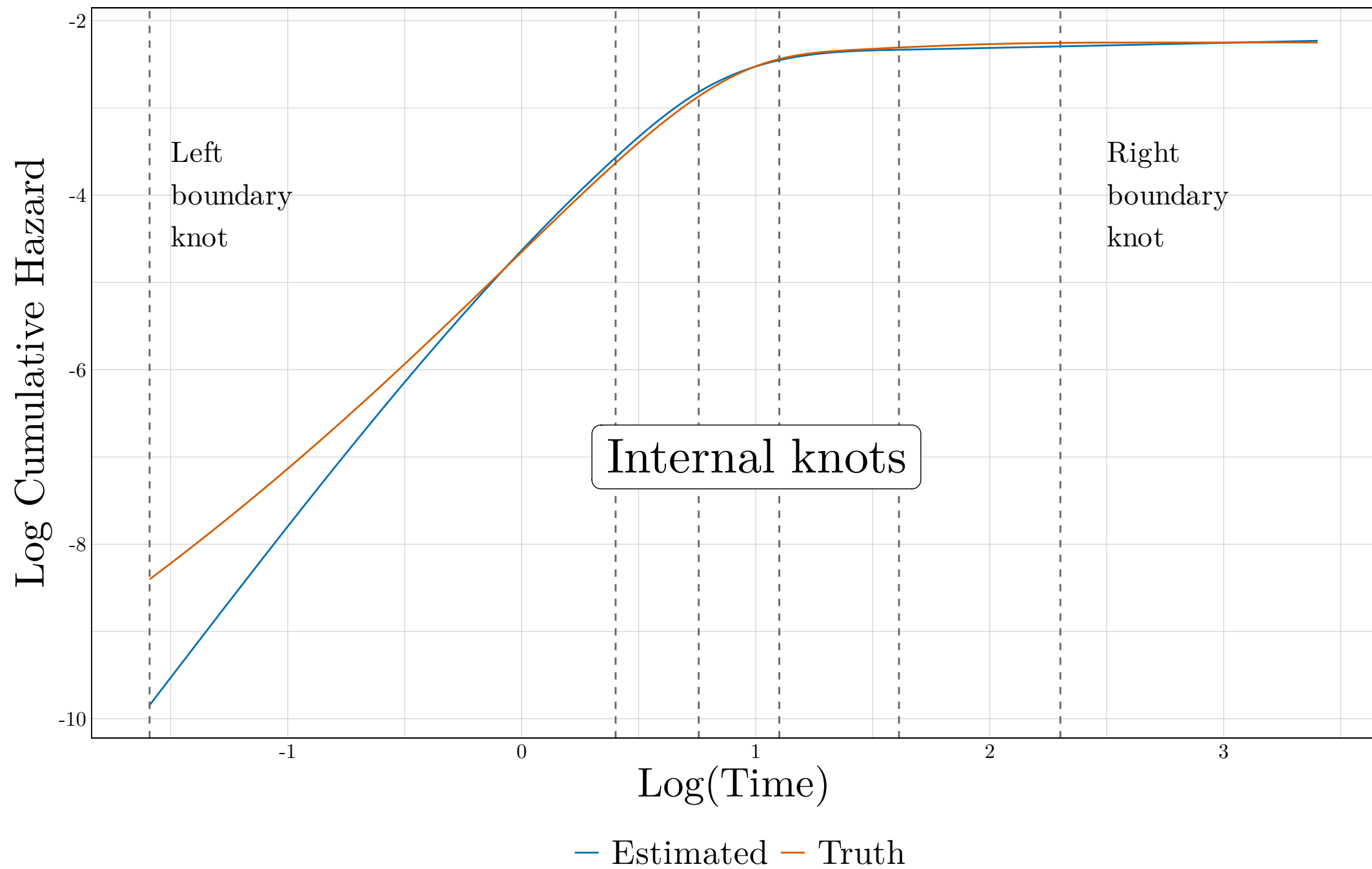

Supplement: Supplementary file 1 — Supporting Information [file BIMJ-67-e70074-s001.zip › code_and_data/results/figures/Figure_3.pdf]

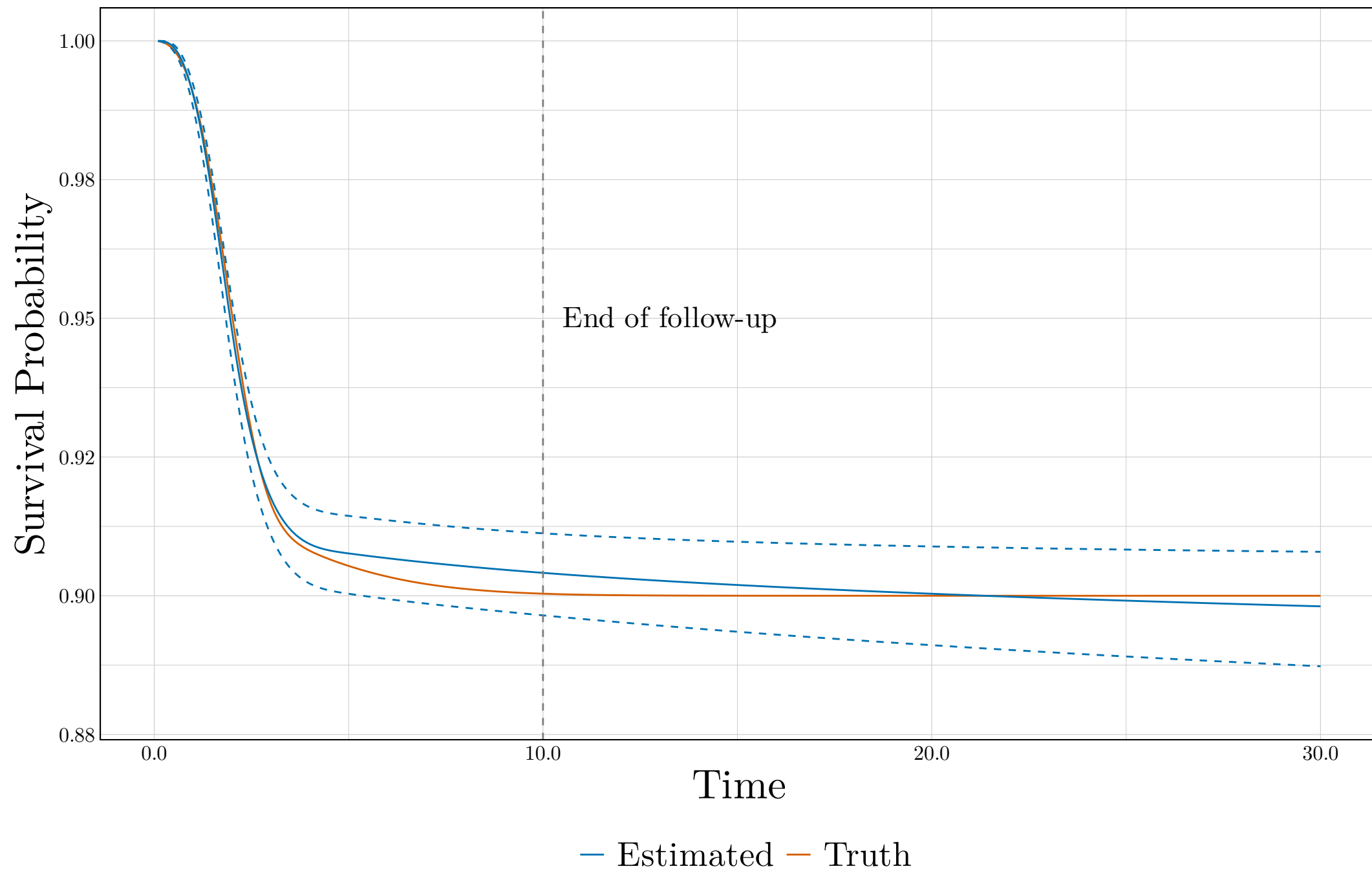

Supplement: Supplementary file 1 — Supporting Information [file BIMJ-67-e70074-s001.zip › code_and_data/results/figures/Figure_4.pdf]

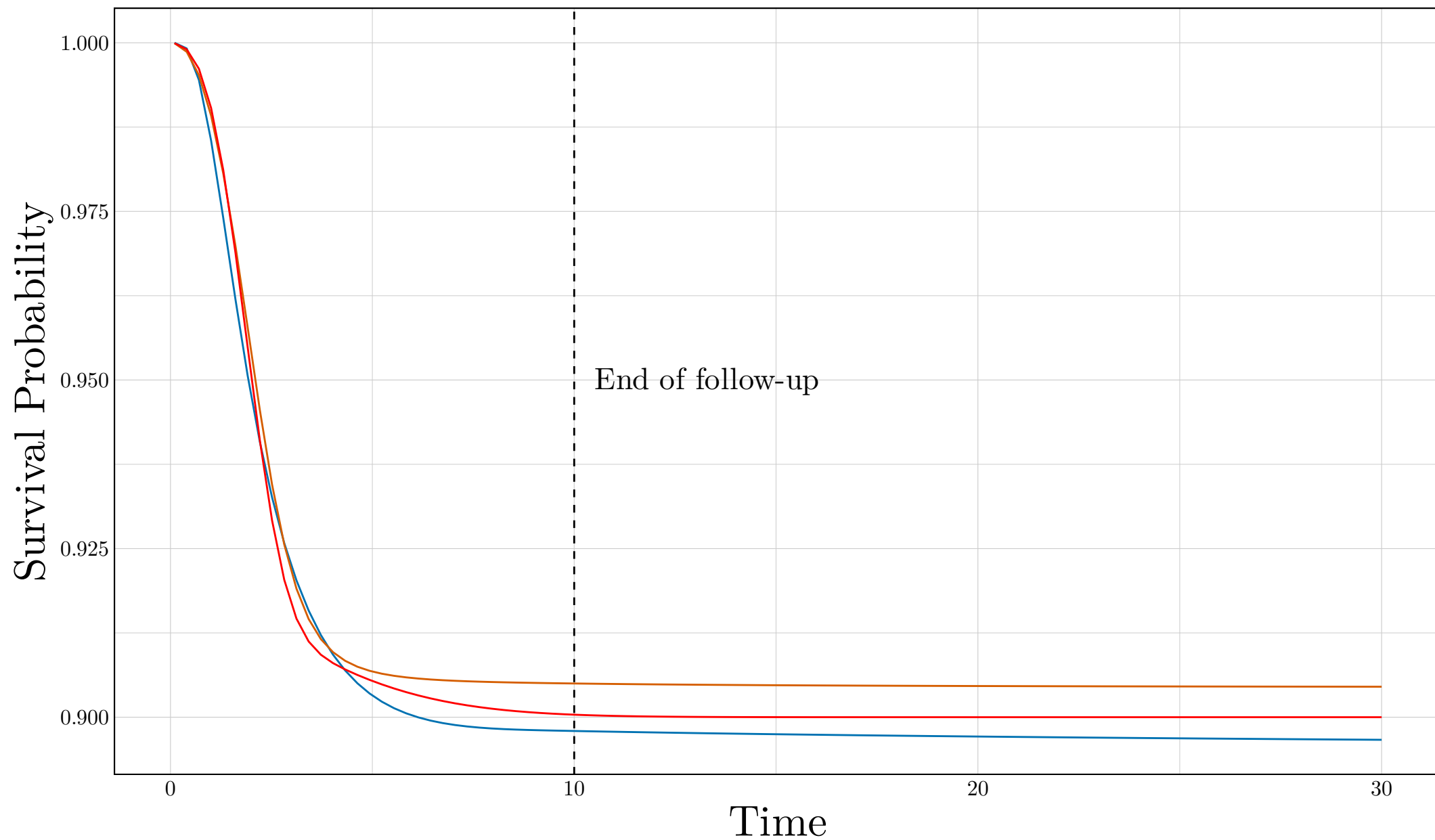

— Initial Values 1, cure = 0.36 — Initial Values 2, cure = 0.90 — Truth, cure = 0.9

Supplement: Supplementary file 1 — Supporting Information [file BIMJ-67-e70074-s001.zip › code_and_data/results/figures/Figure_5.pdf]

# Males

Survival probability

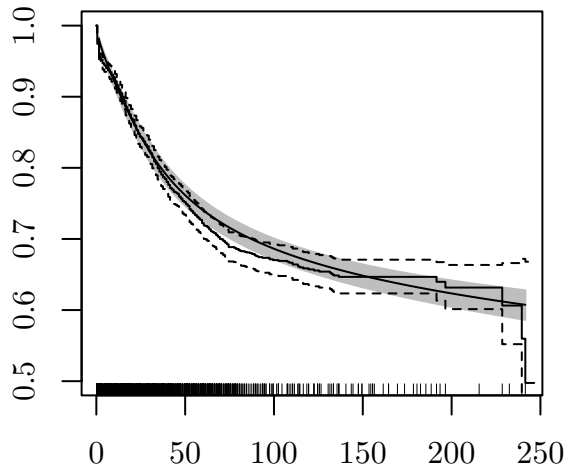

# Females

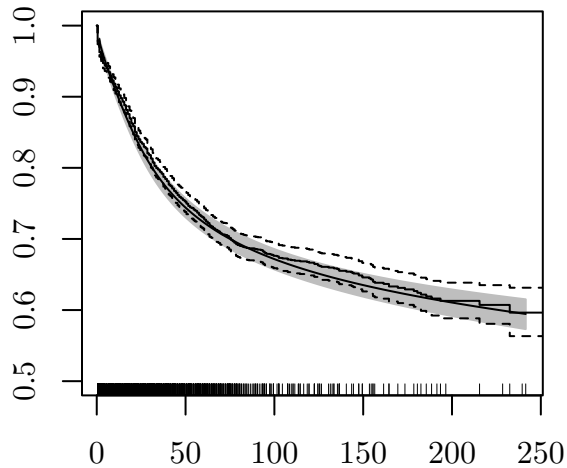

Time since cancer diagnosis (months)

Supplement: Supplementary file 1 — Supporting Information [file BIMJ-67-e70074-s001.zip › code_and_data/results/figures/Figure_6-1.pdf]

# Distant metastasis, Cox and Oakes time-dependent mixture cure AFT

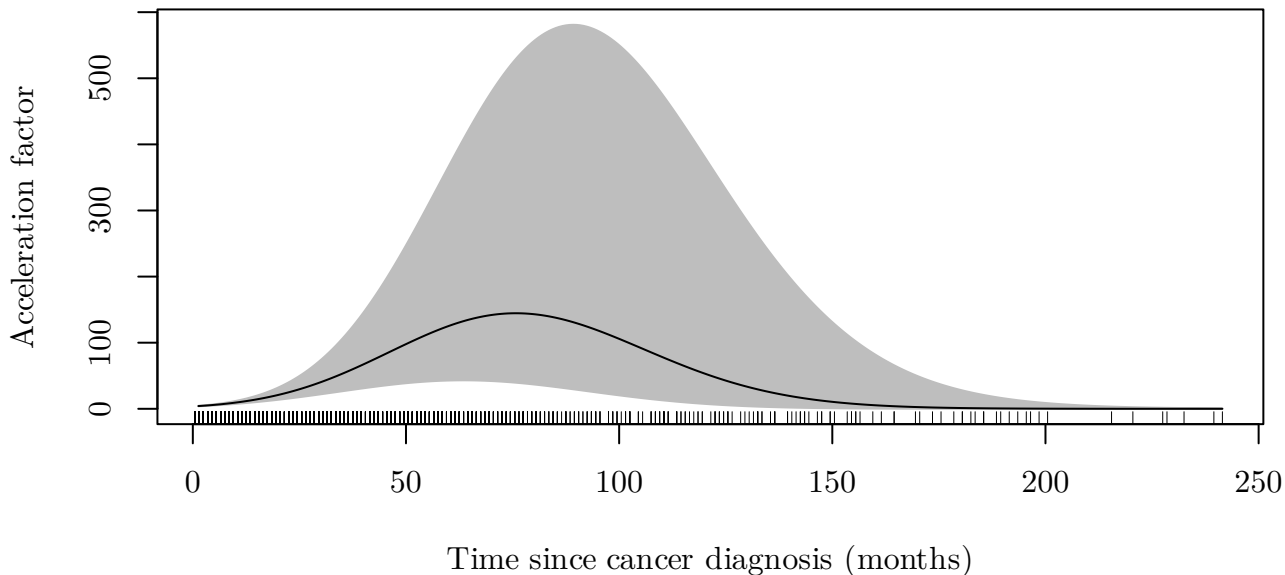

Supplement: Supplementary file 1 — Supporting Information [file BIMJ-67-e70074-s001.zip › code_and_data/results/figures/Figure_9-1.pdf]
